# The cost of reproducibility in artificial intelligence

**DOI:** 10.1016/j.patter.2026.101640

**Published:** 2026-08-27

**Authors:** Thijs Snelleman, Holger Hoos, Odd Erik Gundersen

**Affiliations:** 1 Chair for AI Methodology, Faculty of Computer Science, RWTH Aachen University, 52062 Aachen, North Rhine-Westphalia, Germany; 2 Leiden Institute of Advanced Computer Science, Faculty of Science, Leiden University, 2333 CC, Leiden, South Holland, the Netherlands; 3 Department of Computer Science, Norwegian University of Science and Technology, 7034 Trondheim, Trøndelag, Norway

**Keywords:** reproducibility, documentation, cost, quantification, replicability, meta-science, artificial intelligence, machine learning

## Abstract

Insufficiently documented publications can make independent reproduction an unnecessarily laborious task. We define the cost of reproducibility as the resources required to reproduce the findings of a study and we propose five dimensions of cost, using objective characteristics such as code and data. We review 918 publications between 2022 and 2024 at top AI venues to evaluate the variation in reproducibility costs, with the hypothesis that publications at venues with stricter standards are less costly. Papers published at machine learning conferences are less costly to reproduce than publication from other venues, 12.91% less costly compared with journals, and 16.52% less costly compared with general AI conferences. Award-winning papers, however, are not less costly to reproduce than regular papers, and 6.72% of implementation repository links were broken. These findings suggest that reproducibility costs can be significantly reduced through compliance-based mechanisms—provided they receive community support. We recommend that venues require the inclusion of reproducibility checklists in their publications, permit appendices for comprehensive documentation, and adopt low reproducibility cost as a key criterion for awards.

## Highlights

• We analyze 918 AI publications between 2022 and 2024 for their reproducibility
• Papers published at ML conferences are overall easiest to reproduce
• Award-winning papers are not easier to reproduce than “regular” papers
• We find a dark pattern of 6.72% of URLs to code repositories being broken

## The bigger picture

The reproducibility crisis is affecting empirical research across many fields of science, including artificial intelligence. As reproducibility is widely considered to be a cornerstone of the scientific method, it is of paramount importance that this crisis is mitigated if research is to progress as efficiently as possible. The quality of the documentation supplied by authors of scientific publications plays a crucial role in the reproducibility of their work. In this article, we consider this issue from a different angle; we consider the lack of documentation elements as an issue that increases the *cost* of reproducibility—i.e., the amount of (time) resources required for successfully reproducing a published scientific publication.

## Teaser

Reproducibility is widely considered a cornerstone of the scientific method and thus a key element of research, yet researchers often experience a wide variety of issues when attempting to reproduce empirical studies in AI. This work explores the relationship between the reproducibility of publications and the standards of their corresponding venues.

## Introduction

For over a decade, across all fields of science, a crisis in reproducibility of research has been a persistent problem,^1^ ranging from social sciences^2^ to physics,^3^ chemistry,^4^ biology,^5^ medicine,^6^ and other fields including artificial intelligence (AI).^7^^,^^8^^,^^9^^,^^10^ Recently, Gundersen et al.^11^ attempted to reproduce the 30 most cited AI studies and found an average reproducibility rate of 50.0%, whereas Raff^12^ estimated the reproducibility rate for machine learning (ML) studies to be 63.5%. This is shockingly low, yet not an unsolvable problem. In the social sciences, a crisis of reproducibility was assessed in 2009 by Schmidt,^2^ reporting that reproducibility is “of major importance and highly respected,” yet rarely discussed. In 2023, Korbmacher et al.^13^ determined that replication improved in the social sciences through positive structural, procedural, and community-driven changes.

Reproducibility is paramount in science, being widely seen as a “cornerstone of the scientific method”^6^^,^^14^; Popper^15^ states that “non-reproducible single occurrences are of no significance to science.” The first recorded issue of reproducibility dates back to the 17th century and is described by Robert Boyle in Shapin and Schaffer^16^; an interaction between Boyle, Hobbes, and Huygens concerning an experiment where Huygens observed an “anomalous suspension” of water in a vacuum follows:

Unless the phenomenon could be reproduced [. . .] then no one [. . .] would accept the claims [Huygens had] made [. . .]. The accomplishment of replication was dependent on contingent acts of judgment. One cannot write down a formula saying when replication was or was not achieved.

Only two machines existed at the time to reproduce this experiment—one in Boyle and Hobbes’ possession and the other in Huygens’. Regardless, Boyle and Hobbes were unable to reproduce Huygens’ findings. The solution in this anecdote was to invite the author to their laboratory, a costly but necessary solution. They found that the “accomplishment of replication was dependent on contingent acts of judgement,” i.e., Huygens’ documentation alone was insufficient for Boyles and Hobbes to reproduce the experiment. Requiring the presence of the original authors is not only a cost-ineffective solution, it is often unrealistic as well; to repeat this scenario today with Huygens’ presence would be impossible. Hence, it is obviously necessary to consider reproducibility while documenting scientific findings.

In this work, we consider the cost of reproducibility—the resources required for reproducing the findings of a prior study—and aim to quantify the cost of reproducibility for studies published at top-tier AI venues. Through the quantification of the cost of reproducibility, we seek to understand the impact and effectiveness of reproducibility standards across venues and communities on documentation practices of published AI research. Previous quantification studies on the reproducibility of AI research generally quantified the presence of documentation and their impact on the reproducibility of studies,^8^^,^^11^^,^^12^ but have not accounted for the impact on the increased demands of efforts for the independent investigators. We constructed a rubric score as a proxy for reproducibility cost—based on objective characteristics in the documentation—and conducted a large-scale systematic review of published articles spanning seven major venues between 2022 and 2024.

It would be natural to expect that venues with stricter reproducibility requirements and standards lead to publications that are less costly to reproduce for the independent investigators. Based on our evaluation of the reproducibility cost of 918 AI studies across five dimensions of documentation, we found that stricter venue requirements, such as checklists, have not independently led to easier-to-reproduce articles—their effectiveness is contingent on community support. Furthermore, we find no statistically significant differences between the cost of reproducibility for award-winning papers and other publications; we make recommendations on how the community may be enabled and stimulated to write documentation that results in low reproducibility cost. We also find that externally hosted implementation artifacts are not available due to broken URLs in 6.72% of the time—we recommend venues to archive the implementation as part of the submission policy. We believe that, by understanding what drives the cost, and subsequently reducing it, the reported reproducibility rates of 50.0%^11^ and 63.5%^12^ could be significantly increased.

## Results

First, we analyze the reliability of our results. Second, we analyze the data across seven venues and five dimensions. The dataset is summarized in Table 1, which shows that the number of effective reviews (918) is lower than the total (1,061) due to purely theoretical papers. We create a supplementary dataset based on the dataset of Gundersen et al.^11^ and study the correlation between our methodology and reproduction efforts in the section “correlating cost with successful reproduction.” We sample award-winning papers specifically and analyzed them separately in the section “award-winning papers”; in all other analyses, these papers were not considered. For all statistical tests, we have used a standard significance level of 0.05. We discuss possible extensions of our data with supplementary data in the supplemental information section “enriching the data set,” such as the correlation between reproducibility cost and citation count as shown in Table S3.

**Table 1:** A summary of the collected data, where the first number represents the number of applicable reviews and the number of excluded (theoretical) papers is represented in brackets

	AAAI	IJCAI	ICLR	ICML	NeurIPS	JAIR	JMLR
2022	46 (5)	40 (10)	46 (4)	53 (5)	44 (6)	39 (11)	39 (11)
2023	48 (2)	43 (7)	47 (3)	51 (5)	48 (2)	28 (22)	39 (11)
2024	48 (2)	47 (2)	48 (2)	47 (2)	48 (2)	33 (17)	36 (12)
Total	142 (9)	130 (19)	141 (9)	151 (12)	140 (10)	100 (50)	114 (34)

We collected 918 applicable reviews out of 1,061 papers. JAIR has the highest theoretical rate (33.33%) and AAAI the lowest (5.96%). The number of reviews (including theoretical) for IJCAI is less than 150 due to a rejected review (conflict of interest), and slightly more for AAAI/ICML due to a correction of a sampling error of award-winning papers. JMLR had less than 50 publications in 2024 at the time of sampling and is thus lower than 150 in total.

### Second review

A limitation of our dataset is the lack of reviewing resources. Due to the difficulty of the task, the effort required for reviewing, and the challenge of finding proficient reviewers, initially, only a single review was acquired for each paper by our first author. To mitigate this limitation, we acquired a second review for 46 (i.e., 5.01%) of the 918 papers from independent researchers in our network of PhD candidates and post-doctoral researchers. We let our 14 volunteers select up to five papers for secondary review based on their expertise and personal interest, but were presented with a subset to select from to ensure spread across years and venues.

We calculated the inter-rater agreement using the intra-class correlation coefficient (ICC).^17^ Based on the guidelines of Koo and Li,^18^ we applied the model ICC3*k*, which calculates the reliability of our data labeling guidelines over a fixed set of *k* raters. The labels of agreement from Koo and Li^18^ range over “poor,” “moderate,” “good,” and “excellent.” Table 2 shows that implementation, data, and configuration dimensions strongly correlate among reviewers, with an excellent label. Experimental procedure has a good label, showing some disagreement but, in general, still a reliable result.

**Table 2:** The ICC over the first and second reviews with 95% CI, *p* value, and their interpreted labels

	MAD	ICC	95% CI	*p* value	Label
Implementation	0.7609	0.9727	[0.95, 0.98]	0.0000	excellent
Data	0.7826	0.9111	[0.84, 0.95]	0.0000	excellent
Configuration	0.9348	0.9306	[0.87, 0.96]	0.0000	excellent
Experimental procedure	0.7826	0.8072	[0.65, 0.89]	0.0000	good
Expertise	2.0652	0.5445	[0.18, 0.75]	0.0048	moderate

MAD, Mean Average Distance; ICC, intraclass correlation coefficient; CI, confidence interval. Labels based on Koo and Li.^18^

Expertise receives a moderate label, which shows the complexity of assessing this dimension and the disagreement among raters, indicating the need for tangible guidelines; expertise only had loose descriptions with few objective features—a design flaw unique to this dimension, as all dimensions were grounded in the availability of specific documentation such as code, data, and parameter values. Unfortunately, this deficiency did not become evident in our preliminary experimentation. The mean absolute difference shows that the reviewers vary in their assessment of expertise cost by 2.0652, a factor of 2.55 above the other dimensions. Even when accounting for human bias, this is a noisy dimension compared with the others. Thus, we omit expertise in our subsequent analyses to ensure representative and reliable results. We further analyze the reliability of our methodology, with an intra-annotator analysis over a sample size of 62, in the supplemental information section “intra-annotator analysis.” Based on the results in Figure S2 and Table S2, we find an overall strong agreement across dimensions and some minor inconsistencies that do not significantly impact our analysis or conclusions.

### Analysis per venue and dimension

In Figure 1, our data are visualized per dimension and venue. For the first two dimensions, the percentages of studies containing public implementation or public datasets (the public rate) are indicated by dashed lines. We plot percentages of studies, with each column adding up to 100%, as the absolute numbers vary slightly (see Table 2). The median and quantiles are reported in Table S4. We consider costs in the range of 1–3 to be “low,” and those in the range of 7–10 to be “high.”

**Figure 1:**
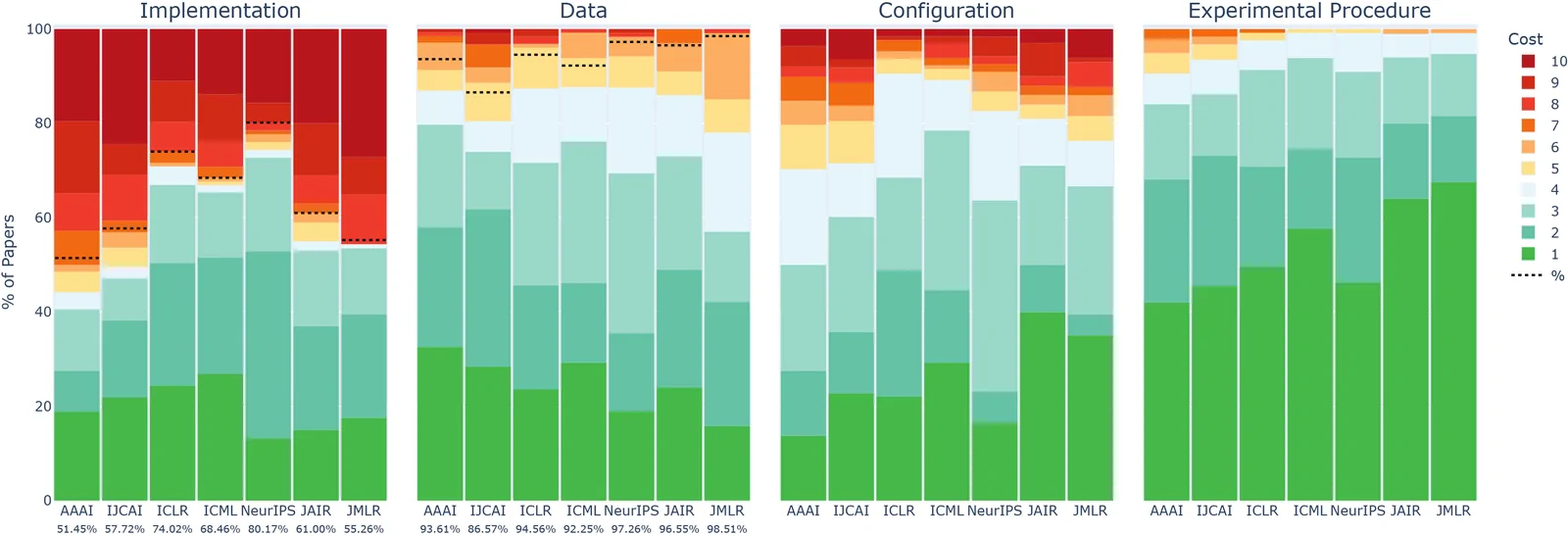
A summary of the collected dataset over seven venues The first two dimensions have their respective percentages of public code or data plotted with a dashed line; the values are denoted in the caption of each bar. Over all empirical studies, we estimate that 70.52% publish their implementation and 94.01% of used datasets are public. A tabular summary can be found in Table S4.

Figure 1 shows that the implementation dimension dominates the other dimensions; even NeurIPS, with a public rate of 80.17%, has 23.97% of studies with a cost of six or higher. Most venues have little middle ground and represent somewhat of a bimodal distribution, which is strongly demonstrated by JMLR, for example; studies either score rather low or high, and this split overlaps with the public implementation rate. This implies that studies that do not publish their implementation, in general, do not seek out to use other facets of documentation, to supplement the absence of code. The configuration dimension dominates the data and experimental procedure dimension, but has a substantially lower distribution than the implementation dimension. For all venues, the majority of studies, third quartile (Q3), have a cost of 5 or lower. The data dimension has a lower distribution, with most venues having cost of up to 4 for Q3; this is, however, not as strongly correlated with the very high overall estimated public data rate of 94.01% as may be expected. We attribute this partially to an inflation of data cost, where authors tend to deem it unnecessary to extensively document commonly used datasets. This is discussed further in the supplemental methods. The experimental procedure is the best documented dimension. All venues have a cost of 3 or less in Q3. This is relatively unsurprising; experiment documentation is an integral part of the scientific method, within the field of AI and beyond.

In order to determine statistically significant differences between venues per dimension, we applied a normality test per venue, per dimension, the results of which can be found in Table S5. We found that none of our data are normally distributed. We thus opt for a one-sided permutation test to determine which studies cost statistically significantly less than another. Statistical tests are discussed further in the supplemental methods. The results of the permutation tests can be found in Table S6.

#### Implementation

ICLR, ICML, and NeurIPS have a significantly lower implementation cost than the other venues, showing a cost reduction between −1.08 and −1.77 compared with the general AI conferences and between −0.88 and −1.44 compared with the journals. ML conferences tend to have better documented implementations than AI conferences, and papers published there are on average 16.52% less costly to reproduce. Compared with journals, we detected a cost reduction of 12.91% on average. Furthermore, we found that a substantial amount of studies follow a dark pattern: an estimated 6.72% of all implementation URLs are either empty or yield an error after up to 3 years past the publication date. This is not evenly distributed among venues: for general AI venues, the proportions range from 8.20% (JAIR) to 12.68% (AAAI), whereas ML venues range from 3.18% (JMLR) to 5.16% (NeurIPS).

#### Data

The data dimension is more costly for JMLR than the other venues, except for NeurIPS, although the measured differences are relatively small. As most venues in general do have rather low cost for the majority (Q3) of the studies, the practical significance of the measured differences seems to be low. JMLR has the highest public data rate of all venues (98.51%), yet surprisingly, it is the most costly in this dimension compared with the other venues. We attribute this partially to “popular” (benchmark) datasets. We also find that the majority of studies that rely completely on private data (3.87% of all studies) have a high data reproducibility cost: 84.85% of private data studies have a cost of 6 or higher, mainly due to the collection strategy not being clearly documented. This could be mitigated by, for example, authors creating a datasheet as suggested by Gebru et al.^19^

#### Configuration

ICLR and ICML have the lowest configuration cost compared with other venues, being significantly less costly than all other venues except for JAIR. Both the journals (JAIR/JMLR) have significantly lower cost than AAAI. The biggest, and thus practically most impactful differences, are between ICLR/ICML and the other conferences: they are on average 11.70% less costly to reproduce. The most interesting outlier here is NeurIPS, a flagship conference of the ML community, yet more costly than ICLR and ICML by 0.64 and 0.66, respectively. As shown in Figure S3, on average, there is a weak correlation between public code and lower configuration cost of −0.21 overall. However, NeurIPS has a substantially weaker correlation (−0.05), indicating that the code is used less often to document the configuration than at other venues.

#### Experimental procedure

AAAI has a significantly higher cost than all other venues except IJCAI, whereas IJCAI has a significantly higher cost than ICML, JAIR, and JMLR. NeurIPS is significantly more costly than both journals. All distances are relatively small, ranging from −0.27 to −0.65. Thus, the practical impact of the differences is low. This is also shown by the fact that each venue has a low experimental procedure cost for the majority of studies (Q3) and that no studies with a high experimental procedure cost have been found. The studies we surveyed document this dimension well, and although there is room for improvement, this is marginal compared with the other dimensions.

### Correlating cost with successful reproduction

Our evaluation has been primarily focused on the quality of documentation and its expected impact on unnecessary labor in reproducing research studies. To determine how our estimated costs directly correlate with the reproduction of a study, we rely on the dataset of Gundersen et al.,^11^ where the authors published the success rate of reproducing studies using public data within a 40-working-hour time limit. We also aimed to include a similar analysis on the results of Stodden et al.,^20^ but this was rendered infeasible due to unavailability of the data. Gundersen et al.^11^ recorded both working hours and various problem types per reproduction attempt. There are several biases to be accounted for; the authors only included studies with public data, the working hours used for reproducing research were limited to 40 per study, the studies were selected from a different time frame than the one considered here, and the studies were selected based on the number of citations, whereas ours were randomly drawn. An extensive discussion can be found in the supplemental information section “correlation with Gundersen et al. (2025).”

A straightforward approach would be to correlate our cost metric with the recorded working hours per reproduction attempt. However, the dataset of Gundersen et al.^11^ is relatively small and offers little statistical power for such an analysis; in two cases, the authors determined that no results could be acquired before the time limit, due to lacking information, thus limiting the explanatory value of the recorded labor hours in relation to the quality of documentation. Secondly, the majority of the attempts (14 of 22) ran into the time limit, both in success and failures, making the recorded working hours variable rather constant. Thus, we reframe our question toward the data as follows; how strongly are our estimates of reproducibility cost correlated with reproduction success within a limit of 40 working hours? We hypothesize that higher costs imply lower success rates; we expect to observe a negative correlation coefficient between the cost variables and success rate variables.

We applied the Kendall correlation coefficient to determine the relation between the dimensions we consider and the success rates of the reproduction attempts. We analyzed the number of experiments successfully reproduced, the number of experiments performed overall, and whether the study was considered successfully reproduced or not. We also consider the *number* of experiments completed, regardless of the outcome; the number of experiments completed is still a form of success even if the result was inconclusive, albeit less positive than the other success metrics. The results are found in Table 3. Based on our hypothesis, we expect an inverse correlation: the higher our cost, the lower the reproducibility. In Table 4 we can see that across the board, the dimensions show moderate correlations with the three properties, except for the data dimension. This is easily explainable: the data dimension has an overall low score, due to Gundersen et al.^11^ selecting studies with only publicly available data, which generally implies a low cost by our methodology. This variable is thus less insightful or predictive of successful reproduction attempts in this dataset. The eight samples of the dataset that use private data have been excluded in our analysis, as no reproduction attempt was undertaken for these studies. The implementation and configuration dimensions have moderate and significant correlations for all three metrics; implementation has a slightly stronger correlation for reproduction and successful outcome, whereas configuration has a slightly stronger correlation with regard to whether the independent investigators were able to conduct an experiment based on the documentation.

**Table 3:** The Kendall correlation coefficient between our dimensions and the recorded reproduction success on a sample dataset

	% Experiment completed	% Experiment reproduced	Success
Implementation	**−0.31** (0.03)	**−0.37** (0.03)	**−0.37** (0.02)
Data	−0.08 (0.32)	−0.01 (0.49)	0.07 (0.65)
Configuration	**−0.38** (0.01)	**−0.32** (0.03)	**−0.32** (0.05^a^)
Experimental procedure	−0.18 (0.16)	−0.16 (0.19)	−0.12 (0.28)

Based on the dataset from Gundersen et al.^11^ The second column denotes how many experiments were completed in total (including inconsistent results), and the third column denotes how many experiments of the original authors were successfully reproduced (identical or consistent results). The fourth column denotes whether the reproduction attempt was considered successful (“S” or “PS”) or not (“NR” or “F”).

The *p* values of each correlation are placed in brackets, and significant results are marked in bold.

a The measured *p* value was 0.0467, rounded to 0.05 in the table.

The Experimental Procedure dimension has no significant correlations with any of the measurements. This can be partially explained by the fact that overall, the studies in the dataset of Gundersen et al.^11^ scored quite well in this dimension (the highest recorded value was 4), similar to our results in the section “analysis per venue and dimension.” We did not expect a single dimension to show a "”near-perfect” correlation with success; lack of documentation can occur in any dimension, and the dimensions may have a joint impact on whether a study can successfully be reproduced or not. Furthermore, we found that of the problems that occurred during the study of Gundersen et al..^11^ only a few are (partially) related to the Experimental Procedure (P1, P4, P9, P10, and P13), and many reproduction attempts that encountered such a problem still resulted in successful reproduction. These problem types have a strong relation with our work and can give concrete insights into the limitations of our method, which we discuss in detail in the supplemental information section “correlation with Gundersen et al. (2025)” and expand upon in the supplemental information section “analysis on TMLR reproducibility studies” with 39 additional samples and 10 new problem types (Table S1) acquired from TMLR.

### Award-winning papers

We also reviewed 65 award-winning papers, i.e., best or outstanding papers, at AAAI, IJCAI, ICLR, ICML, and NeurIPS. JMLR and JAIR were excluded from this as JMLR does not present such awards and JAIR only has a single award-winning paper in our time frame. To test whether award-winning papers are drawn from statistically significantly different distributions when compared with “regular” papers, we applied the Kolmogorov-Smirnov test to check if they are drawn from the same distribution and the permutation test to check if the cost is significantly lower. The results are found in Table 4. Our results suggest that only IJCAI shows a single significant difference according to the Kolmogorov-Smirnov test and only ICML best papers show a significant reduction in cost for the data dimension compared with average papers, an already well-documented dimension. We find no strong evidence that the cost of reproducing an award-winning paper is lower than an average paper published at the same venue.

## Discussion

According to our results, the two most ill-documented dimensions are implementation and configuration. Gundersen et al.^11^ found that the biggest bottleneck for reproducible research in AI is the accessibility of data and code. As the data dimension is generally associated with a low reproducibility cost—driven by a high proportion of public datasets across the board—we conclude that the community has good standards in this regard. However, we do note that our study does not include a separate analysis of the documentation of datasets in the original publications, which we leave for future work. The second most important dimension, according to the findings by Gundersen et al.,^11^ implementation, is the least well documented of all. For example, 6.72% of all provided implementation links are either empty or broken. In this respect, we see room for improvement for all venues, but papers published at ML conferences appear to be significantly less costly to reproduce than those at other venues with respect to the implementation dimension.

The configuration dimension is the second least well-documented dimension, and we find that publications at ICLR and ICML are less costly to reproduce with regard to the configuration dimension than those from other venues, except for JAIR. The most noteworthy outlier here is NeurIPS, a flagship of the ML community, yet separated from ICLR and ICML. Since ICML and NeurIPS share the same checklist, yet employ a different protocol around its use, this finding suggests that the effects of venue reproducibility standards on the actual reproducibility costs are more nuanced than we initially hypothesized.

All venues employ reproducibility standards in their paper submission process (AAAI, IJCAI, ICLR, NeurIPS, ICML, JAIR, and JMLR), and the effectiveness of the different approaches is interesting: NeurIPS has the strongest requirements, and since 2024, it is mandatory for all authors to append the reproducibility checklist to their main papers published there. This is in strong contrast to ICLR, which only “strongly encourages” a paragraph-long reproducibility statement. For the year 2024, we found that 98.84% of the accepted papers at NeurIPS included the checklist in the main paper, whereas only 15.79% of the accepted papers at ICLR included the optional reproducibility statement.

However, ICLR achieves similar results as NeurIPS in terms of documentation quality with far softer requirements for authors. This difference is hard to explain based on our dataset. An important factor that could contribute to this effect is that the participants of ICLR and NeurIPS are all part of the larger ML community. Thus, standards set out by either conference chairs or its participants can easily influence other sub-communities. This effect can directly occur through resubmission: papers rejected from one conference often find a home at another one later; thus, multiple sets of standards can affect the documentation quality of a given paper. Another important factor for ICLR is its use of a public review process, allowing both reviewers and other members of the scientific community to read and respond to the submitted work. How often these broader interactions play a role in adapting the documentation of submitted work is unknown to us, as is how authors adapt their work before submission, considering that it will become publicly visible at that point. This public reviewing process only applies to ICLR and warrants an analysis of its own to determine its role in reproducibility and whether it should be recommended to other venues.

The general AI conferences have the highest reproducibility cost across the board, with a few exceptions in the data dimension. These conferences have different reproducibility standards, and most importantly, they are the only venues that do not allow for appendices in accepted publications. Appendices are frequently used by authors to document key information; we found that, for the ML conferences in approximately 69.23% of the empirical studies, the appendix contained information for one or more dimensions. However, the availability of appendices is not guaranteed to lead to better reproducibility documentation; publications at JAIR and JMLR are less costly than those at AAAI and IJCAI in terms of configuration and experimental procedure, yet the differences are marginal. We argue that the longer format enables authors to provide in-depth documentation, but that the sheer availability of them does not serve as a stimulus for better documentation. Another reason that general AI conferences are more costly than others is that they might suffer, on average, from different standards in different sub-communities in AI, which should be studied in the future.

### Limitations of the study

We attempted to evaluate the expertise required for reproducing a paper but failed to do this reliably, as described in the section “second review.” This dimension was intended to represent a more intangible cost but turned out to be impractical, due to the lack of objective guidelines enabling objective assessment. As an alternative approach, a quantification study to determine the impact of the experience of independent investigators on the effort required to reproduce a given study would, nonetheless, likely provide valuable insights, yet it remains an open question how to properly measure and quantify this experience; we leave this for future work.

Another limitation of our study is the sample size, as the process of evaluating the quality of documentation without relying on objective but simplified metrics (e.g., as done in Gundersen and Kjensmo^8^) is difficult to automate. Thus, we had to rely on human annotation for a complicated and time-expensive task; this resulted in only a small proportion of all published papers of each venue being analyzed, but we argue that the sample size of 150 per venue is statistically robust. Note that our sample size is not proportionally adapted to each venue; this has no impact when analyzing the venues individually, but it requires weighting when determining population wide trends. This is process is described in the supplemental information section “assumptions on the entire population.”

Finally, we also estimated how strong our dimensions correlate with actual reproduction attempts by Gundersen et al..^11^ We found statistically significant correlations between the two dimensions, implementation and configuration, and the success measures. One limitation of this analysis is that the dataset only contains studies with public datasets; hence, we were unable to reliably determine the relation of the data dimension and successful reproduction. Furthermore, the studies in the dataset by Gundersen et al.^11^ represent the most cited works between 2011 and 2016 and are thus less representative of the entire population of studies published within that period of time. Lastly, this dataset is relatively small, with only 22 samples, thus limiting the statistical power of our analysis, which is a common issue with meta-science studies. To mitigate the sample size limitation, we expand upon this dataset with 39 reproducibility studies published in TMLR in the supplemental information section “analysis on TMLR reproducibility studies”; we find, on the basis of Figure S1, (1) that our methodology captures 80.86% of the reported issues in reproducibility studies; (2) that contacting authors for supplementary information is unsuccessful in 36.84%, elucidating the need for comprehensive documentation; and (3) that there is a possible bias of reproduction attempts toward studies that use public data.

### Conclusion

In this work, we reviewed 1,061 papers from seven major AI/ML venues over the past three years and analyzed 918 on five proposed dimensions of reproducibility cost. We found significantly lower costs for ML conferences compared with other venues. We attribute this to the differences in venue policies—stricter reproducibility standards and longer formats of papers through appendices. We recommend that venues require the inclusion of reproducibility checklists in their submissions and allow appendices in publications to enable in-depth documentation.

However, we also find that compliance-based mechanics, such as checklists, are not effective independently, as shown by ICLR and NeurIPS as well as the general AI venues and journals; community changes are needed as well. While, in principle, increased effectiveness of such mechanics could be achieved through a stricter review process, this solution may be unrealistic in practice, as the process already demands significant time per reviewer. Venues may consider supplementing the review process by assigning a “reproducibility reviewer,” e.g., as done by the AutoML conference. This can distribute the workload and bring a dedicated reproducibility focus to the review process, and thus stronger enforcement of standards.

We found little evidence that award-winning papers are less costly to reproduce than average papers. As awards bring a spotlight to selected publications, we strongly recommend strengthening the role of reproducibility in the criteria used for adjudicating awards, to drive community changes through highly visible examples of best practices and increase the effectiveness of these measures. Alternatively, venues could include a “reproducibility papers” track, as, found in e.g., ACM MM or ECIR. Another stimulant for reproducibility is the use of workshops and challenges such as the Reproducible AI workshop or the Machine Learning Reproducibility Challenge. Hosting such tracks, workshops, or challenges places a highlight on reproducibility and enables the publication of reproduction studies.

Our study also revealed that a significant number of implementation URLs (6.72%) are empty or broken. Thus, one venue policy that may prove effective independently, is requiring authors to submit their implementation as supplementary material rather than only providing an external link in the article—archiving could reduce the erroneous implementation link rate substantially.

We hypothesized that studies published at venues with stricter requirements will be less costly to reproduce. We found only partial support for this hypothesis; although venues with stricter requirements generally cost less, we found more factors that play an important role regarding whether strict requirements are effective; community support and stimulation are important factors whether these standards affect documentation quality—without such stimulus, compliance-based mechanisms may be largely performative. Although we do not regard the reproducibility crisis in AI as solved, our work provides insight into the effectiveness of reproducibility mechanisms, and through our data-driven findings, we offer actionable pathways toward achieving positive structural, procedural, and community changes to mitigate this crisis.

## Methods

In this section, we first address and discuss the definition of the term “reproducibility” as used in this work in the section “background.” Second, we define the methodology used to obtain the results in the section “methodology.” Last, we discuss literature related to this work in the section “related work.”

### Background

Although scientists generally agree that reproducibility is fundamental to the scientific method,^6^^,^^14^ definitions found in the literature vary, and terms such as replicability and repeatability have been used as synonyms for reproducibility. Notably, over the past three decades, with the increasing prominence of computer science and automatization, there has been a tendency to define reproducibility as re-computation^21^^,^^22^^,^^23^—a definition we find too narrow, as it reduces the reproducibility of methods and results to a subset of the documentation and the execution of computer instructions. Buckheit and Donoho^24^ limited reproducibility to reproducing the figures of the original authors using their source code, which can be useful for comparison, but is only a single step in the analysis of the results, which itself represents only one part of the scientific method.

These conflicting notions were recently clarified by the Association of Computing Machinery (ACM)^25^—motivated by the lack of uniformity and clarity on the meaning of reproducibility across the field of computer science. This discussion of terminology was continued by the National Academies of Sciences, Engineering, and Medicine (NASEM),^26^ where the authors provide a comprehensive summary on the subject and overall settle on the definition of reproducibility similar to that by the ACM: “*reproducibility* is obtaining consistent results using the same input data; computational steps, methods, and code; and conditions of analysis.” While this definition provides a clear outline of the term and successfully resolves prior ambiguities, like other definitions, it introduces restrictions but more importantly omits a definition on the *objective* of reproducibility—essential in our work to understand what any “cost” is to be paid for.

In this work, we use the term reproducibility as defined in Gundersen^27^:

Reproducibility is the ability of *independent* investigators to draw the same *conclusions* from an experiment by following the *documentation* shared by the original investigators.

In this definition, the terms shown in italics are to be interpreted as follows:

*Independent*: investigators who have no conflict of interest with the original work and have no access to information limited to the original investigators.

*Conclusions*: drawing the same conclusion can be based on producing the same outcome, performing the same analysis, or arriving at the same interpretation of the analysis.

*Documentation*: any work produced, written, or otherwise, provided by the authors, including supplementary material of a publication. This can include, but is not limited to, appendices, code written or used by the authors, data, and other (digital) artifacts.

The definition of Gundersen^27^ deviates from that of NASEM,^26^ and by extension ACM.^25^ Namely, the definition of NASEM^26^ does not permit any sort of variation in data or code, which we find overly strict. Furthermore, when independent investigators draw the same conclusions as the original authors, they add *credibility* to the original method; the definitions of the ACM^25^ and NASEM^26^ do not align with this view—one can easily construct a counterexample, where different code and/or data have been used to conduct, in spirit, the same experiment as the authors and thus reproduce their findings. Thus, we conclude that the open definition of Gundersen^27^ aligns more closely with the objective that motivated this work.

Raff et al.^28^ also drew attention to the problems of confusing terminology when it comes to reproducibility, specifically within ML (we consider ML to be part of the broader field of AI^29^) research; 45% of the surveyed papers use the terms reproducibility, repeatability, and replicability interchangeably. Using the definitions of repeatability, reproducibility, and replicability of the ACM,^25^ they proposed to extend the terminology with five types of rigor: (1) adaptability, the study of applying an existing implementation to different data; (2) model selection, the study of how to conclude whether one method outperforms another; (3) label/data quality, the reliability of data acquisition; (4) meta & incentive, the study of incentives of scientific rigor; and (5) maintainability, which considers the factor of time as degradative to code. Within the definitions of Raff et al.,^28^ our work is best categorized as a meta & incentive study, as we seek to understand what factors drive the cost of reproducibility in specific venues and communities—a field of meta-science “very few papers have studied.”^28^

An extended discussion on the terminology of reproducibility used in other fields, as found in Goodman et al.^30^ and Crook et al.,^31^ is provided in the “extended background” section of the supplemental information.

#### Cost of reproducibility

In this work, we evaluate the cost of reproducibility in AI research—the resources that are required to reproduce the findings of a prior study. These resources may vary per study but will always include some time resources of the independent investigator. This demand of time resources can be significantly impacted by, for example, the absence of code. When the independent investigator must reimplement the method, the reproduction attempt becomes far more costly than a study that provides code that only has to be run. Analogously, the unavailability of data will require the independent investigator to acquire a substitute dataset or reacquire the original. This will have a substantial impact on the time resources required, thus raising the cost of reproducibility. As evidenced by the estimated reproducibility rates,^11^^,^^12^ this cost is often too high for independent investigators to practically reproduce published AI research. Here, we investigate how reproducibility cost varies across venues and communities—to understand how much effort is involved in reproducing studies from the recent AI literature. We specifically focus on top-tier venues because of the high impact of the empirical studies published there.

Based on our definition of reproducibility, we investigate the cost based on the documentation provided by the authors. To isolate this role, we design our methodology around the materials provided by the authors during the peer-review process for each venue. We formulated criteria to detect critical gaps in the provided documentation, to quantify the cost from five distinct aspects of documentation. This will enable us to determine possible trends and variations between publication venues and research communities within AI, providing data-driven conclusions to what practices should be encouraged or avoided. We describe this methodology in the section “methods.”

We are not the first to address the cost of reproducibility. Poldrack^32^ describes the costs as the efforts and struggles for early-career researchers (ECR) within the field of neuroscience to ensure the reproducibility of their work. It highlights the difficulties for ECRs to produce and write quality research that adopts more reproducible practices. These key actors within neuroscience, such as the need for resources or data as well as the impact of the “publish or perish” mentality, are present in our field as well and play a detrimental role to the reproducibility of published research. The distinction between our work and that of Poldrack^32^ is that we consider cost as the impact on independent investigators, in contrast to factors affecting authors to produce less-reproducible research.

### Methodology

As stated in the section “background,” we aim to identify criteria with regard to the documentation that correlate with the effort required for a reproduction attempt by independent investigators. We analyze it from the perspective of the original authors—costs that may be mitigated by the original authors. Factors that can increase the cost of reproducibility, such as requirements for computing resources, therefore fall outside of the scope of our analysis.

Based on the findings of previous quantification^8^^,^^11^^,^^12^ and methodological^27^^,^^28^ studies on reproducibility in AI, we propose five dimensions of documentation of AI research. Each dimension addresses discrete elements of the documentation that are orthogonal to each other and impact a different part of the reproducibility effort: (1) implementation—regarding the presented methodology and specifically how it is to be computationally executed; (2) data—regarding the input data used to obtain experimental results; (3) configuration—the algorithmic parameter settings used to produce the results; (4) experimental procedure—under which protocol the presented values were acquired; and (5) expertise—how much background knowledge the independent investigator is expected to have.

#### Implementation cost

*Given the documentation shared by the authors on a new method, how much effort would it be to re-implement the method from scratch?* The cost of implementing a method mainly considers publishing the source code used for the results of the study. By publishing the source code or the method as part of a publicly available software package, the authors can lower the cost of re-implementation and facilitate running the same or similar experiments and enable independent researchers to determine possible improvements in computational efficiency or alternative methods. Publishing the implementation can also be substituted, augmented, or enhanced with other materials, such as pseudo-code, practical descriptions, or designs and diagrams.

#### Data cost

*Given the data description in the documentation, how much effort would it take to either find the same dataset the authors used, or a similar dataset and defend the comparability, or acquire one from scratch?* The cost of acquiring data when reproducing a method and results is one of the most persistent problems in the reproducibility of AI research. We aim to evaluate how well authors document which (publicly available) data they used, to be able to conduct a comparable experiment. Furthermore, we acknowledge that publishing data is not always possible, e.g., in fields such as medicine. However, the method of data acquisition should be clearly documented, such that independent investigators can explain the comparability with other pre-existing datasets or acquire new data with similar characteristics.

#### Configuration cost

*Given the (hyper)parameters, including semantic parameters, of the method, how much effort would it take to acquire the algorithm configurations used for obtaining the reported results and compare them against their computation budget?* The acquisition of algorithm configurations and hyperparameter values is mainly a computationally costly endeavor, and the budgetary constraints under which they were achieved can yield substantial variation in the results and subsequent conclusions. By documenting the used values and under which conditions they were acquired, independent investigators can reproduce results efficiently, and defend why these results can be used to conduct a comparable experiment. This includes documenting semantic parameters, e.g., parameters related to the task not impacting outcome quality, such as the number of output nodes in a neural network.

#### Experimental cost

*Given the experiment description in the original work, how difficult is it to set up a new experiment with the same procedure?* The cost of setting up the same, or a comparable, experiment is crucial for evaluating the method. This requires documentation on the strategy and workflow, which metrics are used, and how the results are interpreted to draw conclusions. Lack thereof may significantly impact the outcome of the experiment, leading to (statistically) incorrect results and conflicting conclusions.

#### Expertise cost

*How much effort would it take to acquire the expertise required to reproduce the work independently?* Acquiring expertise to reproduce a study may vary both on the side of the independent investigators’ experience and the complexity of the study. We unify this by considering the cost of the acquisition of the prerequisite expertise, independent of whether this has been done. This may be caused by the need for knowledge from specialized subfields, requirements of inter-domain knowledge in applications and the understanding of mathematical theory.

### Assessing the cost

To quantify each dimension of reproducibility cost, we answer the questions of each dimension with a score ranging from one to ten, where one indicates very low cost and ten indicates very high cost. For each paper, we limited the documentation to all materials supplied by the publisher (e.g., the paper itself and possible supplementary materials) and any external links directly provided there (e.g., a link to a GitHub repository). No other materials were searched for online, to ensure a fair and comparable evaluation for each publication. We decided on a positive approach for the study and assumed all required documentation was provided; thus, each review starts with a cost of **1** for all dimensions.

We evaluate each dimension based on our guidelines that cover a wide range of topics allowing for the expression of a variety of topics across studies and subfields. Implementation cost is focused on practical method documentation, such as pseudo-code, diagrams, design choices, and libraries or systems used for creating the implementation as well as verifying the code presence/quality when available. Data cost is focused on all datasets used and is averaged with a weight of one per dataset by default, but we permitted reviewers to adapt these weights on a case-by-case basis. Datasets were evaluated on their descriptions regarding their task and statistics, with ample documentation requirements regarding acquisition when private. Synthetic data or simulated environments were evaluated separately, with a focus on their implementation and parameters. Configuration cost is focused on clear and structured explanations of what the authors considered parameters of their method, using, for example, pseudo-code or tables; what values were used in each experiment; and how they were acquired. The importance of parameters is taken into account when estimating the cost. Experimental cost is focused on the metrics used and the population representation, including the type of error bars, aggregation functions, and the evaluation strategy used, along with possible parameters. Expertise cost is mainly focused on how many subfields are used and to what extent and how well these are introduced. We define a set of criteria and associated penalties and provide a summary in Table 4. For brevity, a detailed specification can be found in the supplemental information, in the section “criteria examples.”

**Table 4:** A summary of our data collection guidelines

Dimension	Subset	Description	Cost
Implementation	code	bad readme (dependencies unclear, bad examples)	+1
	code	lack of comments	+1
	code	lack of repository structure	+1
	no code	no (subset) of code given	+4
	no code	no statements on framework/libraries/languages, etc.	+4
	no code	some details on framework/libraries/languages, etc.	+[2,3]
	no code	extensive practical details including design choices	+1
	no code	statements of implementations used for their method	+0
	overview	pseudo-code, designs, or architecture missing	+[1,2]
	miscellaneous	no implementation, but other key details provided	−[1,2]
Data	meta	link to dataset not provided	+1
	meta	dataset citation not given and no link	+1
	details	dataset description lacking	+1
	details	dataset statistics lacking	+1
	acquisition	dataset (partially) private	+[1,2]
	acquisition	private dataset with unclear collection strategy	+[1,3]
	miscellaneous	dataset is only named	+[0,5]
	synthetic	code not provided	+[0,5]
	synthetic	process/task not described	+[1,2]
	synthetic	parameters not clear	+[1,3]
Configuration	parameters	no clear overview/summary	+[1,3]
	parameters	values not specified	+4
	parameters	values not clear per experiment	+[1,2]
	acquisition	strategy not clear	+1
	acquisition	budget not clear	+1
Experimental procedure	strategy	strategy not clear	+[1,2]
	strategy	strategy parameters not clear	+1
	metrics	used metrics not clear	+[1,2]
	metrics	aggregation/population not clear	+1
	metrics	population variation not clear	+1
	data	split for training/testing unclear	+2
	results	data (subset) unclear	+1

In brackets, we express a range of options to summarize various sub-criteria. Each guideline is specified per dimension and subset. Not all guidelines are applicable to any given study. When multiple datasets are used, the value is a weighted average, with weights set to one by default. Further details can be found in the supplemental methods, and complete guidelines are provided in the supplemental information.

As we aim to estimate the cost of reproducibility, solely based on the provided documentation, we measure the cost indirectly; our rubric score represents the quality and comprehensiveness of the documentation of a study. This also entails that our metrics are capturing documentation quality as a *proxy* for reproducibility itself; it is possible for a well-documented paper to be irreproducible—a simple example would be a case of fraudulently manipulated results. Nonetheless, we would expect the independent investigators to reach the conclusion of *irreproducibility* with less effort when comprehensive documentation is given. Furthermore, even with complete documentation, practical issues may still surface during a reproduction attempt, thus increasing the effort required; we study this separately in the supplemental information, in the section “analysis on TMLR reproducibility studies.”

Further details regarding the methods can be found in the supplemental methods. Our full guidelines, dataset, and code can be found in our repository.^33^

### Paper sampling

We sampled studies from five major conferences and two major journals from AI and ML over the past 3 years (2022–2024), two general AI conferences (AAAI and IJCAI), three ML conferences (ICLR, ICML, and NeurIPS), and one general AI and ML journal each (JAIR and JMLR). We selected these venues based on their broad scope, excellent reputation, and high impact on the scientific community. Per venue, we reviewed about 50 papers per year, selecting award-winning papers and sampling the remaining papers uniformly at random. We reviewed each paper according to our guidelines, noting the implementation link and the amount of (public) datasets in each work as metrics. Only regular papers were included in the sampling, e.g., excluding survey papers and position papers, as they generally do not provide an empirical evaluation. We also excluded papers that were originally present in another venue to avoid over-representation (i.e., journal versions of conference papers). Theoretical papers were included, but purely theoretical papers (e.g., no empirical evaluation) were marked and excluded from our analysis as the reproducibility of purely theoretical work poses challenges of a different nature. More details on the sampling strategy can be found in the “data collection” section of the supplemental information.

### Related work

Gundersen and Kjensmo^8^ as well as Gundersen et al.^34^ conducted a quantification study regarding reproducibility. They used 16 Boolean and categorical variables over three categories, namely method, data, and experiment, to analyze the state of reproducibility in two conferences over 2 years each. They reviewed in total 400 papers, of which 325 were applicable for the study. The variables were engineered to the quality of documentation on the method, availability of data, results, and code. Based on their degrees of reproducibility, the authors concluded that none of the papers are “fully reproducible,” but that there was evidence for a statistically significant improvement over time. Although similarities are present between our methodology and that of Gundersen and Kjensmo,^8^ we approach the problem from a distinctly different perspective—we consider the effect of the collective documentation on the reproducibility effort rather than the mere presence of individual elements such as the presence of a code repository URL or the presence of a pseudo-code. Hence, we required a more expressive representation to capture the cost of reproducibility. The resulting scores allow for a more fine-grained assessment and capture the presence of the documentation elements in the context of each study. Furthermore, we separate the experimental setup and implementation of the method entirely, whereas the experiment category of Gundersen and Kjensmo^8^ covers both. The reason for this separation is that we find these two elements to serve two fundamentally different purposes—the ability to implement or execute the presented method versus the experimental protocol under which the method is evaluated.

Raff^12^ defined 26 features over three types (unambiguous, mildly subjective, and subjective) and collected values for these from 255 ML papers, published between 2012 and 2018, that he attempted to reproduce. He collected various features, including the number of plots and tables in the paper, the availability of code, the specification of hyperparameter settings, and author responsiveness to inquiries—some of which coincide with our method. He showed the correlation between the collected features and whether the paper was reproducible and thus determined that 10 features are significantly correlated. In contrast to our study, Raff^12^ focused on papers that have already been (attempted to be) reproduced and were selected based on “historical interest,” thus making the population of papers reviewed not necessarily representative of published studies in ML, let alone the broader field of AI. Second, we aim to quantify the cost of reproducibility, whereas Raff^12^ sought to answer the question of what makes a study reproducible when no code is available and assessed them accordingly. The latter is a key methodological difference; Raff^12^ explicitly stated that he does not use source code as part of the documentation of a paper to answer his research question, whereas we argue that it is a key element of the documentation.

Stodden et al.^20^ conducted a large-scale reproducibility analysis to determine the efficacy of journal policy standards on computational reproducibility in the field of mathematical computational sciences. To measure this effect, they randomly sampled 204 scientific papers published in *Science* in 2011 and 2012, and they quantified their expected reproducibility using the standards of the Institute for Computational and Experimental Research in Mathematics workshop in 2012 (ICERM) workshop; they additionally acquired a pre-policy sample (2009–2010) to examine the difference in available artifacts. Stodden et al.^20^ deemed 56 publications “likely to be reproducible,” and randomly selected 22 for a reproducibility attempt, finding all but one to be reproducible. This outlier relied on a large community dataset and documented links for software, which was no longer available.

The standards of the ICERM largely overlap with our criteria for a low reproducibility cost: the presence of parameter settings, details on auxiliary software, availability of code with separate criteria for quality of documentation thereof, and availability of input/output data. There are two criteria that are not present in our methodology: precise statements of assertions made in the paper and a full statement or valid summary of the experimental results. Precisely stating findings and documenting under which assumptions they are made are crucial for reproducing research. We rather focus on “salient details,” key information that is easily overlooked but substantially impacts the reproducibility effort, rather than the framing of claims; this focuses on what a study concludes rather than how the empirical evaluation is documented. We separated these two distinct elements in our definition of reproducibility in the section “background.” Nonetheless, there is substantial overlap between our work and that of Stodden et al.^20^ We contacted the authors to request the data to carry out a direct comparison of our methodology with their reproduction attempt findings; however, the results of the survey in Stodden et al.^20^ are anonymized, due to restrictions by the institutional review board, rendering a direct comparison with this study and ours impossible.

In a more recent approach, Gundersen et al.^11^ presented a systematic replication of 30 AI studies (10 publications from 2012, 2014, and 2016 each). The authors selected the most highly cited publications and only included papers where either data or data and code are available, based on the reproducibility types of Gundersen et al.^35^ This yielded 22 papers to be studied, the results of which represent the reproducibility of the most impactful research. They found a shockingly low (partial) success rate of 50% within a time limit of 40 working hours. The working hours measured in their study have an important link with our work, as it is a direct expression of labor, i.e., the effort required to reproduce. The average successful reproduction, including partial success, took the authors 34 h out of a 40-h time limit. They encountered problems during this process that increased the time cost, and 17 of 20 encountered problems directly related to a lack of ambiguities in documentation. We cross-analyze the outcomes of Gundersen et al.^11^ with the outcomes of our methodology in the section “correlating cost with successful reproduction.” An extension of this section can be found in the “extended quantification studies” section of the supplemental information.

## Resource availability

### Lead contact

Requests for further information and resources should be directed to and will be fulfilled by the lead contact, Thijs Snelleman (snelleman@aim.rwth-aachen.de).

### Materials availability

There are no materials generated within this work.

### Data and code availability

We document the methodology used to produce our dataset in the section “methods” and the supplemental methods. The full guidelines, code, and dataset can be found in our repository.^33^ Original data have been deposited to Zenodo: https://doi.org/10.5281/zenodo.21032062. This includes our code used to create our tables and figures for analysis. The few parameters we use, such as the amount of re-samplings in the permutation tests, are documented throughout our paper and are included in our code notebooks as well.

## Acknowledgments

We would like to thank our colleagues from our research group, specifically Justin Dettmer, Julian Dierkes, and Roman Kalkreuth, for the valuable input regarding the data collection process setup and the following for their input regarding secondary reviews: Nick Kocher, Hadar Shavit, Anja Jankovic, Henning Duwe, Bram Renting, Wadie Skaf, Annelot Bosman, Laurens Arp, Guus Toussaint, Marie Anastacio, Anna Muenz, and Konstantin Kaulen. We would like to thank the reviewers of our manuscript for their substantial, thorough, and constructive feedback, providing us with the opportunity to improve our work. Furthermore, we would like to acknowledge financial support through the Alexander von Humboldt Professorship in Artificial Intelligence held by H.H. and the 10.13039/501100005416Norwegian Research Council under the Robust Intelligent Control project (RICO, 329730T).

## Author contributions

T.S. was responsible for conceptualization, data curation, software, formal analysis, validation, investigation, visualization, methodology, writing (original draft and review & editing), and project administration. H.H. was responsible for formal analysis, supervision, funding acquisition, project administration, and writing (review & editing). O.E.G. was responsible for conceptualization, formal analysis, supervision, funding acquisition, validation, investigation, methodology, project administration, and writing (review & editing).

## Declaration of interests

The authors declare no competing interests.

## Declaration of generative AI and AI-assisted technologies in the writing process

During the preparation of this work the authors used Google Gemini in order to search for synonyms for stylistic purposes and precise terminology. After using this tool/service, the authors reviewed and edited the content as needed and take full responsibility for the content of the published article.

## Supplemental information

Document S1. Figures S1–S3, Tables S1–S7, and supplemental methods

Document S2. Article plus supplemental information
