# Supplementary material for: The cost of reproducibility in artificial intelligence
Source: Patterns (N Y). 2026 Aug 27;7(10):101640. doi: 10.1016/j.patter.2026.101640 (PMC13674308; doi:10.1016/j.patter.2026.101640)
Supplement: Supplementary file 1 [file mmc1.pdf]

**Patterns, Volume *7***

## Supplemental information

# The cost of reproducibility in artificial intelligence

**Thijs Snelleman, Holger Hoos, and Odd Erik Gundersen**

# S1. Supplemental Methods

Due to the length of the full guidelines document, we included it in our online repository: https://zenodo.org/records/21032062.

The guidelines consist of 11 pages, including two example reviews with snippets of actual papers as examples, and explain numerous possibilities for which the cost can be increased in bullet points with phrases the reviewer may be looking for in the study. We formulated the criteria based on previous reproducibility quantification studies, such as Gundersen and Kjensmo [S1], Raff [S2] and various individual reproducibility studies, as well as personal experience. We validated the generalisability of the criteria across various subfields of AI research in a preliminary experiment, adjusting them based on encountered issues in practice. We analyse the coverage of our protocol over a set of unseen case studies — reproducibility issues identified in literature that were not considered during development of our protocol — in subsection S2.2.

## S1.1 Criteria Examples

To provide further details on the methodology and illustrate the application of our criteria, we provide examples on how they are to be applied. Each criterion of the protocol is stated with a description, followed by various examples, stating the cost increase and a hypothetical situation that may be encountered in empirical AI publications. The complete protocol can be found in the supplementary material.

### S1.1.1 Implementation Cost

**Criterion: Installation Instruction & Execution [Code Available]**

*The degree to which the source code is documented for installation and execution.*

---

**Example [+0]:** Upon code inspection, the repository contains a `README.md`, a `requirements.txt` file, and a shell script to reproduce the main results table.
**Example [+1]:** Upon code inspection, the repository contains a `requirements.txt` file, but no run instructions making it more costly for the independent investigator to use the code.
**Example [+1]:** Upon code inspection, the repository contains a `README.md` file, explaining how to call the entry point of the code, but no details on how to install making it more costly for the independent investigator to execute the code on their machine.
**Example [+2]:** The code is available but has no description on installation notes or how to run the code making it more costly for the independent investigator to get started with applying the code overall.

**Criterion: Repository Documentation [Code Available]**

*The degree to which the source code itself is documented for understanding and navigation*

---

**Example [+0]:** The repository is large, but the authors provide a `README.txt` indexing each folder such that specific files can easily be found. Methods and classes are provided with a brief but comprehensive `docstring` making everything easily accesible to the independent investigator.
**Example [+1]:** The repository structure is simple, and is easily indexed by the file or directory names, but apart from copyright `docstrings`, the code basically has no comments making it more costly for the independent investigator to understand.
**Example [+1]:** The repository structure is overly complex, and the file names are not very straightforward to understand, but each method has a clear `docstring` describing the input arguments, making the code easy to follow once the correct file has been found. This makes it more costly for the independent investigator to explore and oversee the repository.
**Example [+2]:** The repository structure is extremely nested and many files in each directory. Some may be taken from other implementations and pasted in here. Apart from a random comment here and there, the repository is a maze to navigate and understand making it more costly for the independent investigator to use and trust. 21

**Criterion: Practical details [No Code, +4]**

*The degree to which the paper documents design and implementation choices; can the article can be used as a blueprint for the code?*

---

**Example [+0]:** The authors state programming languages, but also specifically state which methods and classes they used from other specific packages to build their method on giving a good starting point for the independent investigator to implement the method.
**Example [+1]:** The article states the programming language and packages used with their version, but also mention specific issues they ran into and how they optimised their code or created a work around giving a starting point for the independent investigator but may be a costly endeavour by having to resolve certain practical issues.
**Example [+2]:** The article has some mention on the implementation details and specifically states their solution was developed using `Python 3.11` with the latest version of the `JAX` package. The independent investigator can rely on specific tools from the package but will still have incur the cost of reimplementing the entire method.
**Example [+3]:** The article makes little mention of how they executed, but states they developed their solution in `C++`. The independent investigator has a starting point for their search, but most of the work will be very costly for them.
**Example [+4]:** The article makes no mention of how they executed their method, and sticks to formal and mathematical definitions. The independent investigator to start the costly process of translating the entire methodology from definition to application from scratch. 22

**Criterion: Pseudo Code and Overviews [No Code, +4]**

*The degree to which the methodology itself is easy to oversee.*

---

**Example [+0]:** The authors have summarised their new algorithm in pseudo code in the appendix, making the stepwise procedure of the method easy to follow for the independent investigator.
**Example [+0]:** The authors provide a overview figure showing how their new Neural Network architecture diverges from previous works, functioning as a blueprint of sort for the independent investigator.
**Example [+1]:** The authors develop a new algorithm to be calculated within a Neural Network architecture. They provide pseudo code on said method, but an overview on this new architecture is not provided. The independent investigator will have pay the cost of piecing together an overview from the article before reimplementing the method.
**Example [+2]:** The authors provide no overview or summary of their methodology in a structured format, leaving the independent to the costly process of deducing everything from mathematical or formal definitions. 23

**Criterion: Alternative Implementation Documentation [No Code, +4]**

*The authors find alternative ways to document critical points of the implementation.*

---

**Example [-2]:** The authors provide not only pseudo code, but also paste simplified code examples of a specified programming language and framework/package in which they executed their method. Hence, a reduced version of the code is provided, substantially reducing the cost for the independent investigator as they can immediately start with something resembling an implementation. 24

### S1.1.2 Data Cost 25

**Criterion: Dataset**

*To which degree a used dataset is documented.*

---

**Example [+0]:** The authors use a public dataset, provide a link where it is to be downloaded and cite the original paper introducing the dataset. There is a description on what the data represents and details on the number of features and instances, making all information surrounding the data easily accessible to the independent investigator.

**Example [+1]:** The authors use a public dataset, providing a citation and a link, and state how many graphs there are in the dataset including the median amount of edges and nodes. There is no explanation on what the data represents or why it was selected for evaluation of the methodology, making it more costly for the independent investigator to understand why this dataset was selected.

**Example [+2]:** The authors describe the benchmark dataset, providing statistics and its purpose for evaluation but do not cite the source or a link where it can be found making it more costly for the independent investigator to find the right (version) of the dataset.

**Example [+2]:** The authors cite the dataset and provide a download link, but provide no other information making it costly for the independent investigator to understand the data.

**Example [+3]:** The authors code automatically download the dataset used. There is no mention on the dataset in the article except for its name making the dataset costly to understand for the independent investigator.

**Example [+4]:** The authors state they use a benchmark dataset, but other than the name, it is up to the reader to determine meta information leaving the independent investigator with a costly search and possibly many assumptions.

**Example [+5]:** The authors use a private dataset. They state the collection strategy step wise in the appendix, but provide little statistics or descriptions on the resulting dataset. The independent investigator knows how re-create the dataset, but is left with the cost of defending whether their dataset is similar (enough).

**Example [+5]:** The authors use a private dataset. They state briefly how it was collected and why it remains private, but release a subset of the private dataset for evaluation purposes. The independent investigator has some working data, but is still left with the cost of acquiring auxiliary data to supplement the private data.

**Example [+6]:** The authors use a private dataset with a summary on how it was collected. They provide some form of description of the data but no statistics on for example the resulting size. The independent investigator is left with the cost of either filling in the gaps of the acquisition and executing it, or making assumptions on the presented dataset and defending these assumptions when present results on an alternative public dataset.

**Example [+7]:** The authors use a private text sentiment dataset, with a note that it was 'sourced from a popular forum'. It is not clear how many dialogues this resulted in leaving the independent investigator with the cost to fill in the gaps.

**Example [+8]:** The authors state a description of what their private dataset represents. No other information. The independent investigator has a high cost to replace it.

**Example [+9]:** The authors state they use an internal dataset, which they only name with next-to no details, forcing the independent investigator to make a myriad assumptions. 26

**Criterion: Synthetic Data**

*To which degree generated data is documented.*

---

**Example [+0]:** The authors use a synthetic data generator to create various mathematical functions to model. They describe and visualise them in the paper, stating the exact function and parameter values. The actual generator is provided in the code, making it easy for the independent investigator to understand the process and generate new data.
**Example [+0]:** The authors use a simulated environment and cite the package they used where it is implemented. The environment does not take any parameters. The independent investigator can easily access the environment and use it.
**Example [+1]:** The authors use a public environment simulator, which is cited and described in the paper. They state how many steps they use per experiment, but not which difficulty parameters are used exactly to generate the levels leaving the independent investigator with the cost to explore or assume the values.
**Example [+2]:** The authors use a public simulator, which installs with the code. There is no information however on what the problem is to be solved by the method, leaving the cost with the independent investigator to understand what the method is actually evaluated on.
**Example [+3]:** The authors use a public synthetic data generator, which automatically installs with their code. They do not specify which parameter settings they use per experiment to create the synthetic dataset, forcing the independent investigator to pay the cost of exploring or assuming the values.
**Example [+4]:** The authors use a synthetic data generator which is included in the code, but provide little explanation and do not document the majority of the parameter values used for generation, leaving a substantial cost with the independent investigator.
**Example [+5]:** The authors use a synthetic data generator which is included in the code. They do not explain on what the generated data is supposed to represent or what parameter values must be used to generate the dataset but do provide some visualised examples allowing the independent investigator to have some insights on the generator but still leaves a costly process for re-creating.
**Example [+6]:** The authors use a synthetic data generator, providing the functions and parameter values but do not share the implementation, leaving it to the independent investigator to re-implement.
**Example [+7]:** The authors use a private environment, describing clearly how it is parametrised but not what the task is. It is not clear which value is used for the parameters per experiment. This makes the reimplementation a costly endeavour but once implemented the values to be used are clear.
**Example [+8]:** The authors use a private synthetic data generator which they state is parameter has three input parameters. They provide details on the task or on the generation process, but omit any information on the parameter values. The requirements for reimplementation are clear to the independent investigator, but a costly implementation process and parameter search remains.
**Example [+9]:** The authors use a private generator which is only named. The independent investigator is left with the entire cost to start from scratch under an array of assumptions.

**Criterion: Dataset averaging**

*How multiple datasets are to be unified into a single cost.*

---

**Example [+1]:** The authors use five benchmark datasets and provide all information except for a link. Each is represented in the main results Table 1. They are hence weighted equally as each is as costly to use for the independent investigator.
**Example [+2]:** The authors use four datasets, three of which are public datasets used to benchmark the method against other state-of-the-art methods. They provide an auxiliary experiment on their internal dataset, which they briefly describe (+8) — only to demonstrate its real world performance. Hence the majority of conclusions are made on the public dataset, leading to this dataset being weighted half of the others; $\frac{1+1+1+9}{2+2+2+1} = \frac{12}{7} \approx 2$. The independent investigator is left with some cost although most of the conclusions are accessible for reproduction.

We note that the above criterion on dataset averaging, in contrast to other criteria in this dimension, is quite subjective; nonetheless, it was deemed necessary for enabling the annotator to create a unified cost representative of the entire study. This criterion of applying different weights to the datasets was applied once throughout the study; during the secondary review, one reviewer scaled two datasets to $\frac{1}{3}$ of the weight of six other datasets. This resulted in a cost difference of one between the two annotators.

### S1.1.3 Configuration Cost

**Criterion: (Hyper)parameters**

*The explicit listing of all (hyper)parameters considered for the method.*

---

**Example [+0]:** The authors provide pseudo code stating the input parameters of their method. It is immediately clear to the independent investigator what the parameters are.
**Example [+1]:** The authors discuss the parameters used with description in text, although a structured overview is missing leaving the independent investigator with the cost to verify the parameters throughout the article.
**Example [+2]:** The authors discuss certain parameters in the text, but it is unclear whether these were all the parameters considered for the method. The independent investigator is left with the cost to search through the article, code, and possible cited works to verify the parameters.
**Example [+3]:** The authors perhaps discuss some parameters throughout the article, but a real definition is not found, leaving the costly process of extracting and assuming information by the independent investigator.

**Criterion: (Hyper)parameter values**

*The actual (hyper)parameter values used for the experiments.*

---

**Example [+0]:** The authors provide a `configuration.yaml` per experiment in the code repository, making each value easily recognisable.
**Example [+1]:** The authors provide a hyperparameter table specifying the values, although not each completely overlaps with their parameter definition/overview leaving a small cost of verification for the independent investigator.
**Example[+2]:** The authors provide a concise table stating the hyperparameters of their method, but it is unclear to which experiment they belong and a substantial amount of values are missing; the independent investigator pays the cost of filling in the gaps.
**Example[+4]:** The authors provide no structured overview on the hyperparameter values and only detail this briefly for some in text. The independent investigator is left with the cost of reacquiring the majority of the values. 37

**Criterion: (Hyper)parameter value acquisition**

*How the authors acquired the (hyper)parameter values used.*

---

**Example [+0]:** The authors state they use the parameters of previous works to remain comparable to other methods in their benchmark, citing the papers.
**Example [+1]:** The authors state they empirically chose the values in a preliminary experiment, leaving it unclear what kind of experimentation budget was used leaving the independent investigator unsure how to reliably reacquire comparable values.
**Example [+2]:** The authors state the simply the values used, leaving it unclear to the independent investigator how to pay the cost of supplementing (possible missing) values in a comparable fashion. 38

### S1.1.4 Experimental Procedure Cost 39

**Criterion: Metrics**

*The metrics used for the evaluation of the method's performance on the task.*

---

**Example [+0]:** The authors use standard metrics such as accuracy and F1-score. They also use more complex metrics for which they provide a citation or describe and denote the formula in the appendix.
**Example [+1]:** The authors use a mixture of metrics, although some require domain knowledge and are not specified. For a few they provide a citation, but for others it will require the independent investigator to survey external documentation to understand how these are calculated.
**Example [+2]:** The authors use complicated metrics which are only named; the independent investigator is left with the cost to verify their meaning and how they were applied. 40

**Criterion: Data partition**

*The specification on which data the evaluation is presented.*

---

**Example [+0]:** The authors specify they use the train/validation/test split of the dataset and denote for each result whether it was on the validation or test set.
**Example [+1]:** The authors state on which dataset the result is produced on, and state dataset is split randomly into training, validation and testing 70/10/20. However, they don't state on which split their tables and figures report on, leaving the cost of determining this to the independent investigator.
**Example [+2]:** The authors state their results are on the test set, but do not clarify how they separate the benchmark data into training and testing. The independent investigator has to assume a comparable protocol.
**Example [+3]:** The authors clearly use a training procedure in their methodology and thus implying a possible train/test split, but in the results they only state the dataset they are reporting on. The independent investigator is left with the cost of assuming the protocol and on which split to compare to. 41

**Criterion: Strategy**

*The strategy used to evaluate the method on the data.*

---

**Example [+0]:** The authors state they use $k$-fold cross validation with $k = 10$ for each dataset.
**Example [+0]:** The authors state they run each experiment with five random seeds
**Example [+1]:** The authors present the results over 'multiple seeds', but the amount of seeds is unclear. The independent investigator has to assume and defend a budget.
**Example [+2]:** The authors present the results with mean and standard deviation over a population of 5, but it is unclear *what* strategy produced this population of results. The independent investigator has to assume how to recreate the population of the experiment.
**Example [+3]:** The authors present the results with a median value and a 95% confidence interval, but do not specify under what strategy they present this population nor what the population size is. The independent investigator now has to pay the cost of searching and assuming alternatives for both. 42

**Criterion: Aggregation of results**

*Is it clear what kind of statistics the authors are presenting on their results?*

---

**Example [+0]:** The authors state their results as $a \pm b$ and denote in the caption of the table that this is the mean and standard deviation.
**Example [+1]:** The authors state their results in the table are presented with the mean but do not clarify what the variation statistics describes. The independent investigator has to pay the cost of finding a suitable replacement.
**Example [+2]:** The authors provide a graph with their results, comparing their methodology against multiple state-of-the-art methods. The graph contains a centre-line and a shaded area, but no clarification on what these represent. The independent investigator has to pay the cost of assuming and replace both aggregator and variation. 43

## S1.2 Discussion

During the study, we found that the data dimension shows somewhat of an 'artificial inflation'; when the authors used popular or 'standard' datasets, such as MNIST [S3] or CIFAR [S4], the authors did not feel the need to report detailed information on them, especially when it does not play a central role to the method. We decided against making exceptions for popular or common datasets as we found it too complicated to objectively decide what makes a dataset common or 'popular', especially when dealing with smaller subfields that may use less well known datasets to the broader community, but may be considered common in their respective subfields. Thus we did not adopt a separate procedure for these datasets. This also indicates the authors did not provide a (detailed) motivation for choosing a standard dataset for a given task.

We also note that in a few cases, the number of papers slightly varied per venue or year. This we accounted for due to one conflict of interest detected post-review, and the others due to a sampling incident regarding pre-selecting the award-winning papers, resulting in more reviews.

Furthermore, we noted one theoretical example where the implementation cost could exceed ten by our guidelines; we have not seen any instance where this occurred.

For the statistical analysis we considered many statistical tests to determine the significance between distributions, which we would like to clarify. The normality test showed that the data is not normally distributed ($p < 0.01$ for all). Using Brown-Forsythe test, with the venues as groups, we find that implementation and experimental procedure have strong evidence of non-homogeneity ($p < 0.01$), significant evidence for configuration (0.045) but not for data (0.30). We did not apply ANOVA as we would violate two assumptions: Normality and homogeneity. We applied the Kruskal-Wallis test instead: For each dimension, there is a statistical significant difference for at least one group ($p < 0.003$ for all). We considered the following tests for comparing venues per dimension:

- Kolmogorov-Smirnov: Non-parametric test, does not assume normality distribution. However, it does not have a one sided version.
- Friedman: requires repeated measures which we do not have.
- Wilcoxon signed rank: an alternative to the t-test when normality cannot be assumed, but requires paired observations
- Mann-Whitney U / Wilcoxon rank-sum: no normality assumption, does not require same size samples or paired data but assumes equal variance
- Permutation: No assumption about normal distribution or variances. For robustness, we increased resamplings to 1 000 000

We decided against p-value correction (Bonferonni) to reduce the false positive rate, as our hypothesis is per single test rather than using a family of statistical test for a single hypothesis, and instead opted for interpretation of the results for practical relevance.

## S1.3 Assumptions on the entire population

Our analysis is widely aimed at comparing venues, and hence our sampling strategy was conducted uniformly at random per venue per year, with the same sample sizes. However, in a few cases, we also presented findings across the entire population; the average public data rate, average implementation link rate and its subsequent error rate. As our sampling strategy does not take into account the proportionality of each venue to the population, we weighted these metrics to the *estimated* rates by determining the proportion of empirical publications of each venue per year and calculating their contribution to the averages accordingly. This resulted in the following value shifts in our study:

- The estimated public data rate, measured in our dataset at 93.95%, calculated to be 94.01% after weighing the sources per year.

- The estimated public implementation rate, measured in our dataset 62.13%, corrected to 70.52%% after weighing the sources per year. This is the most substantial change, due to the substantially lower rates found in JMLR/JAIR, whose impact is more limited to the overall population than other venues due to a lower amount of publications per year.

- The estimated implementation URL error rate, measured in our dataset at 6.59%, corrected to 6.72%.

## S1.4 Secondary Review

A highly important part of this work is the secondary review to determine the reproducibility of our methodology. There are a few details that we would like to highlight with regards to these results. We have acquired our secondary reviewers through our research network and considered doctoral candidates, post-docs and professors based on their proficiency. To ensure a diverse group of secondary reviewers, we used various channels to contact a population with a diverse background. For example, the research directions of our network varies strongly; among the secondary reviewers we recorded the following primary research directions: Reinforcement Learning, Neural Network Verification, Music Information Retrieval, Robustness, Algorithm Selection/Configuration, Multi-Agent systems, Time Series Analysis, SAT Solving, Hyperparameter Optimisation, Data Mining, and, ML for Earth Observation. Thus, the communities they are part of which impact their views and judgements are diverse. This was done to reflect a wide variety of researchers from the field of AI, and mitigate possible biases in this part of the dataset. We subsampled our dataset using the same strategy as our initial sampling, and ensured each venue and year received a proportional amount of reviews to be representative of the distribution of the dataset.

The secondary reviewers were allowed to select papers by title themselves based on their expertise and personal interest. This potentially introduced a topic-matching bias — but this was permitted for two main reasons; 1) expertise in a certain subfield tends to increase the consistency of a given rater across samples and 2) their interest in the domain reduces the chances of human error due to fatigue. As Bayerl and Paul [S5] pointed out, raters with similar levels of expertise may give rise to inflated agreement scores. They note that this bias, the influence of expertise on agreement, is not consistent across domains. As we do not have any information whether this bias is applicable to our domain, this remains an open question. However, the topic-matching bias only occurred on one side of the annotation — namely the secondary reviewer. As noted by Bayerl and Paul [S5]: "[..] to increase the likelihood of high agreement rates annotator groups should consist of coders with a comparable level of expertise". Hence, we argue that the topic-matching bias triggered the exact opposite effect — only one side was matched with their supposed expertise, whereas the other was sampled uniformly at random, thus not guaranteeing any specific subfield expertise.

Another important subject of this analysis is the sample size; we acquired 46 secondary reviews for our entire dataset (5.01%). Although larger sample sizes are always highly desirable in any data-driven study, we faced resource constraints due to the complexity of the task; we had strong requirements for the academic expertise of the reviewers, and conducting such a review is time consuming. We do find the sample size is sufficient for statistically robust claims, and hence do not expect this outcome to change substantially with a larger sample size under this strategy.

# S2. Extended Results

Apart from annotating a sample of publications over the past three years of seven main venues, we also validated our methodology against actual reproduction attempts. We first discuss limitations on our comparison with the dataset by Gundersen et al. [S6] in subsection S2.1, then discuss an extension of this dataset with reproducibility studies sourced from TMLR in subsection S2.2, analyse the intra-annotator agreement in subsection S2.3, and discuss the possible enrichment of meta data in subsection S2.4.

## S2.1 Correlation with Gundersen et al. (2025)

In the dataset of Gundersen et al. [S6], there are a several sources of bias that should be noted with regards to our analysis:

- The authors used publications from a different time frame (2011 to 2016). Standards of documentation have changed over the years, which may bias the data.
- The authors considered *only* publications that use public data for their experiments.
- The authors selected the most highly cited publications from their time frame.
- The authors used publications from other venues than those included in this study. Although our method is generalised towards all empirical AI studies, this may imply a shift in population as the community (standards) of researchers from these venues may vary from those evaluated in this work.
- The authors have decided on a hard cut off of forty working hours per study. Working hours also included computing time; this is not considered labour by the definition of reproducibility cost in our work.

**Limitations of our method.** Gundersen et al. [S6] measured problem types P1, ..., P20 per reproduction attempt, but not present per study in the original publication. We requested the data regarding which study encountered which problem type, which they kindly provided us with. They encountered twenty different problems, the majority of which is covered in the documentation evaluation of our method; in fifteen cases, we could determine that the subject is covered in our assessment of at least one dimension. However, five of these cases (P1, P7, P14, P19 and P20) are not covered, which amounts to 18.84% of the encountered issues. This is a limitation of our method (i.e. encountered documentation issues that are not captured by our methodology) that we would like to discuss.

- (P1) "Method code is shared, but not experiment code": Our methodology focuses on the documentation of the implementation of the newly presented method. The authors find this issue in five attempts; only one occurred in an unsuccessful attempt.
- (P7) "Random seeds and random number generators not specified": We explicitly decided against including such values into our cost assessment, as we believe that outcomes should generally be statistically robust and reproducible under repetition with different pseudo-random number sequences. Gundersen et al. [S6] found this issue in four attempts, none of which resulted in a failure to reproduce a given study. Hence we conclude that not including this into our measurement is not a limitation, but rather that our choice is supported by the (limited) data set.

- (P14) "The article contains an error": This is a very important problem, that can only be detected upon deeper inspection (and perhaps actual reproduction attempt) of a study. Gundersen et al. [S6] encountered this once, but the reproduction attempt was still successful.

- (P19) "Results are presented in a way that makes a comparison hard": This problem is focused on the presentation of the method. This is a very important topic that is hard to capture objectively. Gundersen et al. [S6] encountered this in two publications; the reproduction attempt of one turned out to be unsuccessful.

- (P20) "Lack of access to hardware or software needed to conduct the experiment": This problem is not related to the documentation provided by authors, but rather the accessibility of resources and thus not included in our method, based on our definitions.

## S2.2 Analysis on TMLR Reproducibility Studies

The correlation study between the reproducibility effort of Gundersen et al. [S6] has yielded valuable insights into the relation between our methodology and actual reproduction attempts. Although the study by Gundersen et al. [S6] has involved substantial effort to acquire data on the reproducibility of AI research, a larger population sample may afford more statistical power and achieve more generalisable conclusions. To this end, we analysed reproducibility studies from the Transactions on Machine Learning Research (TMLR). TMLR is an online open access journal (https://jmlr.org/tmlr/).

### S2.2.1 Acquisition strategy

TMLR awards special certifications to submissions aiming to reproduce a previous study, a characteristic we leveraged to manually scrape the submission website (TMLR uses OpenReview for a public reviewing process; https://openreview.net/group?id=TMLR). When collecting possibly relevant studies, we specifically included in our sample studies that have been rejected; without, this may introduce a bias towards certain studies, for example failed reproduction attempts that were submitted to TMLR. However, to ensure we only rely on methodologically sound reproducibility studies, we selected them based on the feedback of the reviewers and the editor. Hence, only those rejected due to a 'lack of novelty' — as reproducibility studies are required by TMLR standards to go "beyond simple verification" and to "contribute significant added value through additional baselines, analysis, ablations, or insights." (See; https://jmlr.org/tmlr/editorial-policies.html) — were excluded from our dataset.

This resulted in 39 reproducibility studies published in TMLR between 2024 and 2026. We subsequently extracted the reproduced papers of each reproducibility study from the abstract and labelled the original papers according to our methodology. Some to-be-reproduced articles that were selected by the papers in our TMLR dataset were published outside of our scope of venues, such as ACL and SIGIR, although the vast majority (84.62%) appeared at the venues we included in our original study, such as AAAI, ICML, NeurIPS and ICLR. After labelling the original studies, we sought to apply the labels of Gundersen et al. [S6] to this new dataset, running into several limitations:

- Reproducibility studies submitted to TMLR have very high expectations in terms of effort by the independent investigators; more often than not, the papers have more than one author, and, as they are expected to extend upon the original study in addition reproducing it, it is not unreasonable to assume this exceeds the forty working hour limit of a single individual used by Gundersen et al. [S6].

- The increase of budget has implications on how we can apply our methodology to this extended dataset. Our work focuses on estimating the amount of unnecessary labour for independent reproduction due to lacking documentation; this means that a TMLR study finding that a claim is not reproducible more likely than not will indicate some methodological flaw in the original work than an 'inability' of the independent investigators to reproduce it due to lacking documentation. This entails, in the scope of our work, that the independent investigators in our sample will likely have 'paid the cost', even if it was unreasonably high.

- A smaller concern is that Gundersen et al. [S6] labelled reproduction attempt in terms of experiments reproduced, whereas TMLR publications usually divide up the elements of the reproduction in terms of claims made by the original authors.

Based on the reasons stated above, we adapted our original protocol towards the problem types Gundersen et al. [S6] encountered in their work. Besides labelling the claims aimed to be reproduced and the results thereof, we also inspected each study for issues encountered during the attempt at reproducing earlier work. As the scope of each TMLR reproduction study is larger than those considered by Gundersen et al. [S6], we extended the problem types with *ten* new labels, as shown in Table S1.

| **ID** | **Source** | **Description of Problem** | **Covered** |
|---|---|---|---|
| P21 | Code | Installation of the code was difficult to execute | Implementation |
| P22 | Data | The generator or simulator code was not provided or available | Data |
| P23 | Code | Code was not shared at all | Implementation |
| P24 | Contact | Contact with the authors was necessary and yielded more information | Yes |
| P25 | Contact | Contact with the authors was necessary but received no (useful) response | Yes |
| P26 | Code | A bug was detected in the code | No |
| P27 | Article | It is unclear how the hyperparameter value was selected | Configuration |
| P28 | Data | Part of the data requires a key | Data |
| P29 | Resources | The code is inefficient and requires optimisation | No |
| P30 | Resources | Running the experiments requires a substantial amount of compute | No |

Table S1: Extension of the problem classification by Gundersen et al. [S6]. The first column shows the new problem ID, the second column a source description as done in Gundersen et al. [S6], the third column the description of the problem, and the last column the dimension of our methodology covering the problem. Note that for P24 and P25, we simply show 'yes', as this covers various questions about lacking or ambiguous documentation. For the definition of the first twenty problem types, please see Gundersen et al. [S6] or our repository.

As shown in Table S1, our methodology covers seven out of ten new problems independent investigators encountered during their reproduction attempts. With regards to P29 and P30, these are mainly to allow for more detailed expressions of issues of compute than P20. P26, a bug was detected in the code, has also not been covered by our methodology, as these are usually only discovered at runtime rather than inspecting the code documentation for completeness and quality. With these new labels, we joined the two datasets resulting in a total of *61* reproduction attempts.

### S2.2.2 Analysis

In Figure S1, we quantify the occurrences of each problem type in the extended dataset, sorted by how it is covered by our methodology.

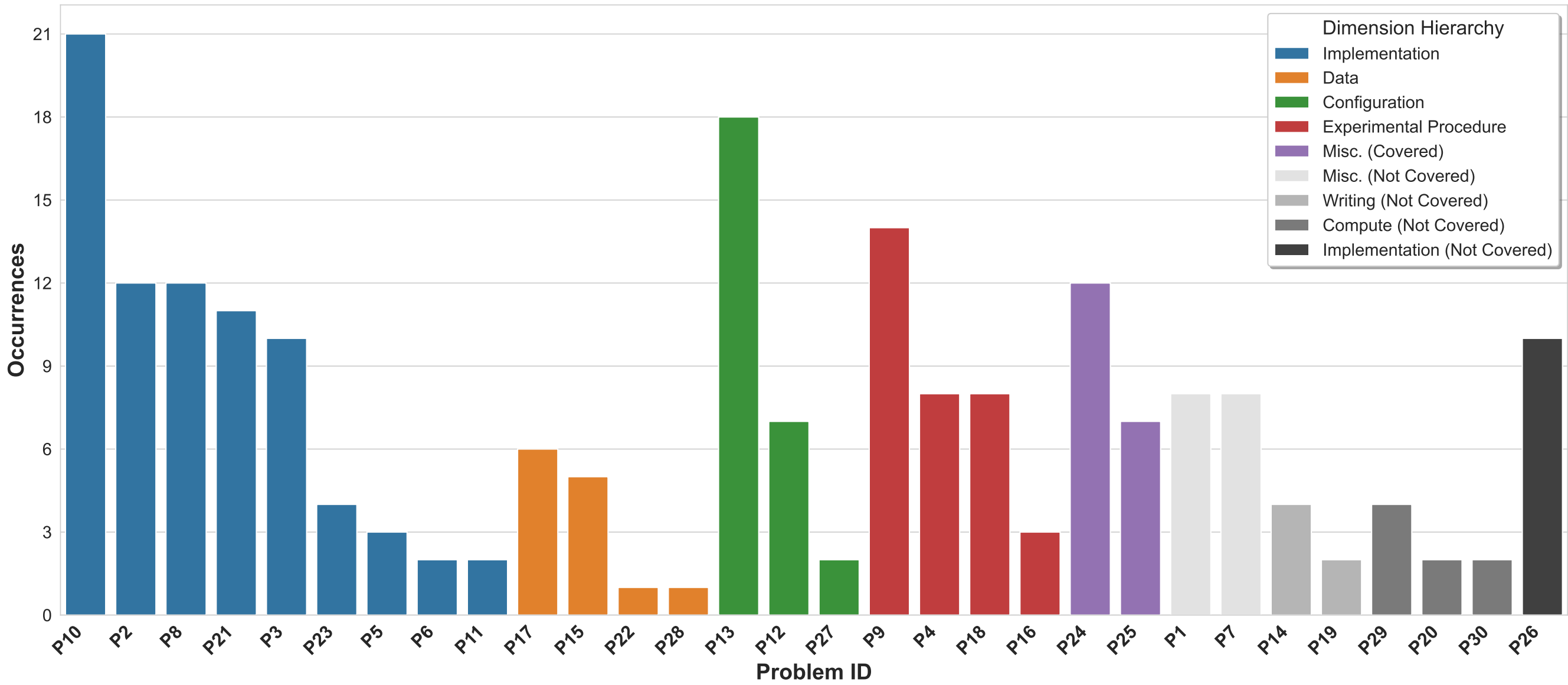


Figure S1: The amount of occurrences of each problem type in the reproducibility study dataset, sorted, left to right, based on their coverage by our methodology dimensions (Implementation, Data, Configuration, Experimental Procedure), followed by those that are not covered in grey and black. It is directly apparent that P10 (21), P13 (18), P9 (14) and P24 (12) are occurring most frequently. Overall, 80.86% of the reported problems are captured by our methodology.

Based on the findings in Figure S1, our method captures a vast majority of the encountered issues in practice. Those that are not represented in our methodology are largely explainable due to the fact that they have little or no overlap with the documentation responsibilities of the authors.

However, there some important details we would like to emphasise. Firstly, the most frequently encountered problems (P10, P13, P9 and P24) are captured strongly by our methodology and associated with substantial increases in reproducibility cost. Secondly, there are few issues encountered with the data dimension; only in two studies did independent investigators record such problems (P22, P28), although this was largely biased by the fact that Gundersen et al. [S6] considered only studies with public data. We found this to be the case for the TMLR studies as well: only two datasets used throughout 39 reproducibility studies were subject to (partial) data privacy, one of which was resolved after direct contact with the authors. The other data problem was an issue related to code provided by original authors using a data scraper with a privatised API requiring an API key (P28).

In our original analysis we found that the majority of published AI research relies on the usage, yet still found that 3.99% of AI publications rely on solely private data. Yet in our TMLR dataset we only find a singular case of an issue with (semi) private data, showing a possible bias of reproduction attempts towards studies with public data. This bias may suggest that a significant share of published research is not even attempted to be reproduced — an estimated 3.99% of all published research based on our data set. The overuse of public benchmark datasets may lead to over-fitting to specific instances of a given problem or task, which may have detrimental effects on performance beyond those limited use cases [S7] — possibly affecting the reproducibility of conclusions that presume the results to be generalisable or transferable to other instances or tasks.

Furthermore, we found in the 19 cases in the TMLR data where independent investigators sought

contact with original authors (P24, P25) — Gundersen et al. [S6] explicitly avoided contact with the authors: "We did not contact the authors as the documentation should contain all necessary information." In 63.16% of the reported cases, the original authors responded. This gives rise to a form of semi-public information; the authors clearly omitted some crucial information required by independent investigators, yet this was not included directly in the original publication. This elucidates the need for concise and comprehensive documentation — especially with known decay rates of the correctness of contact details found by Vines et al. [S8], showing that author contact is an unreliable method of information acquisition. This is unreliability of contacting authors is found in our TMLR dataset as well; over one third of the attempts at obtaining important information from original authors were unsuccessful.

Lastly, the TMLR data also highlights a possible limitation of our methodology; a frequently occurring problem in these reproducibility studies were related to the installation procedure of code provided by original authors (P21, eleven occurrences). This was captured by our protocol; a cost increase of one was attached if no instructions were provided at all on how to install the code (e.g. dependencies, packages, versioning). However, we found that in at least two studies this was provided, yet independent investigators still encountered this problem. Nonetheless, we believe this is still fairly represented in our methodology; the authors clearly made an effort to document the procedure, and they cannot be expected to ensure that it works across all conceivable target platforms, let alone stand the test of time with the speed at which software dependencies change and software is deprecated. Nonetheless, this issue illustrates that our approach opens the door to alternative cost schemes that may further refine how the problem is extracted. For example, a protocol based on empirical data could serve as a strong alternative foundation. However, as such data is extremely costly to create, we believe such an effort is a study on its own and thus leave this for future work.

Furthermore, the frequency of installation issues raises a new question: "How can we as a community improve the best practices when it comes to code installation documentation?" Our observation here is supported by the empirical study of Samuel and Mietchen [S9], who found that only 10 388 notebooks within their dataset of 22 578 Python notebooks successfully installed 'out of the box' using a requirements file, even though 15 817 notebooks had their dependencies declared in a standard file. Thus, in only 70.1% of the cases the installation is properly documented, but only in 65.6% of the 70.1% cases Samuel and Mietchen [S9] were able to execute the code. This means that Samuel and Mietchen [S9] found an effective installation rate of 46.0%, which demonstrates the frequency and scale of this problem.

Furthermore, the artifact decay rate does not only affect code but also data, and its subsequent impact on the cost of reproducibility, must not be underestimated. As reported by Vines et al. [S8], the availability of data artifacts from published studies declines by 17% per year post-publication.

## S2.3 Intra-annotator analysis

As discussed previously, a limitation of our study is the lack of reviewing resources. Although we validated our methodology by studying the agreement between various reviewers on the same publications, we have not yet investigated possible inconsistency of a single reviewer on this dataset — the within-person inconsistency or error [S5]. To measure this, we randomly sampled three previously annotated samples from each venue and year resulting in 63 publications. We excluded those previously labelled as *theoretical* from sampling, as we draw our conclusions from and aim our actionable findings at empirical research. Furthermore, we have omitted expertise from this experiment, as we have already concluded that our methodology was not sufficiently grounded to accurately capture this dimension.

In a preliminary analysis, we immediately detected a strong outlier; this was determined to be the result of our review on "Three rates of convergence or separation via U-statistics in a dependent

framework", published in JMLR in 2022. The root cause for this extreme intra-annotator variation (an increase of 7 in implementation) was determined to be a deleted GitHub repository, where the authors originally hosted their implementation. This is an example of data-drift rather than intra-annotator variation, and has therefore been excluded from our subsequent analysis. Nonetheless, this outlier serves as a clear-cut example of the dark pattern regarding 'dead' implementation URLs. We continued our analysis with a sample size of 62, representing 6.75% of our full dataset.

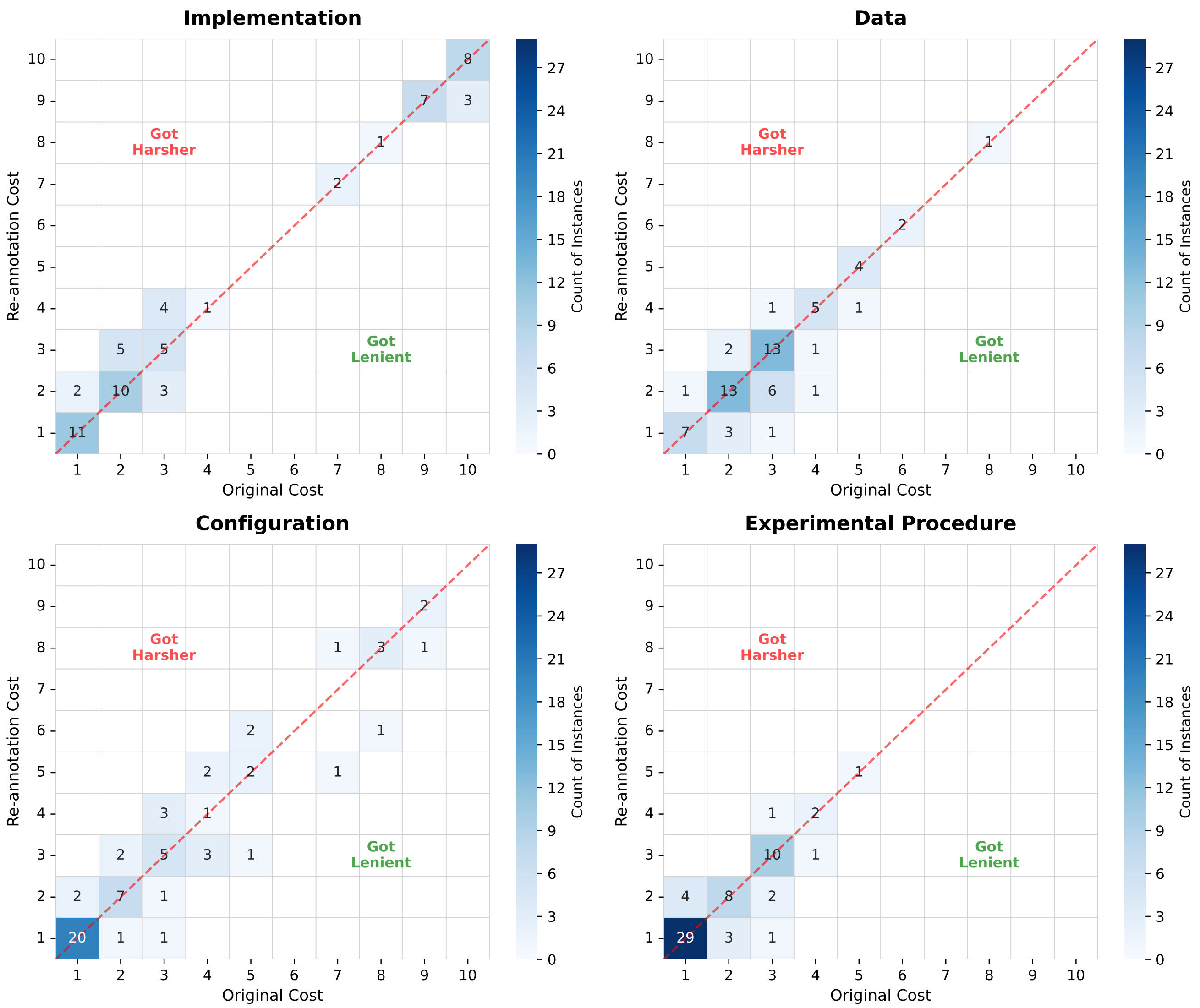


Figure S2: Confusion matrix of the original labels produced by the reviewer (x-axis) and a second review conducted by the same reviewer several months later (y-axis) per dimension. The colour intensity indicates the frequency of the occurrences, which are labelled per cell with their exact values. The diagonal line indicates exact overlap (agreement), whereas below the diagonal suggest labels have drifted to lower costs (i.e. the reviewer 'became lenient'), and above the diagonal suggest labels have drifted to high costs (i.e. the reviewer 'became harsher').

In Figure S2, we present the results of our investigation of within-reviewer inconsistency in the form of a confusion matrix. Overall, the scores have changed in a few cases, most frequently in the data and configuration categories, with inconsistencies up to two points, but mostly limited to one. They are also relatively spread out across the main diagonal indicating perfect consistency. We noticed one exception: studies that previously received a cost of three now frequently receive a cost of two. Based on our methodology, this is likely caused by the rule set regarding the descriptions of

data; cost may increase when the description of the dataset used or the statistics regarding the data are lacking. What constitutes 'sufficient' descriptions or statistics is relatively subjective, and this is reflected in the data presented here. Nonetheless, our analysis indicates overall low within-reviewer inconsistency.

| | MAD | ICC | 95% CI | P-Value | Label |
|---|---|---|---|---|---|
| Implementation | 0.27 | 0.989 | [0.98, 0.99] | 0.000 | Excellent |
| Data | 0.31 | 0.908 | [0.85, 0.94] | 0.000 | Excellent |
| Configuration | 0.42 | 0.953 | [0.92, 0.97] | 0.000 | Excellent |
| Experimental Procedure | 0.21 | 0.885 | [0.82, 0.93] | 0.000 | Good |

Table S2: The intraclass correlation coefficient (ICC) over the original and re-evaluation review with 95% confidence interval, P-Value and their interpreted labels based on Koo and Li [S10].

To statistically assess the within-reviewer inconsistency, we calculated the intraclass correlation coefficient, as seen previously in the main article. The results are shown in Table S2, calculated using the ICC-1 model, as in this scenario, we have a single rater assessing the same items twice. We find excellent agreements for implementation, data and configuration dimensions, and a good agreement for the experimental procedure. The P-values for each test are less than 0.0001, easily passing our threshold of 0.05 for statistical significance. To conclude, although some reviewer inconsistency has been detected, this effect is minor and thus does not significantly impact our analysis or conclusions.

## S2.4 Enriching the Data Set

To expand the data set and open up more angles of analysis, we leveraged SCOPUS — the curated abstract and citation database of Elsevier — through the tool from Rose and Kitchin [S11] to yield more meta-data on each evaluated paper. Although promising, we have gained little insights thus far using these extra features. However, we did find one interesting point, using the amount of citations per paper; when analysing the relationship between citation count and our dimensions of cost we found it difficult to determine any consistency between these variables. Thus, we determined the Kendall correlation coefficient between each dimension per year and the number of citations provided by SCOPUS. We calculated the correlation per year, as more recent papers have had less opportunity to receive citations than older papers. The results can be found in Table S3.

| # Citations | Implementation | Data | Configuration | Exp. Procedure |
|---|---|---|---|---|
| 2022 | **-0.10**(0.02) | -0.08(0.06) | **-0.10**(0.02) | 0.01(0.85) |
| 2023 | -0.06(0.15) | -0.03(0.53) | 0.02(0.60) | 0.02(0.64) |
| 2024 | -0.05(0.31) | -0.07(0.12) | **0.10**(0.02) | 0.05(0.29) |

Table S3: The Kendall Correlation between the number of citations and the dimensions per calendar year. A negative correlation implies that lower cost correlates with a higher amount of citations. The statistically significant results are marked in **bold**.

Three results in Table S3 have been found to be statistically significant, however the correlation across the board are quite weak with the largest value being 0.10. Thus, deducing any relation between these variables based on this data is very weakly supported. Hence, we find in this exploration no signals that *better documented* studies receive more citations. As citations are a key metric to measure the success of a publication, this illustrates that, although the community finds high reproducibility standards important, applying best practices with regards to documentation has little effect on how it is received on the short (3 year) term. However, there is a caveat to this; we

have only collected data for the past three year and can see indications of an up tick of correlation year-on-year in for example implementation and configuration. Hence, it may be hypothesised that, although high quality documentation may be unrewarding on the short term, on the long term it will survive the test of time. We leave such investigations for future work.

# S3. Extended Background

In this work, we use the term 'reproducibility' to refer to a general scientific concept, rather than limiting it to the field of AI or computer science, for which we find two possible standards: Goodman et al. [S12] and Gundersen [S13], who both used quite similar definitions and terminology. Goodman et al. [S12] separated the definitions of reproducibility into three types: methods, results and inferential reproducibility, which partially overlap with the definitions in Gundersen [S13], who defined three degrees of reproducibility: outcome, analysis and interpretation reproducible. Both Goodman et al. [S12] and Gundersen [S13] presented their types of reproducibility within a hierarchical ordering. Goodman et al. [S12] referred to methods reproducibility as the ability to repeat the procedure of the study on the level of implementation, e.g. "methodologically reproducible", where the experimental procedure can be repeated using the documentation provided. This does not include the outcome or the conclusions that are drawn from the experiment. We note a discrepancy between fields: in psychology, methodologically reproducible would mainly refer to reproducing an experimental set-up, whereas in computer science, this would also refer to the reproducibility of the implementation or execution, e.g. by running code. Although the other categories and degrees, such as results reproducibility and outcome reproducibility or inferential and interpretation reproducibility, overlap more between these two approaches, for conciseness and clarity, we refrain from using definitions of categories and degrees. We find, however, the definitions by Goodman et al. [S12] to be more ambiguous, as they lack a definition of independent investigators ('reproducers'), as well as the concept of what constitutes the documentation of a method.

We also disagree with the reasoning that the search for 'truth' is the main motive for reproducibility, and argue that in the field of AI, it is rather the credibility of the method [S14]. This discrepancy between definitions likely originates in the epistemological nature of both fields; the framing by Goodman et al. [S12] reflects an approach that more closely models the discovery of knowledge in the natural world — in AI, we rather consider the discovery of knowledge, at least to some degree, to occur by determining and comparing the performance of some methodologies for solving a given problem or accomplishing a given task. Hence, by reproducing such findings, we add credibility to specific methodologies.

Crook et al. [S15] discussed various approaches regarding reproducibility in computational neuroscience. They defined reproduction and replication of an experiment: An independent investigator either reproduces an experiment, or replicates it 'using the same code', and they consider the latter to be 'certainly easier than independent reproduction'. Later, they redefined these terms as internal and external replicability, and introduced the concept of cross-replicability: The ability to simulate the same model with different software. Lastly, they defined reproducibility as the ability to implement a model independently, without using the source code provided by the original authors.

We refrain from using these definitions, due to their ambiguity and limitations. The definition of reproducibility excludes the source code as part of the documentation, which we find an unnecessary and somewhat unnatural constraint. The terms of internal and external replicability are redundant from our point of view: Reproducibility should be measured based on the documentation made publicly available by original authors, and should thus not vary based on the relation to the independent investigator; one could even argue that a closer relation could create a conflict of interest. The information provided to a reader for 'external replicability' should be the same as for reproducibility. Lastly, the term cross-replicability refers to the ability to re-implement a method

outside the domain of the source code. We see this as a constrained version of the re-implementation of methods within reproducibility.

## S3.1 Alternative costs

Alternatively to documentation quality, other types of reproducibility cost can be conceived. For example, one could focus on resource requirements in terms of monetary cost. A famously expensive example from physics involves CERN's Large Hadron Collider [S16], a ten-year project with a 4.75 billion USD price tag [S17]. Reproducing research that requires such an instrument would be unrealistic, unless it is made available to independent investigators, and may also imply other costs, in terms of effort (for securing access) or time (the resource is in high demand). The issue of resource requirements is becoming more prevalent in AI research, e.g., in the context of large deep learning models. Large language models (LLMs), such as Open AI's ChatGPT-4 and Microsoft's Copilot models, take several months to train on thousands of state-of-the-art GPUs. Tian et al. [S18] conducted a reproducibility study of AlphaZero [S19] and found that "A single training run requires [..] days of training on thousands of TPUs, which is an unattainable level of compute for the majority of the research community". The authors further state: "When combined with the unavailability of code and models, the result is that the approach is very difficult, if not impossible, to reproduce, study, improve upon, and extend". High demand for compute can be accepted as an "irreducible cost" by the original authors, as it is in general not in their reach nor their responsibility to make these resources available, whereas lack of documentation is clearly a "reducible cost" that could be mitigated relatively easily. Yet, as we have seen in subsection S2.2 and Figure S1 specifically, the high demand for computing resources is a frequently reported problem in reproducibility studies. Hence, the issue of compute with substantive implications for reproducibility which we leave for future work.

# S4. Extended Quantification Studies

In Raff [S2], the author defines features to test for correlation with reproducibility of studies. The most directly linked feature to our work is the specification of hyperparameters, which we evaluate under configuration cost, and pseudo code, which we evaluate under implementation cost. It is important to note that both of these features are considered in Raff [S2] as 'mildly subjective', which also finds its place in our work as we enable our reviews to diverge from the set guidelines. One significant feature that we do not include in our work is required compute. We have chosen to exclude this, as our focus lies on the documentation provided by authors rather than the accessibility of compute. Another reason, unrelated to the work of Raff [S2], is that the variation that can be caused by different low-level systems (such as a CPU/GPU architecture or drivers) is more closely aligned with *outcome* reproducible rather than *interpretation* reproducible, e.g. being able to draw the same conclusion. Variations in (low-level) system software may impact the results outcome in unexpected ways, even if factors of stochasticity are well documented; for example seeds for non-deterministic algorithms. However, exactly reproducing the outcomes is a highly strict view on reproducibility which often may be unnecessary; as long as outcomes support the same interpretations for the hypothesis (for example, varying outcomes but statistical tests still prove the same significances) we find this sufficient to determine a study reproducible.

Due to its widespread applicability and popularity, the impact of irreproducible machine-learning research is not limited to its field [S20–S22]. In Kapoor and Narayanan [S23], the authors study 642 publications across 17 scientific fields, ranging from medicine, satellite imaging and cybersecurity to the political sciences. They identified 294 cases of data leakage and classified them into eight categories. These classifications cover situations such as ill-separated training and test sets, or the

lack thereof entirely, as well as more complicated examples such as temporal leakage or feature selection and dataset pre-processing on the test set. The authors conclude that the application of ML methods has many pitfalls and that each is rediscovered independently by each community. To prevent or mitigate these issues, Kapoor et al. [S24] provides recommendations for a stronger review process regarding reproducibility documentation, which can help to alert the authors to their mistakes, thus contributing to more realistic or well-founded outcomes. The authors conclude that this identification process of these pitfalls is a first step in the direction of addressing this interdisciplinary crisis. Secondly, increasing the focus on documentation on the application of AI methods regarding reproducibility emphasises the importance to the community.

# S5. Extended figures and tables

In this section, we present several graphs and tables that are used as a supporting role for our main results. In Table S4 we present the median, quantiles and data skew. In Table S5 we represent the results of the normality test. In Table S6 we show the permutation test between all sources per dimension, testing which sources are significantly less costly than others. In Table S7 we present the results of the Kolmogorov-Smirnov test and permutation test for award winning papers. In Figure S3 we present the correlation per source and overall.

| | **Implementation** | | | | | **Data** | | | | |
|---|---|---|---|---|---|---|---|---|---|---|
| | Q1 | Median | Q3 | IQR | Skew | Q1 | Median | Q3 | IQR | Skew |
| AAAI | 2 | 6.50 | 9 | 7 | -0.09 | 1 | 2.00 | 3 | 2 | 1.35 |
| IJCAI | 2 | 5.00 | 9 | 7 | 0.14 | 1 | 2.00 | 4 | 3 | 1.47 |
| ICLR | 2 | 2.00 | 8 | 6 | 0.86 | 2 | 3.00 | 4 | 2 | 1.31 |
| ICML | 1 | 2.00 | 8 | 7 | 0.72 | 1 | 3.00 | 3 | 2 | 0.80 |
| NeurIPS | 2 | 2.00 | 5 | 3 | 1.16 | 2 | 3.00 | 4 | 2 | 0.92 |
| JAIR | 2 | 3.00 | 9 | 7 | 0.32 | 2 | 3.00 | 4 | 2 | 0.87 |
| JMLR | 2 | 3.00 | 10 | 8 | 0.17 | 2 | 3.00 | 4 | 2 | 0.46 |

| | **Configuration** | | | | | **Experimental Procedure** | | | | |
|---|---|---|---|---|---|---|---|---|---|---|
| | Q1 | Median | Q3 | IQR | Skew | Q1 | Median | Q3 | IQR | Skew |
| AAAI | 2 | 3.50 | 5 | 3 | 0.95 | 1 | 2.00 | 3 | 2 | 1.41 |
| IJCAI | 2 | 3.00 | 5 | 3 | 1.07 | 1 | 2.00 | 3 | 2 | 1.57 |
| ICLR | 2 | 3.00 | 4 | 2 | 1.64 | 1 | 2.00 | 3 | 2 | 1.34 |
| ICML | 1 | 3.00 | 3 | 2 | 1.73 | 1 | 1.00 | 3 | 2 | 1.05 |
| NeurIPS | 3 | 3.00 | 4 | 1 | 1.36 | 1 | 2.00 | 3 | 2 | 0.85 |
| JAIR | 1 | 2.50 | 4 | 3 | 1.35 | 1 | 1.00 | 2 | 1 | 1.68 |
| JMLR | 1 | 3.00 | 4 | 3 | 1.18 | 1 | 1.00 | 2 | 1 | 1.80 |

Table S4: The median, first and third quantile and interquantile range of each dimension per venue. The skewness of the distribution is nearly always positive, with the exception of AAAI in the implementation dimension, thus motivating the representation of median rather than mean as it is frequently substantially affected by right skewed distribution outliers except for the implementation dimension which has relatively mild skew values.

| | Implementation | Data | Configuration | Experimental Procedure |
|---|---|---|---|---|
| AAAI | 1728.65 (0.00) | 35.00 (0.00) | 17.19 (0.00) | 36.18 (0.00) |
| IJCAI | 1398.58 (0.00) | 36.10 (0.00) | 18.00 (0.00) | 43.33 (0.00) |
| ICLR | 29.38 (0.00) | 37.34 (0.00) | 51.78 (0.00) | 34.83 (0.00) |
| ICML | 72.84 (0.00) | 12.35 (0.00) | 52.38 (0.00) | 18.42 (0.00) |
| NeurIPS | 20.97 (0.00) | 22.03 (0.00) | 34.03 (0.00) | 12.82 (0.00) |
| JAIR | 3948.80 (0.00) | 10.70 (0.00) | 23.84 (0.00) | 39.46 (0.00) |
| JMLR | 1075.33 (0.00) | 8.91 (0.01) | 21.25 (0.00) | 48.88 (0.00) |

Table S5: Normality test [S25] of all venues and dimensions, where the p-values are shown in brackets. The results are statistically significant for each venue. Thus, we find evidence for each venue that the results are not normally distributed.

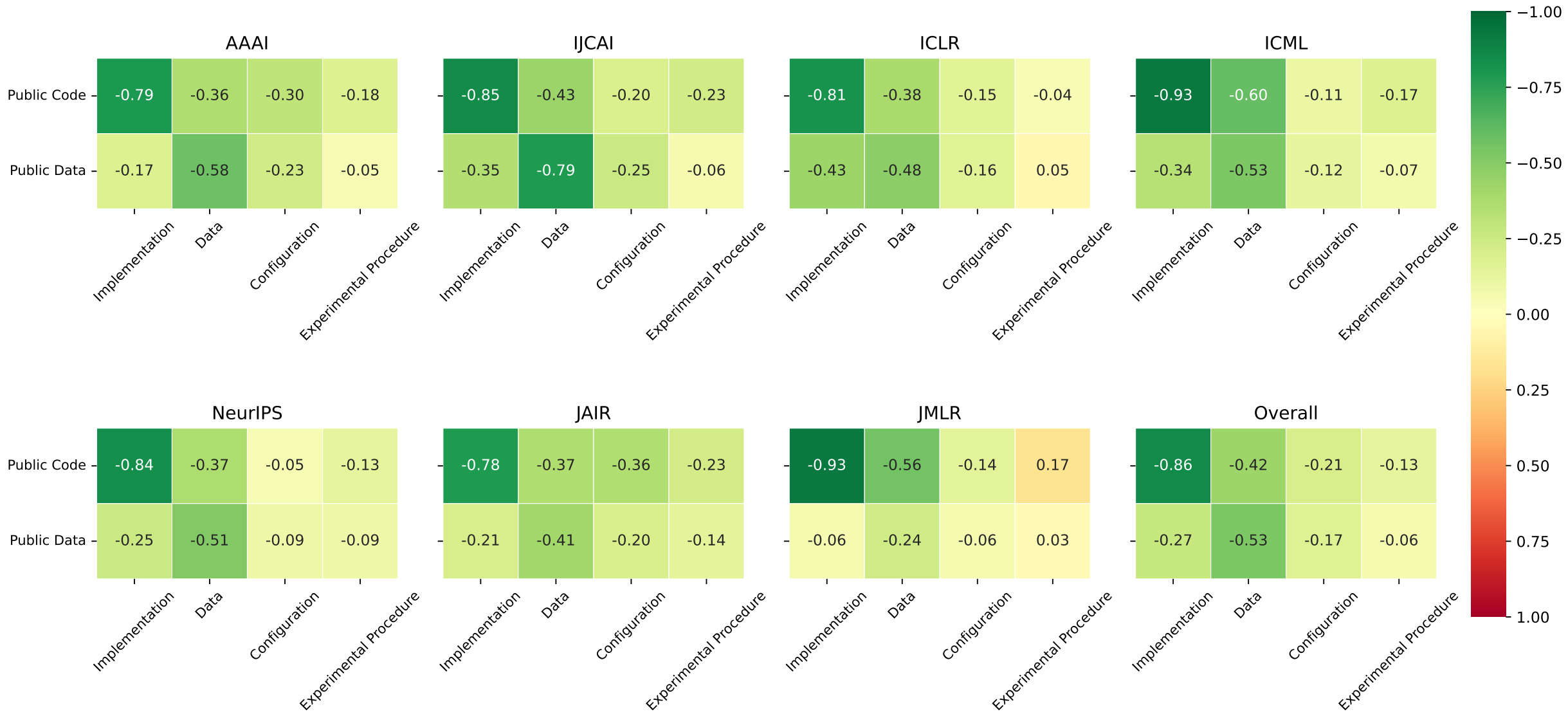


Figure S3: Kendall Correlation heat map of the dimensions and the collected metrics, namely implementation link (URL) and proportion of public data sets (Public Data). A negative correlation indicates lower cost.

# S6. Data Collection

In the sampling process, we manually excluded the following categories of papers from each source, as they generally lack a full empirical evaluation:

- In AAAI we exempted the (student) abstracts
- In IJCAI we exempted the survey, doctoral consortium, and early career tracks. The Special Track on AI for Good (Projects) and 'demonstration track' were curated for empirical evaluations. 'Extended abstract' papers were included when the full version was linked.
- In ICLR, ICML and NeurIPS we exempted papers originating from a journal.
- In ICML we curated Position Papers for an empirical evaluation.

In our data scraping, we supplemented the data from OpenReview manually to ensure representative documentation from the point of view of a reviewer for each paper. We have two specific

notes: JMLR 2024 only had 49 accepted papers at the time of sampling, thus all were used. In JAIR 2022 "Marginal Distance and Hilbert-Schmidt Covariances-Based Independence Tests for Multivariate Functional Data" where code is available upon request. We requested, received, and reviewed accordingly. Regarding the reviews of papers with awards, we included from each source/year the papers with 'best paper award' up to 8 papers. If more were presented with such an award 8 were sampled at random due to resource and time constraints and to avoid data undersampling of the 'regular' paper population.

During sampling, the following papers evaluated for possible conflict of interest:

- IJCAI 2023 we rejected "Solving the Identifying Code Set Problem with Grouped Independent Support" due to the first author being a former PhD candidate of one of the authors
- IJCAI 2023 we still evaluated "One Model, Any CSP: Graph Neural Networks as Fast Global Search Heuristics for Constraint Satisfaction" as although the researchers come from the same institution we have no direct contact with them
- ICML 2023 we rejected "Expectation-Complete Graph Representations with Homomorphisms" due to a personal connection of the first author to the authors.
- ICML 2022 we still evaluated "Markov Chain Monte Carlo for Continuous-Time Switching Dynamical Systems" even though the code is hosted RWTH Aachen (No direct affiliation, authors are from TU Darmstadt)
- NeurIPS 2022 we rejected "JAHS-Bench-201: A Foundation For Research On Joint Architecture And Hyperparameter Search" as HH has publications with Frank Hutter
- JMLR 2024 we rejected "Critically Assessing the State of the Art in Neural Network Verification" as one of the authors also authored this paper
- JMLR 2022 we rejected "SMAC3: A Versatile Bayesian Optimization Package for Hyperparameter Optimization" due to personal connections with the authors.
- JAIR 2024 we rejected "A Hybrid Intelligence Method for Argument Mining" due to a personal connection to the authors
- JAIR 2024 we rejected "Improving Reproducibility in AI Research: Four Mechanisms Adopted by JAIR" as it was authored by the authors
- JAIR 2023 we rejected "MDP Playground: An Analysis and Debug Testbed for Reinforcement Learning" due to a personal connection with one of the authors
- JAIR 2022 we rejected "FOND Planning with Explicit Fairness Assumptions" due to a connection with the third author.

**Implementation**

| | AAAI | IJCAI | ICLR | ICML | NeurIPS | JAIR | JMLR |
|---|---|---|---|---|---|---|---|
| AAAI | - | 0.81 | 1.00 | 1.00 | 1.00 | 0.90 | 0.77 |
| IJCAI | 0.20 | - | 1.00 | 0.99 | 1.00 | 0.67 | 0.46 |
| ICLR | **0.00** (-1.66) | **0.00** (-1.27) | - | 0.34 | 0.62 | **0.01** (-1.06) | **0.00** (-1.33) |
| ICML | **0.00** (-1.47) | **0.01** (-1.08) | 0.67 | - | 0.77 | **0.03** (-0.88) | **0.01** (-1.14) |
| NeurIPS | **0.00** (-1.77) | **0.00** (-1.38) | 0.40 | 0.25 | - | **0.01** (-1.18) | **0.00** (-1.44) |
| JAIR | 0.10 | 0.34 | 0.99 | 0.97 | 1.00 | - | 0.31 |
| JMLR | 0.24 | 0.55 | 1.00 | 0.99 | 1.00 | 0.70 | - |

**Data**

| | AAAI | IJCAI | ICLR | ICML | NeurIPS | JAIR | JMLR |
|---|---|---|---|---|---|---|---|
| AAAI | - | 0.14 | 0.10 | 0.28 | **0.02** (-0.42) | 0.15 | **0.00** (-0.67) |
| IJCAI | 0.87 | - | 0.49 | 0.74 | 0.25 | 0.55 | **0.05** (-0.42) |
| ICLR | 0.91 | 0.54 | - | 0.79 | 0.24 | 0.58 | **0.03** (-0.40) |
| ICML | 0.74 | 0.29 | 0.24 | - | 0.06 | 0.30 | **0.00** (-0.55) |
| NeurIPS | 0.98 | 0.77 | 0.78 | 0.95 | - | 0.82 | 0.12 |
| JAIR | 0.87 | 0.48 | 0.45 | 0.73 | 0.20 | - | **0.03** (-0.44) |
| JMLR | 1.00 | 0.96 | 0.97 | 1.00 | 0.89 | 0.98 | - |

**Configuration**

| | AAAI | IJCAI | ICLR | ICML | NeurIPS | JAIR | JMLR |
|---|---|---|---|---|---|---|---|
| AAAI | - | 0.79 | 1.00 | 1.00 | 0.95 | 1.00 | 0.96 |
| IJCAI | 0.22 | - | 1.00 | 1.00 | 0.76 | 0.95 | 0.83 |
| ICLR | **0.00** (-1.08) | **0.00** (-0.84) | - | 0.55 | **0.00** (-0.64) | 0.20 | **0.03** (-0.53) |
| ICML | **0.00** (-1.10) | **0.00** (-0.86) | 0.48 | - | **0.01** (-0.66) | 0.19 | **0.04** (-0.55) |
| NeurIPS | 0.06 | 0.26 | 1.00 | 1.00 | - | 0.89 | 0.65 |
| JAIR | **0.01** (-0.83) | 0.05 | 0.82 | 0.82 | 0.12 | - | 0.23 |
| JMLR | **0.04** (-0.55) | 0.19 | 0.97 | 0.97 | 0.37 | 0.78 | - |

**Experimental Procedure**

| | AAAI | IJCAI | ICLR | ICML | NeurIPS | JAIR | JMLR |
|---|---|---|---|---|---|---|---|
| AAAI | - | 0.83 | 0.97 | 1.00 | 0.98 | 1.00 | 1.00 |
| IJCAI | 0.20 | - | 0.83 | 0.99 | 0.86 | 1.00 | 1.00 |
| ICLR | **0.04** (-0.30) | 0.20 | - | 0.91 | 0.56 | 0.98 | 0.99 |
| ICML | **0.00** (-0.48) | **0.02** (-0.32) | 0.11 | - | 0.11 | 0.81 | 0.92 |
| NeurIPS | **0.03** (-0.32) | 0.17 | 0.49 | 0.91 | - | 0.98 | 1.00 |
| JAIR | **0.00** (-0.58) | **0.01** (-0.43) | **0.03** (-0.28) | 0.23 | **0.03** (-0.27) | - | 0.70 |
| JMLR | **0.00** (-0.65) | **0.00** (-0.49) | **0.01** (-0.34) | 0.11 | **0.01** (-0.33) | 0.35 | - |

Table S6: Permutation test of 1 000 000 resamples between sources per dimension, where each row is tested to be smaller than each column. The table presents the found $p$-values per comparison/test. The statistically significant results are highlighted in **bold** with the statistic values (distances) in brackets.

| **Implementation** | | |
|---|---|---|
| | KS Test | P-Test |
| AAAI | 0.20 (0.99) | 0.42 (0.63) |
| IJCAI | 0.35 (0.31) | 2.28 (0.95) |
| ICLR | 0.14 (0.94) | -0.06 (0.48) |
| ICML | 0.18 (0.51) | -1.09 (0.11) |
| NeurIPS | 0.15 (0.81) | -0.41 (0.31) |

| **Data** | | |
|---|---|---|
| | KS Test | P-Test |
| AAAI | 0.13 (1.00) | 0.07 (0.54) |
| IJCAI | 0.14 (0.99) | 0.39 (0.70) |
| ICLR | 0.14 (0.93) | -0.24 (0.33) |
| ICML | **0.33** (0.03) | **-1.12** (0.00) |
| NeurIPS | 0.22 (0.34) | -0.65 (0.06) |

| **Configuration** | | |
|---|---|---|
| | KS Test | P-Test |
| AAAI | 0.28 (0.86) | -0.79 (0.28) |
| IJCAI | **0.64** (0.00) | 2.43 (1.00) |
| ICLR | 0.13 (0.96) | -0.34 (0.26) |
| ICML | 0.17 (0.64) | -0.77 (0.08) |
| NeurIPS | 0.20 (0.44) | 0.99 (0.99) |

| **Exp. Proc.** | | |
|---|---|---|
| | KS Test | P-Test |
| AAAI | 0.43 (0.36) | -0.53 (0.27) |
| IJCAI | 0.26 (0.68) | 0.64 (0.94) |
| ICLR | 0.14 (0.94) | -0.22 (0.27) |
| ICML | 0.10 (0.98) | -0.25 (0.18) |
| NeurIPS | 0.10 (0.99) | -0.25 (0.20) |

Table S7: Kolmogorov–Smirnov test [S26] and Permutation test per source and dimension between papers with and without awards. The P-values are in brackets. The statistically significant results are highlighted in **bold**.

# References


S1. Gundersen, O.E., and Kjensmo, S. (2018). State of the art: Reproducibility in artificial intelligence. In Proceedings of the 32nd AAAI Conference on Artificial Intelligence vol. 1. pp. 1644–1651. doi: https://doi.org/10.1609/aaai.v32i1.11503.

S2. Raff, E. (2019). A step toward quantifying independently reproducible machine learning research. In Proceedings of the 33rd International Conference on Neural Information Processing Systems vol. 32. pp. 5485–5495.

S3. LeCun, Y., Bottou, L., Bengio, Y., and Haffner, P. (1998). Gradient-based learning applied to document recognition. Proceedings of the IEEE *86*, 2278–2324. doi: https://doi.org/10.1109/5.726791.

S4. Krizhevsky, A., and Hinton, G. (2009). Learning multiple layers of features from tiny images. Computer Science Department, University of Toronto. URL: http://www.cs.utoronto.ca/~kriz/learning-features-2009-TR.pdf Accessed: 01-07-2026.

S5. Bayerl, P.S., and Paul, K.I. (2011). What determines inter-coder agreement in manual annotations? a meta-analytic investigation. J. Comput. Linguist. *37*, 699–725. doi: https://doi.org/10.1162/COLI_a_00074.

S6. Gundersen, O.E., Cappelen, O., Mølnå, M., and Nilsen, N.G. (2025). The unreasonable effectiveness of open science in ai: A replication study. Proceedings of the 39th AAAI Conference on Artificial Intelligence *39*, 26211–26219. doi: https://doi.org/10.1609/aaai.v39i25.34818.

S7. Sourget, T., Akkoç, A., Winther, S., Galsgaard, C.L., Jiménez-Sánchez, A., Juodelyte, D., Petitjean, C., and Cheplygina, V. (2024). [citation needed] data usage and citation practices in medical imaging conferences. In N. Burgos, C. Petitjean, M. Vakalopoulou, S. Christodoulidis, P. Coupe, H. Delingette, C. Lartizien, and D. Mateus, eds. Proceedings of the 7th International Conference on Medical Imaging with Deep Learning vol. 250 of *Proceedings of Machine Learning Research*. pp. 1475–1496.

S8. Vines, T.H., Albert, A.Y., Andrew, R.L., Débarre, F., Bock, D.G., Franklin, M.T., Gilbert, K.J., Moore, J.S., Renaut, S., and Rennison, D.J. (2014). The availability of research data declines rapidly with article age. Curr. Biol. *24*, 94–97. doi: https://doi.org/10.1016/j.cub.2013.11.014.

S9. Samuel, S., and Mietchen, D. (2024). Computational reproducibility of jupyter notebooks from biomedical publications. GigaScience *13*, 1–23. doi: https://doi.org/10.1093/gigascience/giad113.

S10. Koo, T.K., and Li, M.Y. (2016). A guideline of selecting and reporting intraclass correlation coefficients for reliability research. J. Chiropr. Med. *15*, 155–163. doi: https://doi.org/10.1016/j.jcm.2016.02.012.

S11. Rose, M.E., and Kitchin, J.R. (2019). pybliometrics: Scriptable bibliometrics using a python interface to scopus. SoftwareX *10*, 1–6. doi: https://doi.org/10.1016/j.softx.2019.100263.

S12. Goodman, S.N., Fanelli, D., and Ioannidis, J.P. (2016). What does research reproducibility mean? Sci. Transl. Med. *8*, 341ps12–341ps12. doi: https://doi.org/10.1126/scitranslmed.aaf5027.

S13. Gundersen, O.E. (2021). The fundamental principles of reproducibility. Philos. Trans. R. Soc. *379*, 1–15. doi: https://doi.org/10.1098/rsta.2020.0210.

S14. Popper, K. (1934). The logic of scientific discovery. Julius Springer, Hutchinson & Co.

S15. Crook, S.M., Davison, A.P., and Plesser, H.E. (2013). Learning from the past: Approaches for reproducibility in computational neuroscience. In 20 Years of Computational Neuroscience vol. 9 of *Springer Series in Computational Neuroscience* pp. 73–102.. Springer pp. 73–102. doi: https://doi.org/10.1007/978-1-4614-1424-7_4.

S16. Brüning, O., Burkhardt, H., and Myers, S. (2012). The large hadron collider. Prog. Part. Nucl. Phys. *67*, 705–734. doi: https://doi.org/10.1016/j.ppnp.2012.03.001.

S17. Knapp, A. (2012). How much does it cost to find a higgs boson? Forbes. Forbes Inc. URL: https://www.forbes.com/sites/alexknapp/2012/07/05/how-much-does-it-cost-to-find-a-higgs-boson/#1891ccaa3948.

S18. Tian, Y., Ma, J., Gong, Q., Sengupta, S., Chen, Z., Pinkerton, J., and Zitnick, L. (2019). ELF OpenGo: an analysis and open reimplementation of AlphaZero. In K. Chaudhuri, and R. Salakhutdinov, eds. Proceedings of the 36th International Conference on Machine Learning vol. 97 of *Proceedings of Machine Learning Research*. pp. 6244–6253.

S19. Silver, D., Hubert, T., Schrittwieser, J., Antonoglou, I., Lai, M., Guez, A., Lanctot, M., Sifre, L., Kumaran, D., Graepel, T., Lillicrap, T., Simonyan, K., and Hassabis, D. (2018). A general reinforcement learning algorithm that masters chess, shogi, and go through self-play. Science *362*, 1140–1144. doi: https://doi.org/10.1126/science.aar6404.

S20. Ball, P. (2023). Is AI leading to a reproducibility crisis in science? Nature *624*, 22–25. doi: https://doi.org/10.1038/d41586-023-03817-6.

S21. Gibney, E. (2022). Could machine learning fuel a reproducibility crisis in science? Nature *608*, 250–251. doi: https://doi.org/10.1038/d41586-022-02035-w.

S22. La Malfa, E., Petrov, A., Frieder, S., Weinhuber, C., Burnell, R., Nazar, R., Cohn, A., Shadbolt, N., and Wooldridge, M. (2024). Language-models-as-a-service: Overview of a new paradigm and its challenges. J. Artif. Intell. Res. *80*, 1497–1523. doi: https://doi.org/10.1613/jair.1.15865.

S23. Kapoor, S., and Narayanan, A. (2023). Leakage and the reproducibility crisis in machine-learning-based science. Patterns *4*, 1–12. doi: https://doi.org/10.1016/j.patter.2023.100804.

S24. Kapoor, S., Cantrell, E.M., Peng, K., Pham, T.H., Bail, C.A., Gundersen, O.E., Hofman, J.M., Hullman, J., Lones, M.A., Malik, M.M. et al. (2024). Reforms: Consensus-based recommendations for machine-learning-based science. Sci. Adv. *10*, eadk3452. doi: https://doi.org/10.1126/sciadv.adk3452.

S25. D'agostino, R., and Pearson, E.S. (1973). Tests for departure from normality. Biometrika *60*, 613–622. doi: https://doi.org/10.2307/2335012.

S26. Dodge, Y. (2008). The Concise Encyclopedia of Statistics. Springer.
