# Supplementary material for: The cost of reproducibility in artificial intelligence
Source: Patterns (N Y). 2026 Aug 27;7(10):101640. doi: 10.1016/j.patter.2026.101640 (PMC13674308; doi:10.1016/j.patter.2026.101640)
Supplement: Supplementary file 2 [file mmc2.pdf]

Article

Patterns

The cost of reproducibility in artificial intelligence

Highlights

- We analyze 918 AI publications between 2022 and 2024 for their reproducibility
- Papers published at ML conferences are overall easiest to reproduce
- Award-winning papers are not easier to reproduce than "regular" papers
- We find a dark pattern of 6.72% of URLs to code repositories being broken

Authors

Thijs Snelleman, Holger Hoos, Odd Erik Gundersen

Correspondence

snelleman@aim.rwth-aachen.de

In brief

Reproducibility is widely considered a cornerstone of the scientific method and thus a key element of research, yet researchers often experience a wide variety of issues when attempting to reproduce empirical studies in AI. This work explores the relationship between the reproducibility of publications and the standards of their corresponding venues.

Snelleman et al., 2026, Patterns *7*, 101640
October 9, 2026 © 2026 The Author(s). Published by Elsevier Inc.
https://doi.org/10.1016/j.patter.2026.101640

CellPress

Patterns

CellPress
OPEN ACCESS

Article

The cost of reproducibility in artificial intelligence

Thijs Snelleman,[1,4,5,*] Holger Hoos,[1,2] and Odd Erik Gundersen[3]
[1]Chair for AI Methodology, Faculty of Computer Science, RWTH Aachen University, 52062 Aachen, North Rhine-Westphalia, Germany
[2]Leiden Institute of Advanced Computer Science, Faculty of Science, Leiden University, 2333 CC, Leiden, South Holland, the Netherlands
[3]Department of Computer Science, Norwegian University of Science and Technology, 7034 Trondheim, Trøndelag, Norway
[4]Present address: Theaterstrasse 35–39, 52062, Aachen, Germany
[5]Lead contact
*Correspondence: snelleman@aim.rwth-aachen.de
https://doi.org/10.1016/j.patter.2026.101640

THE BIGGER PICTURE The reproducibility crisis is affecting empirical research across many fields of science, including artificial intelligence. As reproducibility is widely considered to be a cornerstone of the scientific method, it is of paramount importance that this crisis is mitigated if research is to progress as efficiently as possible. The quality of the documentation supplied by authors of scientific publications plays a crucial role in the reproducibility of their work. In this article, we consider this issue from a different angle; we consider the lack of documentation elements as an issue that increases the *cost* of reproducibility—i.e., the amount of (time) resources required for successfully reproducing a published scientific publication.

SUMMARY

Insufficiently documented publications can make independent reproduction an unnecessarily laborious task. We define the cost of reproducibility as the resources required to reproduce the findings of a study and we propose five dimensions of cost, using objective characteristics such as code and data. We review 918 publications between 2022 and 2024 at top AI venues to evaluate the variation in reproducibility costs, with the hypothesis that publications at venues with stricter standards are less costly. Papers published at machine learning conferences are less costly to reproduce than publication from other venues, 12.91% less costly compared with journals, and 16.52% less costly compared with general AI conferences. Award-winning papers, however, are not less costly to reproduce than regular papers, and 6.72% of implementation repository links were broken. These findings suggest that reproducibility costs can be significantly reduced through compliance-based mechanisms—provided they receive community support. We recommend that venues require the inclusion of reproducibility checklists in their publications, permit appendices for comprehensive documentation, and adopt low reproducibility cost as a key criterion for awards.

INTRODUCTION

For over a decade, across all fields of science, a crisis in reproducibility of research has been a persistent problem,[1] ranging from social sciences[2] to physics,[3] chemistry,[4] biology,[5] medicine,[6] and other fields including artificial intelligence (AI).[7–10] Recently, Gundersen et al.[11] attempted to reproduce the 30 most cited AI studies and found an average reproducibility rate of 50.0%, whereas Raff[12] estimated the reproducibility rate for machine learning (ML) studies to be 63.5%. This is shockingly low, yet not an unsolvable problem. In the social sciences, a crisis of reproducibility was assessed in 2009 by Schmidt,[2] reporting that reproducibility is "of major importance and highly respected," yet rarely discussed. In 2023, Korbmacher et al.[13] determined that replication improved in the social sciences through positive structural, procedural, and community-driven changes.

Reproducibility is paramount in science, being widely seen as a "cornerstone of the scientific method"[6,14]; Popper[15] states that "non-reproducible single occurrences are of no significance to science." The first recorded issue of reproducibility dates back to the 17th century and is described by Robert Boyle in Shapin and Schaffer[16]; an interaction between Boyle, Hobbes, and Huygens concerning an experiment where Huygens observed an "anomalous suspension" of water in a vacuum follows:

> Unless the phenomenon could be reproduced [. . .] then no one [. . .] would accept the claims [Huygens had] made [. . .].

© 2026 The Author(s). Published by Elsevier Inc.
This is an open access article under the CC BY license (http://creativecommons.org/licenses/by/4.0/).

Table 1. A summary of the collected data, where the first number represents the number of applicable reviews and the number of excluded (theoretical) papers is represented in brackets

	AAAI	IJCAI	ICLR	ICML	NeurIPS	JAIR	JMLR
2022	46 (5)	40 (10)	46 (4)	53 (5)	44 (6)	39 (11)	39 (11)
2023	48 (2)	43 (7)	47 (3)	51 (5)	48 (2)	28 (22)	39 (11)
2024	48 (2)	47 (2)	48 (2)	47 (2)	48 (2)	33 (17)	36 (12)
Total	142 (9)	130 (19)	141 (9)	151 (12)	140 (10)	100 (50)	114 (34)

We collected 918 applicable reviews out of 1,061 papers. JAIR has the highest theoretical rate (33.33%) and AAAI the lowest (5.96%). The number of reviews (including theoretical) for IJCAI is less than 150 due to a rejected review (conflict of interest), and slightly more for AAAI/ICML due to a correction of a sampling error of award-winning papers. JMLR had less than 50 publications in 2024 at the time of sampling and is thus lower than 150 in total.

> The accomplishment of replication was dependent on contingent acts of judgment. One cannot write down a formula saying when replication was or was not achieved.

Only two machines existed at the time to reproduce this experiment—one in Boyle and Hobbes' possession and the other in Huygens'. Regardless, Boyle and Hobbes were unable to reproduce Huygens' findings. The solution in this anecdote was to invite the author to their laboratory, a costly but necessary solution. They found that the "accomplishment of replication was dependent on contingent acts of judgement," i.e., Huygens' documentation alone was insufficient for Boyles and Hobbes to reproduce the experiment. Requiring the presence of the original authors is not only a cost-ineffective solution, it is often unrealistic as well; to repeat this scenario today with Huygens' presence would be impossible. Hence, it is obviously necessary to consider reproducibility while documenting scientific findings.

In this work, we consider the cost of reproducibility—the resources required for reproducing the findings of a prior study—and aim to quantify the cost of reproducibility for studies published at top-tier AI venues. Through the quantification of the cost of reproducibility, we seek to understand the impact and effectiveness of reproducibility standards across venues and communities on documentation practices of published AI research. Previous quantification studies on the reproducibility of AI research generally quantified the presence of documentation and their impact on the reproducibility of studies,[8,11,12] but have not accounted for the impact on the increased demands of efforts for the independent investigators. We constructed a rubric score as a proxy for reproducibility cost—based on objective characteristics in the documentation—and conducted a large-scale systematic review of published articles spanning seven major venues between 2022 and 2024.

It would be natural to expect that venues with stricter reproducibility requirements and standards lead to publications that are less costly to reproduce for the independent investigators. Based on our evaluation of the reproducibility cost of 918 AI studies across five dimensions of documentation, we found that stricter venue requirements, such as checklists, have not independently led to easier-to-reproduce articles—their effectiveness is contingent on community support. Furthermore, we find no statistically significant differences between the cost of reproducibility for award-winning papers and other publications; we make recommendations on how the community may be enabled and stimulated to write documentation that results in low reproducibility cost. We also find that externally hosted implementation artifacts are not available due to broken URLs in 6.72% of the time—we recommend venues to archive the implementation as part of the submission policy. We believe that, by understanding what drives the cost, and subsequently reducing it, the reported reproducibility rates of 50.0%[11] and 63.5%[12] could be significantly increased.

RESULTS

First, we analyze the reliability of our results. Second, we analyze the data across seven venues and five dimensions. The dataset is summarized in Table 1, which shows that the number of effective reviews (918) is lower than the total (1,061) due to purely theoretical papers. We create a supplementary dataset based on the dataset of Gundersen et al.[11] and study the correlation between our methodology and reproduction efforts in the section "correlating cost with successful reproduction." We sample award-winning papers specifically and analyzed them separately in the section "award-winning papers"; in all other analyses, these papers were not considered. For all statistical tests, we have used a standard significance level of 0.05. We discuss possible extensions of our data with supplementary data in the supplemental information section "enriching the data set," such as the correlation between reproducibility cost and citation count as shown in Table S3.

Second review

A limitation of our dataset is the lack of reviewing resources. Due to the difficulty of the task, the effort required for reviewing, and the challenge of finding proficient reviewers, initially, only a single review was acquired for each paper by our first author. To mitigate this limitation, we acquired a second review for 46 (i.e., 5.01%) of the 918 papers from independent researchers in our network of PhD candidates and post-doctoral researchers. We let our 14 volunteers select up to five papers for secondary review based on their expertise and personal interest, but were presented with a subset to select from to ensure spread across years and venues.

We calculated the inter-rater agreement using the intra-class correlation coefficient (ICC).[17] Based on the guidelines of Koo and Li,[18] we applied the model ICC3k, which calculates the reliability of our data labeling guidelines over a fixed set of k raters. The labels of agreement from Koo and Li[18] range over "poor," "moderate," "good," and "excellent." Table 2 shows that implementation, data, and configuration dimensions strongly correlate

CellPress
OPEN ACCESS

Table 2. The ICC over the first and second reviews with 95% CI, p value, and their interpreted labels

	MAD	ICC	95% CI	p value	Label
Implementation	0.7609	0.9727	[0.95, 0.98]	0.0000	excellent
Data	0.7826	0.9111	[0.84, 0.95]	0.0000	excellent
Configuration	0.9348	0.9306	[0.87, 0.96]	0.0000	excellent
Experimental procedure	0.7826	0.8072	[0.65, 0.89]	0.0000	good
Expertise	2.0652	0.5445	[0.18, 0.75]	0.0048	moderate

MAD, Mean Average Distance; ICC, intraclass correlation coefficient; CI, confidence interval. Labels based on Koo and Li.[18]

among reviewers, with an excellent label. Experimental procedure has a good label, showing some disagreement but, in general, still a reliable result.

Expertise receives a moderate label, which shows the complexity of assessing this dimension and the disagreement among raters, indicating the need for tangible guidelines; expertise only had loose descriptions with few objective features—a design flaw unique to this dimension, as all dimensions were grounded in the availability of specific documentation such as code, data, and parameter values. Unfortunately, this deficiency did not become evident in our preliminary experimentation. The mean absolute difference shows that the reviewers vary in their assessment of expertise cost by 2.0652, a factor of 2.55 above the other dimensions. Even when accounting for human bias, this is a noisy dimension compared with the others. Thus, we omit expertise in our subsequent analyses to ensure representative and reliable results. We further analyze the reliability of our methodology, with an intra-annotator analysis over a sample size of 62, in the supplemental information section "intra-annotator analysis." Based on the results in Figure S2 and Table S2, we find an overall strong agreement across dimensions and some minor inconsistencies that do not significantly impact our analysis or conclusions.

Analysis per venue and dimension

In Figure 1, our data are visualized per dimension and venue. For the first two dimensions, the percentages of studies containing public implementation or public datasets (the public rate) are indicated by dashed lines. We plot percentages of studies, with each column adding up to 100%, as the absolute numbers vary slightly (see Table 2). The median and quantiles are reported in Table S4. We consider costs in the range of 1–3 to be "low," and those in the range of 7–10 to be "high."

Figure 1 shows that the implementation dimension dominates the other dimensions; even NeurIPS, with a public rate of 80.17%, has 23.97% of studies with a cost of six or higher. Most venues have little middle ground and represent somewhat of a bimodal distribution, which is strongly demonstrated by JMLR, for example; studies either score rather low or high, and this split overlaps with the public implementation rate. This implies that studies that do not publish their implementation, in general, do not seek out to use other facets of documentation, to supplement the absence of code. The configuration dimension dominates the data and experimental procedure dimension, but has a substantially lower distribution than the implementation dimension. For all venues, the majority of studies, third quartile (Q3), have a cost of 5 or lower. The data dimension has a lower distribution, with most venues having cost of up to 4 for Q3; this is, however, not as strongly correlated with the very high overall estimated public data rate of 94.01% as may be expected. We attribute this partially to an inflation of data cost, where authors tend to deem it unnecessary to extensively document commonly used datasets. This is discussed further in the supplemental methods. The experimental procedure is the best documented dimension. All venues have a cost of 3 or less in Q3. This is relatively unsurprising; experiment

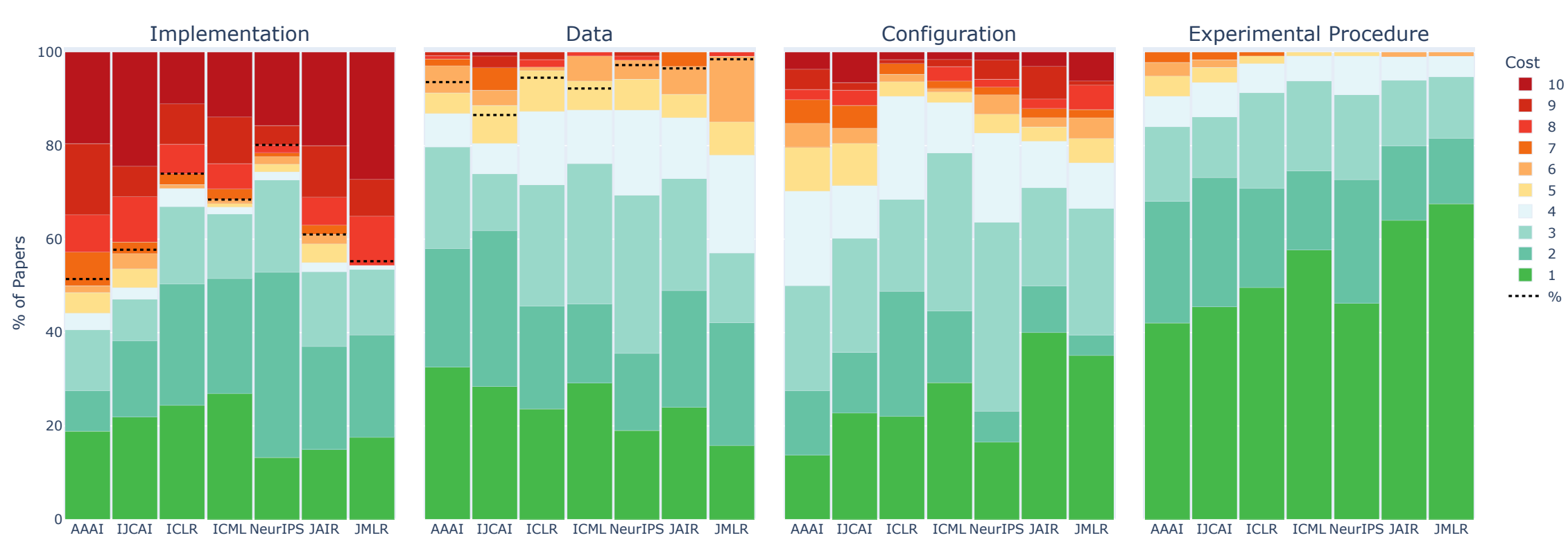

Figure 1. A summary of the collected dataset over seven venues

The first two dimensions have their respective percentages of public code or data plotted with a dashed line; the values are denoted in the caption of each bar. Over all empirical studies, we estimate that 70.52% publish their implementation and 94.01% of used datasets are public. A tabular summary can be found in Table S4.

documentation is an integral part of the scientific method, within the field of AI and beyond.

In order to determine statistically significant differences between venues per dimension, we applied a normality test per venue, per dimension, the results of which can be found in Table S5. We found that none of our data are normally distributed. We thus opt for a one-sided permutation test to determine which studies cost statistically significantly less than another. Statistical tests are discussed further in the supplemental methods. The results of the permutation tests can be found in Table S6.

Implementation

ICLR, ICML, and NeurIPS have a significantly lower implementation cost than the other venues, showing a cost reduction between −1.08 and −1.77 compared with the general AI conferences and between −0.88 and −1.44 compared with the journals. ML conferences tend to have better documented implementations than AI conferences, and papers published there are on average 16.52% less costly to reproduce. Compared with journals, we detected a cost reduction of 12.91% on average. Furthermore, we found that a substantial amount of studies follow a dark pattern: an estimated 6.72% of all implementation URLs are either empty or yield an error after up to 3 years past the publication date. This is not evenly distributed among venues: for general AI venues, the proportions range from 8.20% (JAIR) to 12.68% (AAAI), whereas ML venues range from 3.18% (JMLR) to 5.16% (NeurIPS).

Data

The data dimension is more costly for JMLR than the other venues, except for NeurIPS, although the measured differences are relatively small. As most venues in general do have rather low cost for the majority (Q3) of the studies, the practical significance of the measured differences seems to be low. JMLR has the highest public data rate of all venues (98.51%), yet surprisingly, it is the most costly in this dimension compared with the other venues. We attribute this partially to "popular" (benchmark) datasets. We also find that the majority of studies that rely completely on private data (3.87% of all studies) have a high data reproducibility cost: 84.85% of private data studies have a cost of 6 or higher, mainly due to the collection strategy not being clearly documented. This could be mitigated by, for example, authors creating a datasheet as suggested by Gebru et al.[19]

Configuration

ICLR and ICML have the lowest configuration cost compared with other venues, being significantly less costly than all other venues except for JAIR. Both the journals (JAIR/JMLR) have significantly lower cost than AAAI. The biggest, and thus practically most impactful differences, are between ICLR/ICML and the other conferences: they are on average 11.70% less costly to reproduce. The most interesting outlier here is NeurIPS, a flagship conference of the ML community, yet more costly than ICLR and ICML by 0.64 and 0.66, respectively. As shown in Figure S3, on average, there is a weak correlation between public code and lower configuration cost of −0.21 overall. However, NeurIPS has a substantially weaker correlation (−0.05), indicating that the code is used less often to document the configuration than at other venues.

Experimental procedure

AAAI has a significantly higher cost than all other venues except IJCAI, whereas IJCAI has a significantly higher cost than ICML, JAIR, and JMLR. NeurIPS is significantly more costly than both journals. All distances are relatively small, ranging from −0.27 to −0.65. Thus, the practical impact of the differences is low. This is also shown by the fact that each venue has a low experimental procedure cost for the majority of studies (Q3) and that no studies with a high experimental procedure cost have been found. The studies we surveyed document this dimension well, and although there is room for improvement, this is marginal compared with the other dimensions.

Correlating cost with successful reproduction

Our evaluation has been primarily focused on the quality of documentation and its expected impact on unnecessary labor in reproducing research studies. To determine how our estimated costs directly correlate with the reproduction of a study, we rely on the dataset of Gundersen et al.,[11] where the authors published the success rate of reproducing studies using public data within a 40-working-hour time limit. We also aimed to include a similar analysis on the results of Stodden et al.,[20] but this was rendered infeasible due to unavailability of the data. Gundersen et al.[11] recorded both working hours and various problem types per reproduction attempt. There are several biases to be accounted for; the authors only included studies with public data, the working hours used for reproducing research were limited to 40 per study, the studies were selected from a different time frame than the one considered here, and the studies were selected based on the number of citations, whereas ours were randomly drawn. An extensive discussion can be found in the supplemental information section "correlation with Gundersen et al. (2025)."

A straightforward approach would be to correlate our cost metric with the recorded working hours per reproduction attempt. However, the dataset of Gundersen et al.[11] is relatively small and offers little statistical power for such an analysis; in two cases, the authors determined that no results could be acquired before the time limit, due to lacking information, thus limiting the explanatory value of the recorded labor hours in relation to the quality of documentation. Secondly, the majority of the attempts (14 of 22) ran into the time limit, both in success and failures, making the recorded working hours variable rather constant. Thus, we reframe our question toward the data as follows; how strongly are our estimates of reproducibility cost correlated with reproduction success within a limit of 40 working hours? We hypothesize that higher costs imply lower success rates; we expect to observe a negative correlation coefficient between the cost variables and success rate variables.

We applied the Kendall correlation coefficient to determine the relation between the dimensions we consider and the success rates of the reproduction attempts. We analyzed the number of experiments successfully reproduced, the number of experiments performed overall, and whether the study was considered successfully reproduced or not. We also consider the *number* of experiments completed, regardless of the outcome; the number of experiments completed is still a form of success even if the result was inconclusive, albeit less positive than the other success metrics. The results are found in Table 3. Based on our hypothesis, we expect an inverse correlation: the higher our cost, the lower the reproducibility. In Table 4 we can see that across the board, the dimensions show moderate correlations with the three properties,

Table 3. The Kendall correlation coefficient between our dimensions and the recorded reproduction success on a sample dataset

	% Experiment completed	% Experiment reproduced	Success
Implementation	**−0.31** (0.03)	**−0.37** (0.03)	**−0.37** (0.02)
Data	−0.08 (0.32)	−0.01 (0.49)	0.07 (0.65)
Configuration	**−0.38** (0.01)	**−0.32** (0.03)	**−0.32** (0.05[a])
Experimental procedure	−0.18 (0.16)	−0.16 (0.19)	−0.12 (0.28)

Based on the dataset from Gundersen et al.[11] The second column denotes how many experiments were completed in total (including inconsistent results), and the third column denotes how many experiments of the original authors were successfully reproduced (identical or consistent results). The fourth column denotes whether the reproduction attempt was considered successful ("S" or "PS") or not ("NR" or "F").
The p values of each correlation are placed in brackets, and significant results are marked in bold.
[a]The measured p value was 0.0467, rounded to 0.05 in the table.

except for the data dimension. This is easily explainable: the data dimension has an overall low score, due to Gundersen et al.[11] selecting studies with only publicly available data, which generally implies a low cost by our methodology. This variable is thus less insightful or predictive of successful reproduction attempts in this dataset. The eight samples of the dataset that use private data have been excluded in our analysis, as no reproduction attempt was undertaken for these studies. The implementation and configuration dimensions have moderate and significant correlations for all three metrics; implementation has a slightly stronger correlation for reproduction and successful outcome, whereas configuration has a slightly stronger correlation with regard to whether the independent investigators were able to conduct an experiment based on the documentation.

The Experimental Procedure dimension has no significant correlations with any of the measurements. This can be partially explained by the fact that overall, the studies in the dataset of Gundersen et al.[11] scored quite well in this dimension (the highest recorded value was 4), similar to our results in the section "analysis per venue and dimension." We did not expect a single dimension to show a ""near-perfect" correlation with success; lack of documentation can occur in any dimension, and the dimensions may have a joint impact on whether a study can successfully be reproduced or not. Furthermore, we found that of the problems that occurred during the study of Gundersen et al..[11] only a few are (partially) related to the Experimental Procedure (P1, P4, P9, P10, and P13), and many reproduction attempts that encountered such a problem still resulted in successful reproduction. These problem types have a strong relation with our work and can give concrete insights into the limitations of our method, which we discuss in detail in the supplemental information section "correlation with Gundersen et al. (2025)" and expand upon in the supplemental information section "analysis on TMLR reproducibility studies" with 39 additional samples and 10 new problem types (Table S1) acquired from TMLR.

Award-winning papers

We also reviewed 65 award-winning papers, i.e., best or outstanding papers, at AAAI, IJCAI, ICLR, ICML, and NeurIPS. JMLR and JAIR were excluded from this as JMLR does not present such awards and JAIR only has a single award-winning paper in our time frame. To test whether award-winning papers are drawn from statistically significantly different distributions when compared with "regular" papers, we applied the Kolmogorov-Smirnov test to check if they are drawn from the same distribution and the permutation test to check if the cost is significantly lower. The results are found in Table 4. Our results suggest that only IJCAI shows a single significant difference according to the Kolmogorov-Smirnov test and only ICML best papers show a significant reduction in cost for the data dimension compared with average papers, an already well-documented dimension. We find no strong evidence that the cost of reproducing an award-winning paper is lower than an average paper published at the same venue.

DISCUSSION

According to our results, the two most ill-documented dimensions are implementation and configuration. Gundersen et al.[11] found that the biggest bottleneck for reproducible research in AI is the accessibility of data and code. As the data dimension is generally associated with a low reproducibility cost—driven by a high proportion of public datasets across the board—we conclude that the community has good standards in this regard. However, we do note that our study does not include a separate analysis of the documentation of datasets in the original publications, which we leave for future work. The second most important dimension, according to the findings by Gundersen et al.,[11] implementation, is the least well documented of all. For example, 6.72% of all provided implementation links are either empty or broken. In this respect, we see room for improvement for all venues, but papers published at ML conferences appear to be significantly less costly to reproduce than those at other venues with respect to the implementation dimension.

The configuration dimension is the second least well-documented dimension, and we find that publications at ICLR and ICML are less costly to reproduce with regard to the configuration dimension than those from other venues, except for JAIR. The most noteworthy outlier here is NeurIPS, a flagship of the ML community, yet separated from ICLR and ICML. Since ICML and NeurIPS share the same checklist, yet employ a different protocol around its use, this finding suggests that the effects of venue reproducibility standards on the actual reproducibility costs are more nuanced than we initially hypothesized.

All venues employ reproducibility standards in their paper submission process (AAAI, IJCAI, ICLR, NeurIPS, ICML, JAIR, and JMLR), and the effectiveness of the different approaches is interesting: NeurIPS has the strongest requirements, and since 2024, it is mandatory for all authors to append the reproducibility checklist to their main papers published there. This is in strong contrast to ICLR, which only "strongly encourages" a paragraph-long reproducibility statement. For the year 2024, we found that 98.84% of the accepted papers at NeurIPS included the checklist in the main paper, whereas only 15.79% of the accepted papers at ICLR included the optional reproducibility statement.

However, ICLR achieves similar results as NeurIPS in terms of documentation quality with far softer requirements for authors.

This difference is hard to explain based on our dataset. An important factor that could contribute to this effect is that the participants of ICLR and NeurIPS are all part of the larger ML community. Thus, standards set out by either conference chairs or its participants can easily influence other sub-communities. This effect can directly occur through resubmission: papers rejected from one conference often find a home at another one later; thus, multiple sets of standards can affect the documentation quality of a given paper. Another important factor for ICLR is its use of a public review process, allowing both reviewers and other members of the scientific community to read and respond to the submitted work. How often these broader interactions play a role in adapting the documentation of submitted work is unknown to us, as is how authors adapt their work before submission, considering that it will become publicly visible at that point. This public reviewing process only applies to ICLR and warrants an analysis of its own to determine its role in reproducibility and whether it should be recommended to other venues.

The general AI conferences have the highest reproducibility cost across the board, with a few exceptions in the data dimension. These conferences have different reproducibility standards, and most importantly, they are the only venues that do not allow for appendices in accepted publications. Appendices are frequently used by authors to document key information; we found that, for the ML conferences in approximately 69.23% of the empirical studies, the appendix contained information for one or more dimensions. However, the availability of appendices is not guaranteed to lead to better reproducibility documentation; publications at JAIR and JMLR are less costly than those at AAAI and IJCAI in terms of configuration and experimental procedure, yet the differences are marginal. We argue that the longer format enables authors to provide in-depth documentation, but that the sheer availability of them does not serve as a stimulus for better documentation. Another reason that general AI conferences are more costly than others is that they might suffer, on average, from different standards in different sub-communities in AI, which should be studied in the future.

Limitations of the study

We attempted to evaluate the expertise required for reproducing a paper but failed to do this reliably, as described in the section "second review." This dimension was intended to represent a more intangible cost but turned out to be impractical, due to the lack of objective guidelines enabling objective assessment. As an alternative approach, a quantification study to determine the impact of the experience of independent investigators on the effort required to reproduce a given study would, nonetheless, likely provide valuable insights, yet it remains an open question how to properly measure and quantify this experience; we leave this for future work.

Another limitation of our study is the sample size, as the process of evaluating the quality of documentation without relying on objective but simplified metrics (e.g., as done in Gundersen and Kjensmo[8]) is difficult to automate. Thus, we had to rely on human annotation for a complicated and time-expensive task; this resulted in only a small proportion of all published papers of each venue being analyzed, but we argue that the sample size of 150 per venue is statistically robust. Note that our sample size is not proportionally adapted to each venue; this has no impact when analyzing the venues individually, but it requires weighting when determining population wide trends. This is process is described in the supplemental information section "assumptions on the entire population."

Finally, we also estimated how strong our dimensions correlate with actual reproduction attempts by Gundersen et al..[11] We found statistically significant correlations between the two dimensions, implementation and configuration, and the success measures. One limitation of this analysis is that the dataset only contains studies with public datasets; hence, we were unable to reliably determine the relation of the data dimension and successful reproduction. Furthermore, the studies in the dataset by Gundersen et al.[11] represent the most cited works between 2011 and 2016 and are thus less representative of the entire population of studies published within that period of time. Lastly, this dataset is relatively small, with only 22 samples, thus limiting the statistical power of our analysis, which is a common issue with meta-science studies. To mitigate the sample size limitation, we expand upon this dataset with 39 reproducibility studies published in TMLR in the supplemental information section "analysis on TMLR reproducibility studies"; we find, on the basis of Figure S1, (1) that our methodology captures 80.86% of the reported issues in reproducibility studies; (2) that contacting authors for supplementary information is unsuccessful in 36.84%, elucidating the need for comprehensive documentation; and (3) that there is a possible bias of reproduction attempts toward studies that use public data.

Conclusion

In this work, we reviewed 1,061 papers from seven major AI/ML venues over the past three years and analyzed 918 on five proposed dimensions of reproducibility cost. We found significantly lower costs for ML conferences compared with other venues. We attribute this to the differences in venue policies—stricter reproducibility standards and longer formats of papers through appendices. We recommend that venues require the inclusion of reproducibility checklists in their submissions and allow appendices in publications to enable in-depth documentation.

However, we also find that compliance-based mechanics, such as checklists, are not effective independently, as shown by ICLR and NeurIPS as well as the general AI venues and journals; community changes are needed as well. While, in principle, increased effectiveness of such mechanics could be achieved through a stricter review process, this solution may be unrealistic in practice, as the process already demands significant time per reviewer. Venues may consider supplementing the review process by assigning a "reproducibility reviewer," e.g., as done by the AutoML conference. This can distribute the workload and bring a dedicated reproducibility focus to the review process, and thus stronger enforcement of standards.

We found little evidence that award-winning papers are less costly to reproduce than average papers. As awards bring a spotlight to selected publications, we strongly recommend strengthening the role of reproducibility in the criteria used for adjudicating awards, to drive community changes through highly visible examples of best practices and increase the effectiveness of these measures. Alternatively, venues could include a "reproducibility papers" track, as, found in e.g., ACM MM or ECIR. Another stimulant for reproducibility is the use of workshops

CellPress
OPEN ACCESS

and challenges such as the Reproducible AI workshop or the Machine Learning Reproducibility Challenge. Hosting such tracks, workshops, or challenges places a highlight on reproducibility and enables the publication of reproduction studies.

Our study also revealed that a significant number of implementation URLs (6.72%) are empty or broken. Thus, one venue policy that may prove effective independently, is requiring authors to submit their implementation as supplementary material rather than only providing an external link in the article—archiving could reduce the erroneous implementation link rate substantially.

We hypothesized that studies published at venues with stricter requirements will be less costly to reproduce. We found only partial support for this hypothesis; although venues with stricter requirements generally cost less, we found more factors that play an important role regarding whether strict requirements are effective; community support and stimulation are important factors whether these standards affect documentation quality—without such stimulus, compliance-based mechanisms may be largely performative. Although we do not regard the reproducibility crisis in AI as solved, our work provides insight into the effectiveness of reproducibility mechanisms, and through our data-driven findings, we offer actionable pathways toward achieving positive structural, procedural, and community changes to mitigate this crisis.

METHODS

In this section, we first address and discuss the definition of the term "reproducibility" as used in this work in the section "background." Second, we define the methodology used to obtain the results in the section "methodology." Last, we discuss literature related to this work in the section "related work."

Background

Although scientists generally agree that reproducibility is fundamental to the scientific method,[6,14] definitions found in the literature vary, and terms such as replicability and repeatability have been used as synonyms for reproducibility. Notably, over the past three decades, with the increasing prominence of computer science and automatization, there has been a tendency to define reproducibility as re-computation[21–23]—a definition we find too narrow, as it reduces the reproducibility of methods and results to a subset of the documentation and the execution of computer instructions. Buckheit and Donoho[24] limited reproducibility to reproducing the figures of the original authors using their source code, which can be useful for comparison, but is only a single step in the analysis of the results, which itself represents only one part of the scientific method.

These conflicting notions were recently clarified by the Association of Computing Machinery (ACM)[25]—motivated by the lack of uniformity and clarity on the meaning of reproducibility across the field of computer science. This discussion of terminology was continued by the National Academies of Sciences, Engineering, and Medicine (NASEM),[26] where the authors provide a comprehensive summary on the subject and overall settle on the definition of reproducibility similar to that by the ACM: "*reproducibility* is obtaining consistent results using the same input data; computational steps, methods, and code; and conditions of analysis." While this definition provides a clear outline of the term and successfully resolves prior ambiguities, like other definitions, it introduces restrictions but more importantly omits a definition on the *objective* of reproducibility—essential in our work to understand what any "cost" is to be paid for.

In this work, we use the term reproducibility as defined in Gundersen[27]:

> Reproducibility is the ability of *independent* investigators to draw the same *conclusions* from an experiment by following the *documentation* shared by the original investigators.

In this definition, the terms shown in italics are to be interpreted as follows:

Independent: investigators who have no conflict of interest with the original work and have no access to information limited to the original investigators.

Conclusions: drawing the same conclusion can be based on producing the same outcome, performing the same analysis, or arriving at the same interpretation of the analysis.

Documentation: any work produced, written, or otherwise, provided by the authors, including supplementary material of a publication. This can include, but is not limited to, appendices, code written or used by the authors, data, and other (digital) artifacts.

The definition of Gundersen[27] deviates from that of NASEM,[26] and by extension ACM.[25] Namely, the definition of NASEM[26] does not permit any sort of variation in data or code, which we find overly strict. Furthermore, when independent investigators draw the same conclusions as the original authors, they add *credibility* to the original method; the definitions of the ACM[25] and NASEM[26] do not align with this view—one can easily construct a counterexample, where different code and/or data have been used to conduct, in spirit, the same experiment as the authors and thus reproduce their findings. Thus, we conclude that the open definition of Gundersen[27] aligns more closely with the objective that motivated this work.

Raff et al.[28] also drew attention to the problems of confusing terminology when it comes to reproducibility, specifically within ML (we consider ML to be part of the broader field of AI[29]) research; 45% of the surveyed papers use the terms reproducibility, repeatability, and replicability interchangeably. Using the definitions of repeatability, reproducibility, and replicability of the ACM,[25] they proposed to extend the terminology with five types of rigor: (1) adaptability, the study of applying an existing implementation to different data; (2) model selection, the study of how to conclude whether one method outperforms another; (3) label/data quality, the reliability of data acquisition; (4) meta & incentive, the study of incentives of scientific rigor; and (5) maintainability, which considers the factor of time as degradative to code. Within the definitions of Raff et al.,[28] our work is best categorized as a meta & incentive study, as we seek to understand what factors drive the cost of reproducibility in specific venues and communities—a field of meta-science "very few papers have studied."[28]

An extended discussion on the terminology of reproducibility used in other fields, as found in Goodman et al.[30] and Crook et al.,[31] is provided in the "extended background" section of the supplemental information.

Cost of reproducibility

In this work, we evaluate the cost of reproducibility in AI research—the resources that are required to reproduce the findings of a prior study. These resources may vary per study but will always include some time resources of the independent investigator. This demand of time resources can be significantly impacted by, for example, the absence of code. When the independent investigator must reimplement the method, the reproduction attempt becomes far more costly than a study that provides code that only has to be run. Analogously, the unavailability of data will require the independent investigator to acquire a substitute dataset or reacquire the original. This will have a substantial impact on the time resources required, thus raising the cost of reproducibility. As evidenced by the estimated reproducibility rates,[11,12] this cost is often too high for independent investigators to practically reproduce published AI research. Here, we investigate how reproducibility cost varies across venues and communities—to understand how much effort is involved in reproducing studies from the recent AI literature. We specifically focus on top-tier venues because of the high impact of the empirical studies published there.

Based on our definition of reproducibility, we investigate the cost based on the documentation provided by the authors. To isolate this role, we design our methodology around the materials provided by the authors during the peer-review process for each venue. We formulated criteria to detect critical gaps in the provided documentation, to quantify the cost from five distinct aspects of documentation. This will enable us to determine possible trends and variations between publication venues and research communities within AI, providing data-driven conclusions to what practices should be encouraged or avoided. We describe this methodology in the section "methods."

We are not the first to address the cost of reproducibility. Poldrack[32] describes the costs as the efforts and struggles for early-career researchers (ECR) within the field of neuroscience to ensure the reproducibility of their work. It highlights the difficulties for ECRs to produce and write quality research that adopts more reproducible practices. These key actors within neuroscience, such as the need for resources or data as well as the impact of the "publish or perish" mentality, are present in our field as well and play a detrimental role to the reproducibility of published research. The distinction between our work and that of Poldrack[32] is that we consider cost as the impact on independent investigators, in contrast to factors affecting authors to produce less-reproducible research.

Methodology

As stated in the section "background," we aim to identify criteria with regard to the documentation that correlate with the effort required for a reproduction attempt by independent investigators. We analyze it from the perspective of the original authors—costs that may be mitigated by the original authors. Factors that can increase the cost of reproducibility, such as requirements for computing resources, therefore fall outside of the scope of our analysis.

Based on the findings of previous quantification[8,11,12] and methodological[27,28] studies on reproducibility in AI, we propose five dimensions of documentation of AI research. Each dimension addresses discrete elements of the documentation that are orthogonal to each other and impact a different part of the reproducibility effort: (1) implementation—regarding the presented methodology and specifically how it is to be computationally executed; (2) data—regarding the input data used to obtain experimental results; (3) configuration—the algorithmic parameter settings used to produce the results; (4) experimental procedure—under which protocol the presented values were acquired; and (5) expertise—how much background knowledge the independent investigator is expected to have.

Implementation cost

Given the documentation shared by the authors on a new method, how much effort would it be to re-implement the method from scratch? The cost of implementing a method mainly considers publishing the source code used for the results of the study. By publishing the source code or the method as part of a publicly available software package, the authors can lower the cost of re-implementation and facilitate running the same or similar experiments and enable independent researchers to determine possible improvements in computational efficiency or alternative methods. Publishing the implementation can also be substituted, augmented, or enhanced with other materials, such as pseudo-code, practical descriptions, or designs and diagrams.

Data cost

Given the data description in the documentation, how much effort would it take to either find the same dataset the authors used, or a similar dataset and defend the comparability, or acquire one from scratch? The cost of acquiring data when reproducing a method and results is one of the most persistent problems in the reproducibility of AI research. We aim to evaluate how well authors document which (publicly available) data they used, to be able to conduct a comparable experiment. Furthermore, we acknowledge that publishing data is not always possible, e.g., in fields such as medicine. However, the method of data acquisition should be clearly documented, such that independent investigators can explain the comparability with other pre-existing datasets or acquire new data with similar characteristics.

Configuration cost

Given the (hyper)parameters, including semantic parameters, of the method, how much effort would it take to acquire the algorithm configurations used for obtaining the reported results and compare them against their computation budget? The acquisition of algorithm configurations and hyperparameter values is mainly a computationally costly endeavor, and the budgetary constraints under which they were achieved can yield substantial variation in the results and subsequent conclusions. By documenting the used values and under which conditions they were acquired, independent investigators can reproduce results efficiently, and defend why these results can be used to conduct a comparable experiment. This includes documenting semantic parameters, e.g., parameters related to the task not impacting outcome quality, such as the number of output nodes in a neural network.

Experimental cost

Given the experiment description in the original work, how difficult is it to set up a new experiment with the same procedure? The cost of setting up the same, or a comparable, experiment is crucial for evaluating the method. This requires documentation on the strategy and workflow, which metrics are used, and how

the results are interpreted to draw conclusions. Lack thereof may significantly impact the outcome of the experiment, leading to (statistically) incorrect results and conflicting conclusions.

Expertise cost

How much effort would it take to acquire the expertise required to reproduce the work independently? Acquiring expertise to reproduce a study may vary both on the side of the independent investigators' experience and the complexity of the study. We unify this by considering the cost of the acquisition of the prerequisite expertise, independent of whether this has been done. This may be caused by the need for knowledge from specialized subfields, requirements of inter-domain knowledge in applications and the understanding of mathematical theory.

Assessing the cost

To quantify each dimension of reproducibility cost, we answer the questions of each dimension with a score ranging from one to ten, where one indicates very low cost and ten indicates very high cost. For each paper, we limited the documentation to all materials supplied by the publisher (e.g., the paper itself and possible supplementary materials) and any external links directly provided there (e.g., a link to a GitHub repository). No other materials were searched for online, to ensure a fair and comparable evaluation for each publication. We decided on a positive approach for the study and assumed all required documentation was provided; thus, each review starts with a cost of **1** for all dimensions.

We evaluate each dimension based on our guidelines that cover a wide range of topics allowing for the expression of a variety of topics across studies and subfields. Implementation cost is focused on practical method documentation, such as pseudo-code, diagrams, design choices, and libraries or systems used for creating the implementation as well as verifying the code presence/quality when available. Data cost is focused on all datasets used and is averaged with a weight of one per dataset by default, but we permitted reviewers to adapt these weights on a case-by-case basis. Datasets were evaluated on their descriptions regarding their task and statistics, with ample documentation requirements regarding acquisition when private. Synthetic data or simulated environments were evaluated separately, with a focus on their implementation and parameters. Configuration cost is focused on clear and structured explanations of what the authors considered parameters of their method, using, for example, pseudo-code or tables; what values were used in each experiment; and how they were acquired. The importance of parameters is taken into account when estimating the cost. Experimental cost is focused on the metrics used and the population representation, including the type of error bars, aggregation functions, and the evaluation strategy used, along with possible parameters. Expertise cost is mainly focused on how many subfields are used and to what extent and how well these are introduced. We define a set of criteria and associated penalties and provide a summary in Table 4. For brevity, a detailed specification can be found in the supplemental information, in the section "criteria examples."

As we aim to estimate the cost of reproducibility, solely based on the provided documentation, we measure the cost indirectly; our rubric score represents the quality and comprehensiveness of the documentation of a study. This also entails that our metrics are capturing documentation quality as a *proxy* for reproducibility itself; it is possible for a well-documented paper to be irreproducible—a simple example would be a case of fraudulently manipulated results. Nonetheless, we would expect the independent investigators to reach the conclusion of *irreproducibility* with less effort when comprehensive documentation is given. Furthermore, even with complete documentation, practical issues may still surface during a reproduction attempt, thus increasing the effort required; we study this separately in the supplemental information, in the section "analysis on TMLR reproducibility studies."

Further details regarding the methods can be found in the supplemental methods. Our full guidelines, dataset, and code can be found in our repository.[33]

Paper sampling

We sampled studies from five major conferences and two major journals from AI and ML over the past 3 years (2022–2024), two general AI conferences (AAAI and IJCAI), three ML conferences (ICLR, ICML, and NeurIPS), and one general AI and ML journal each (JAIR and JMLR). We selected these venues based on their broad scope, excellent reputation, and high impact on the scientific community. Per venue, we reviewed about 50 papers per year, selecting award-winning papers and sampling the remaining papers uniformly at random. We reviewed each paper according to our guidelines, noting the implementation link and the amount of (public) datasets in each work as metrics. Only regular papers were included in the sampling, e.g., excluding survey papers and position papers, as they generally do not provide an empirical evaluation. We also excluded papers that were originally present in another venue to avoid over-representation (i.e., journal versions of conference papers). Theoretical papers were included, but purely theoretical papers (e.g., no empirical evaluation) were marked and excluded from our analysis as the reproducibility of purely theoretical work poses challenges of a different nature. More details on the sampling strategy can be found in the "data collection" section of the supplemental information.

Related work

Gundersen and Kjensmo[8] as well as Gundersen et al.[34] conducted a quantification study regarding reproducibility. They used 16 Boolean and categorical variables over three categories, namely method, data, and experiment, to analyze the state of reproducibility in two conferences over 2 years each. They reviewed in total 400 papers, of which 325 were applicable for the study. The variables were engineered to the quality of documentation on the method, availability of data, results, and code. Based on their degrees of reproducibility, the authors concluded that none of the papers are "fully reproducible," but that there was evidence for a statistically significant improvement over time. Although similarities are present between our methodology and that of Gundersen and Kjensmo,[8] we approach the problem from a distinctly different perspective—we consider the effect of the collective documentation on the reproducibility effort rather than the mere presence of individual elements such as the presence of a code repository URL or the presence of a pseudo-code. Hence, we required a more expressive representation to capture the cost of reproducibility. The resulting scores allow

Table 4. A summary of our data collection guidelines

Dimension	Subset	Description	Cost
Implementation	code	bad readme (dependencies unclear, bad examples)	+1
	code	lack of comments	+1
	code	lack of repository structure	+1
	no code	no (subset) of code given	+4
	no code	no statements on framework/libraries/ languages, etc.	+4
	no code	some details on framework/libraries/ languages, etc.	+[2,3]
	no code	extensive practical details including design choices	+1
	no code	statements of implementations used for their method	+0
	overview	pseudo-code, designs, or architecture missing	+[1,2]
	miscellaneous	no implementation, but other key details provided	−[1,2]
Data	meta	link to dataset not provided	+1
	meta	dataset citation not given and no link	+1
	details	dataset description lacking	+1
	details	dataset statistics lacking	+1
	acquisition	dataset (partially) private	+[1,2]
	acquisition	private dataset with unclear collection strategy	+[1,3]
	miscellaneous	dataset is only named	+[0,5]
	synthetic	code not provided	+[0,5]
	synthetic	process/task not described	+[1,2]
	synthetic	parameters not clear	+[1,3]
Configuration	parameters	no clear overview/summary	+[1,3]
	parameters	values not specified	+4
	parameters	values not clear per experiment	+[1,2]
	acquisition	strategy not clear	+1
	acquisition	budget not clear	+1
Experimental procedure	strategy	strategy not clear	+[1,2]
	strategy	strategy parameters not clear	+1
	metrics	used metrics not clear	+[1,2]
	metrics	aggregation/population not clear	+1
	metrics	population variation not clear	+1
	data	split for training/testing unclear	+2
	results	data (subset) unclear	+1

In brackets, we express a range of options to summarize various sub-criteria. Each guideline is specified per dimension and subset. Not all guidelines are applicable to any given study. When multiple datasets are used, the value is a weighted average, with weights set to one by default. Further details can be found in the supplemental methods, and complete guidelines are provided in the supplemental information.

for a more fine-grained assessment and capture the presence of the documentation elements in the context of each study. Furthermore, we separate the experimental setup and implementation of the method entirely, whereas the experiment category of Gundersen and Kjensmo[8] covers both. The reason for this separation is that we find these two elements to serve two fundamentally different purposes—the ability to implement or execute the presented method versus the experimental protocol under which the method is evaluated.

Raff[12] defined 26 features over three types (unambiguous, mildly subjective, and subjective) and collected values for these from 255 ML papers, published between 2012 and 2018, that he attempted to reproduce. He collected various features, including the number of plots and tables in the paper, the availability of code, the specification of hyperparameter settings, and author responsiveness to inquiries—some of which coincide with our method. He showed the correlation between the collected features and whether the paper was reproducible and thus

CellPress
OPEN ACCESS

determined that 10 features are significantly correlated. In contrast to our study, Raff[12] focused on papers that have already been (attempted to be) reproduced and were selected based on "historical interest," thus making the population of papers reviewed not necessarily representative of published studies in ML, let alone the broader field of AI. Second, we aim to quantify the cost of reproducibility, whereas Raff[12] sought to answer the question of what makes a study reproducible when no code is available and assessed them accordingly. The latter is a key methodological difference; Raff[12] explicitly stated that he does not use source code as part of the documentation of a paper to answer his research question, whereas we argue that it is a key element of the documentation.

Stodden et al.[20] conducted a large-scale reproducibility analysis to determine the efficacy of journal policy standards on computational reproducibility in the field of mathematical computational sciences. To measure this effect, they randomly sampled 204 scientific papers published in *Science* in 2011 and 2012, and they quantified their expected reproducibility using the standards of the Institute for Computational and Experimental Research in Mathematics workshop in 2012 (ICERM) workshop; they additionally acquired a pre-policy sample (2009–2010) to examine the difference in available artifacts. Stodden et al.[20] deemed 56 publications "likely to be reproducible," and randomly selected 22 for a reproducibility attempt, finding all but one to be reproducible. This outlier relied on a large community dataset and documented links for software, which was no longer available.

The standards of the ICERM largely overlap with our criteria for a low reproducibility cost: the presence of parameter settings, details on auxiliary software, availability of code with separate criteria for quality of documentation thereof, and availability of input/output data. There are two criteria that are not present in our methodology: precise statements of assertions made in the paper and a full statement or valid summary of the experimental results. Precisely stating findings and documenting under which assumptions they are made are crucial for reproducing research. We rather focus on "salient details," key information that is easily overlooked but substantially impacts the reproducibility effort, rather than the framing of claims; this focuses on what a study concludes rather than how the empirical evaluation is documented. We separated these two distinct elements in our definition of reproducibility in the section "background." Nonetheless, there is substantial overlap between our work and that of Stodden et al.[20] We contacted the authors to request the data to carry out a direct comparison of our methodology with their reproduction attempt findings; however, the results of the survey in Stodden et al.[20] are anonymized, due to restrictions by the institutional review board, rendering a direct comparison with this study and ours impossible.

In a more recent approach, Gundersen et al.[11] presented a systematic replication of 30 AI studies (10 publications from 2012, 2014, and 2016 each). The authors selected the most highly cited publications and only included papers where either data or data and code are available, based on the reproducibility types of Gundersen et al.[35] This yielded 22 papers to be studied, the results of which represent the reproducibility of the most impactful research. They found a shockingly low (partial) success rate of 50% within a time limit of 40 working hours. The working hours measured in their study have an important link with our work, as it is a direct expression of labor, i.e., the effort required to reproduce. The average successful reproduction, including partial success, took the authors 34 h out of a 40-h time limit. They encountered problems during this process that increased the time cost, and 17 of 20 encountered problems directly related to a lack of ambiguities in documentation. We cross-analyze the outcomes of Gundersen et al.[11] with the outcomes of our methodology in the section "correlating cost with successful reproduction." An extension of this section can be found in the "extended quantification studies" section of the supplemental information.

RESOURCE AVAILABILITY

Lead contact

Requests for further information and resources should be directed to and will be fulfilled by the lead contact, Thijs Snelleman (snelleman@aim.rwth-aachen.de).

Materials availability

There are no materials generated within this work.

Data and code availability

We document the methodology used to produce our dataset in the section "methods" and the supplemental methods. The full guidelines, code, and dataset can be found in our repository.[33] Original data have been deposited to Zenodo: https://doi.org/10.5281/zenodo.21032062. This includes our code used to create our tables and figures for analysis. The few parameters we use, such as the amount of re-samplings in the permutation tests, are documented throughout our paper and are included in our code notebooks as well.

ACKNOWLEDGMENTS

We would like to thank our colleagues from our research group, specifically Justin Dettmer, Julian Dierkes, and Roman Kalkreuth, for the valuable input regarding the data collection process setup and the following for their input regarding secondary reviews: Nick Kocher, Hadar Shavit, Anja Jankovic, Henning Duwe, Bram Renting, Wadie Skaf, Annelot Bosman, Laurens Arp, Guus Toussaint, Marie Anastacio, Anna Muenz, and Konstantin Kaulen. We would like to thank the reviewers of our manuscript for their substantial, thorough, and constructive feedback, providing us with the opportunity to improve our work. Furthermore, we would like to acknowledge financial support through the Alexander von Humboldt Professorship in Artificial Intelligence held by H.H. and the Norwegian Research Council under the Robust Intelligent Control project (RICO, 329730T).

AUTHOR CONTRIBUTIONS

T.S. was responsible for conceptualization, data curation, software, formal analysis, validation, investigation, visualization, methodology, writing (original draft and review & editing), and project administration. H.H. was responsible for formal analysis, supervision, funding acquisition, project administration, and writing (review & editing). O.E.G. was responsible for conceptualization, formal analysis, supervision, funding acquisition, validation, investigation, methodology, project administration, and writing (review & editing).

DECLARATION OF INTERESTS

The authors declare no competing interests.

DECLARATION OF GENERATIVE AI AND AI-ASSISTED TECHNOLOGIES IN THE WRITING PROCESS

During the preparation of this work the authors used Google Gemini in order to search for synonyms for stylistic purposes and precise terminology. After

using this tool/service, the authors reviewed and edited the content as needed and take full responsibility for the content of the published article.

SUPPLEMENTAL INFORMATION

Supplemental information can be found online at https://doi.org/10.1016/j.patter.2026.101640.

Received: February 9, 2026
Revised: May 15, 2026
Accepted: July 23, 2026
Published: August 27, 2026

REFERENCES

1. Baker, M. (2016). 1,500 scientists lift the lid on reproducibility. Nature *533*, 452–454. https://doi.org/10.1038/533452a.
2. Schmidt, S. (2009). Shall we really do it again? The powerful concept of replication is neglected in the social sciences. Rev. Gen. Psychol. *13*, 90–100. https://doi.org/10.1037/a0015108.
3. Junk, T.R., and Lyons, L. (2020). Reproducibility and Replication of Experimental Particle Physics Results. Harv. Data Sci. Rev. *2*. https://doi.org/10.1162/99608f92.250f995b.
4. Ciriminna, R., Angellotti, G., Petri, G.L., and Pagliaro, M. (2024). Reproducibility in chemistry research. Heliyon *10*, e33658. https://doi.org/10.1016/j.heliyon.2024.e33658.
5. Tiwari, K., Kananathan, S., Roberts, M.G., Meyer, J.P., Sharif Shohan, M.U., Xavier, A., Maire, M., Zyoud, A., Men, J., Ng, S., et al. (2021). Reproducibility in systems biology modelling. Mol. Syst. Biol. *17*, e9982. https://doi.org/10.15252/msb.20209982.
6. Moonesinghe, R., Khoury, M.J., and Janssens, A.C.J.W. (2007). Most published research findings are false-but a little replication goes a long way. PLoS Med. *4*, e28. https://doi.org/10.1371/journal.pmed.0040028.
7. Hill, D.R. (2017). Numerical reproducibility of parallel and distributed stochastic simulation using high-performance computing. In Computational Frameworks, M.K. Traoré, ed. (Elsevier), pp. 95–109. https://doi.org/10.1016/B978-1-78548-256-4.50004-1.
8. Gundersen, O.E., and Kjensmo, S. (2018). State of the art: Reproducibility in artificial intelligence. In Proceedings of the 32nd AAAI Conference on Artificial Intelligence, *32*, pp. 1644–1651. https://doi.org/10.1609/aaai.v32i1.11503.
9. Hutson, M. (2018). Artificial intelligence faces reproducibility crisis. Science *359*, 725–726. https://doi.org/10.1126/science.359.6377.725.
10. Heil, B.J., Hoffman, M.M., Markowetz, F., Lee, S.I., Greene, C.S., and Hicks, S.C. (2021). Reproducibility standards for machine learning in the life sciences. Nat. Methods *18*, 1132–1135. https://doi.org/10.1038/s41592-021-01256-7.
11. Gundersen, O.E., Cappelen, O., Mølnå, M., and Nilsen, N.G. (2025). The unreasonable effectiveness of open science in ai: A replication study. In Proceedings of the 39th AAAI Conference on Artificial Intelligence, *39*, pp. 26211–26219. https://doi.org/10.1609/aaai.v39i25.34818.
12. Raff, E. (2019). A step toward quantifying independently reproducible machine learning research. In Proceedings of the 33rd International Conference on Neural Information Processing Systems, *32*, H.M. Wallach, H. Larochelle, A. Beygelzimer, F. D'Alché-Buc, and E.M. Fox, eds. (Curran Associates Inc.), pp. 5485–5495.
13. Korbmacher, M., Azevedo, F., Pennington, C.R., Hartmann, H., Pownall, M., Schmidt, K., Elsherif, M., Breznau, N., Robertson, O., Kalandadze, T., et al. (2023). The replication crisis has led to positive structural, procedural, and community changes. Commun. Psychol. *1*, 3. https://doi.org/10.1038/s44271-023-00003-2.
14. Simons, D.J. (2014). The value of direct replication. Perspect. Psychol. Sci. *9*, 76–80. https://doi.org/10.1177/1745691613514755.
15. Popper, K. (1934). The Logic of Scientific Discovery (Julius Springer).
16. Shapin, S., and Schaffer, S. (1985). Leviathan and the Air-Pump: Hobbes, Boyle, and the Experimental Life, Revised Edition (Princeton University Press).
17. Kotz, S., Balakrishnan, N., Read, C.B., Vidakovic, B., and Johnson, N.L. (2005). Encyclopedia of Statistical Sciences*4* (John Wiley & Sons).
18. Koo, T.K., and Li, M.Y. (2016). A guideline of selecting and reporting intraclass correlation coefficients for reliability research. J. Chiropr. Med. *15*, 155–163. https://doi.org/10.1016/j.jcm.2016.02.012.
19. Gebru, T., Morgenstern, J., Vecchione, B., Vaughan, J.W., Wallach, H., III, H.D., and Crawford, K. (2021). Datasheets for datasets. Commun. ACM *64*, 86–92. https://doi.org/10.1145/3458723.
20. Stodden, V., Seiler, J., and Ma, Z. (2018). An empirical analysis of journal policy effectiveness for computational reproducibility. Proc. Natl. Acad. Sci. USA *115*, 2584–2589. https://doi.org/10.1073/pnas.1708290115.
21. Claerbout, J.F., and Karrenbach, M. (1992). Electronic documents give reproducible research a new meaning. In Proceedings of the 62nd Annual International Meeting of the Society of Exploration Geophysics, pp. 25–29. https://doi.org/10.1190/1.1822162.
22. Gent, I., and Kotthoff, L. (2014). Recomputation.org: Experiences of its first year and lessons learned. In Proceedings of the IEEE/ACM 7th International Conference on Utility and Cloud Computing (IEEE), pp. 968–973. https://doi.org/10.1109/UCC.2014.158.
23. Schwab, M., Karrenbach, N., and Claerbout, J. (2000). Making scientific computations reproducible. Comput. Sci. Eng. *2*, 61–67. https://doi.org/10.1109/5992.881708.
24. Buckheit, J.B., and Donoho, D.L. (1995). Wavelab and reproducible research. In Wavelets and Statistics, *103* (Springer), pp. 55–81. https://doi.org/10.1007/978-1-4612-2544-7_5.
25. Association for Computing Machinery (2020). Artifact review and badging. https://www.acm.org/publications/policies/artifact-review-and-badging-current.
26. National Academies of Sciences, Engineering, and Medicine (2019). Reproducibility and Replicability in Science (The National Academies Press).
27. Gundersen, O.E. (2021). The fundamental principles of reproducibility. Phil. Trans. R. Soc. A. *379*, 20200210. https://doi.org/10.1098/rsta.2020.0210.
28. Raff, E., Benaroch, M., Samtani, S., and Farris, A.L. (2025). What do machine learning researchers mean by "reproducible"? In Proceedings of the 39th AAAI Conference on Artificial Intelligence, *39*, pp. 28671–28683. https://doi.org/10.1609/aaai.v39i27.35093.
29. McCarthy, J., Minsky, M.L., Rochester, N., and Shannon, C.E. (2006). A proposal for the Dartmouth summer research project on artificial intelligence: August 31, 1955. AI Mag. *27*, 12–14. https://doi.org/10.1609/aimag.v27i4.1904.
30. Goodman, S.N., Fanelli, D., and Ioannidis, J.P.A. (2016). What does research reproducibility mean? Sci. Transl. Med. *8*, 341ps12. https://doi.org/10.1126/scitranslmed.aaf5027.
31. Crook, S.M., Davison, A.P., and Plesser, H.E. (2013). Learning from the past: Approaches for reproducibility in computational neuroscience. In 20 Years of Computational Neuroscience, J.M. Brower, ed. (Springer), pp. 73–102. https://doi.org/10.1007/978-1-4614-1424-7_4.
32. Poldrack, R.A. (2019). The costs of reproducibility. Neuron *101*, 11–14. https://doi.org/10.1016/j.neuron.2018.11.030.
33. Snelleman, T., Hoos, H.H., and Gundersen, O.E. (2026). Cost of Reproducibility Repository. Zenodo. https://doi.org/10.5281/zenodo.21032062.
34. Gundersen, O.E., Gil, Y., and Aha, D.W. (2018). On reproducible ai: Towards reproducible research, open science, and digital scholarship in ai publications. AI Mag. *39*, 56–68. https://doi.org/10.1609/aimag.v39i3.2816.
35. Gundersen, O.E., Shamsaliei, S., and Isdahl, R.J. (2022). Do machine learning platforms provide out-of-the-box reproducibility? Future Gener. Comput. Syst. *126*, 34–47. https://doi.org/10.1016/j.future.2021.06.014.

Patterns, Volume *7*

Supplemental information

The cost of reproducibility in artificial intelligence

Thijs Snelleman, Holger Hoos, and Odd Erik Gundersen

S1. Supplemental Methods

Due to the length of the full guidelines document, we included it in our online repository: https://zenodo.org/records/21032062.

The guidelines consist of 11 pages, including two example reviews with snippets of actual papers as examples, and explain numerous possibilities for which the cost can be increased in bullet points with phrases the reviewer may be looking for in the study. We formulated the criteria based on previous reproducibility quantification studies, such as Gundersen and Kjensmo [S1], Raff [S2] and various individual reproducibility studies, as well as personal experience. We validated the generalisability of the criteria across various subfields of AI research in a preliminary experiment, adjusting them based on encountered issues in practice. We analyse the coverage of our protocol over a set of unseen case studies — reproducibility issues identified in literature that were not considered during development of our protocol — in subsection S2.2.

S1.1 Criteria Examples

To provide further details on the methodology and illustrate the application of our criteria, we provide examples on how they are to be applied. Each criterion of the protocol is stated with a description, followed by various examples, stating the cost increase and a hypothetical situation that may be encountered in empirical AI publications. The complete protocol can be found in the supplementary material.

S1.1.1 Implementation Cost

Criterion: Installation Instruction & Execution [Code Available]

The degree to which the source code is documented for installation and execution.

Example [+0]: Upon code inspection, the repository contains a `README.md`, a `requirements.txt` file, and a shell script to reproduce the main results table.
Example [+1]: Upon code inspection, the repository contains a `requirements.txt` file, but no run instructions making it more costly for the independent investigator to use the code.
Example [+1]: Upon code inspection, the repository contains a `README.md` file, explaining how to call the entry point of the code, but no details on how to install making it more costly for the independent investigator to execute the code on their machine.
Example [+2]: The code is available but has no description on installation notes or how to run the code making it more costly for the independent investigator to get started with applying the code overall.

Criterion: Repository Documentation [Code Available]

The degree to which the source code itself is documented for understanding and navigation

---

Example [+0]: The repository is large, but the authors provide a `README.txt` indexing each folder such that specific files can easily be found. Methods and classes are provided with a brief but comprehensive `docstring` making everything easily accesible to the independent investigator.
Example [+1]: The repository structure is simple, and is easily indexed by the file or directory names, but apart from copyright `docstrings`, the code basically has no comments making it more costly for the independent investigator to understand.
Example [+1]: The repository structure is overly complex, and the file names are not very straightforward to understand, but each method has a clear `docstring` describing the input arguments, making the code easy to follow once the correct file has been found. This makes it more costly for the independent investigator to explore and oversee the repository.
Example [+2]: The repository structure is extremely nested and many files in each directory. Some may be taken from other implementations and pasted in here. Apart from a random comment here and there, the repository is a maze to navigate and understand making it more costly for the independent investigator to use and trust.

21

Criterion: Practical details [No Code, +4]

The degree to which the paper documents design and implementation choices; can the article can be used as a blueprint for the code?

Example [+0]: The authors state programming languages, but also specifically state which methods and classes they used from other specific packages to build their method on giving a good starting point for the independent investigator to implement the method.
Example [+1]: The article states the programming language and packages used with their version, but also mention specific issues they ran into and how they optimised their code or created a work around giving a starting point for the independent investigator but may be a costly endeavour by having to resolve certain practical issues.
Example [+2]: The article has some mention on the implementation details and specifically states their solution was developed using `Python 3.11` with the latest version of the `JAX` package. The independent investigator can rely on specific tools from the package but will still have incur the cost of reimplementing the entire method.
Example [+3]: The article makes little mention of how they executed, but states they developed their solution in `C++`. The independent investigator has a starting point for their search, but most of the work will be very costly for them.
Example [+4]: The article makes no mention of how they executed their method, and sticks to formal and mathematical definitions. The independent investigator to start the costly process of translating the entire methodology from definition to application from scratch.

22

Criterion: Pseudo Code and Overviews [No Code, +4]

The degree to which the methodology itself is easy to oversee.

Example [+0]: The authors have summarised their new algorithm in pseudo code in the appendix, making the stepwise procedure of the method easy to follow for the independent investigator.
Example [+0]: The authors provide a overview figure showing how their new Neural Network architecture diverges from previous works, functioning as a blueprint of sort for the independent investigator.
Example [+1]: The authors develop a new algorithm to be calculated within a Neural Network architecture. They provide pseudo code on said method, but an overview on this new architecture is not provided. The independent investigator will have pay the cost of piecing together an overview from the article before reimplementing the method.
Example [+2]: The authors provide no overview or summary of their methodology in a structured format, leaving the independent to the costly process of deducing everything from mathematical or formal definitions. 23

Criterion: Alternative Implementation Documentation [No Code, +4]

The authors find alternative ways to document critical points of the implementation.

Example [-2]: The authors provide not only pseudo code, but also paste simplified code examples of a specified programming language and framework/package in which they executed their method. Hence, a reduced version of the code is provided, substantially reducing the cost for the independent investigator as they can immediately start with something resembling an implementation. 24

S1.1.2 Data Cost

Criterion: Dataset

To which degree a used dataset is documented.

Example [+0]: The authors use a public dataset, provide a link where it is to be downloaded and cite the original paper introducing the dataset. There is a description on what the data represents and details on the number of features and instances, making all information surrounding the data easily accessible to the independent investigator.

Example [+1]: The authors use a public dataset, providing a citation and a link, and state how many graphs there are in the dataset including the median amount of edges and nodes. There is no explanation on what the data represents or why it was selected for evaluation of the methodology, making it more costly for the independent investigator to understand why this dataset was selected.

Example [+2]: The authors describe the benchmark dataset, providing statistics and its purpose for evaluation but do not cite the source or a link where it can be found making it more costly for the independent investigator to find the right (version) of the dataset.

Example [+2]: The authors cite the dataset and provide a download link, but provide no other information making it costly for the independent investigator to understand the data.

Example [+3]: The authors code automatically download the dataset used. There is no mention on the dataset in the article except for its name making the dataset costly to understand for the independent investigator.

Example [+4]: The authors state they use a benchmark dataset, but other than the name, it is up to the reader to determine meta information leaving the independent investigator with a costly search and possibly many assumptions.

Example [+5]: The authors use a private dataset. They state the collection strategy step wise in the appendix, but provide little statistics or descriptions on the resulting dataset. The independent investigator knows how re-create the dataset, but is left with the cost of defending whether their dataset is similar (enough).

Example [+5]: The authors use a private dataset. They state briefly how it was collected and why it remains private, but release a subset of the private dataset for evaluation purposes. The independent investigator has some working data, but is still left with the cost of acquiring auxiliary data to supplement the private data.

Example [+6]: The authors use a private dataset with a summary on how it was collected. They provide some form of description of the data but no statistics on for example the resulting size. The independent investigator is left with the cost of either filling in the gaps of the acquisition and executing it, or making assumptions on the presented dataset and defending these assumptions when present results on an alternative public dataset.

Example [+7]: The authors use a private text sentiment dataset, with a note that it was 'sourced from a popular forum'. It is not clear how many dialogues this resulted in leaving the independent investigator with the cost to fill in the gaps.

Example [+8]: The authors state a description of what their private dataset represents. No other information. The independent investigator has a high cost to replace it.

Example [+9]: The authors state they use an internal dataset, which they only name with next-to no details, forcing the independent investigator to make a myriad assumptions.

Criterion: Synthetic Data

To which degree generated data is documented.

Example [+0]: The authors use a synthetic data generator to create various mathematical functions to model. They describe and visualise them in the paper, stating the exact function and parameter values. The actual generator is provided in the code, making it easy for the independent investigator to understand the process and generate new data.
Example [+0]: The authors use a simulated environment and cite the package they used where it is implemented. The environment does not take any parameters. The independent investigator can easily access the environment and use it.
Example [+1]: The authors use a public environment simulator, which is cited and described in the paper. They state how many steps they use per experiment, but not which difficulty parameters are used exactly to generate the levels leaving the independent investigator with the cost to explore or assume the values.
Example [+2]: The authors use a public simulator, which installs with the code. There is no information however on what the problem is to be solved by the method, leaving the cost with the independent investigator to understand what the method is actually evaluated on.
Example [+3]: The authors use a public synthetic data generator, which automatically installs with their code. They do not specify which parameter settings they use per experiment to create the synthetic dataset, forcing the independent investigator to pay the cost of exploring or assuming the values.
Example [+4]: The authors use a synthetic data generator which is included in the code, but provide little explanation and do not document the majority of the parameter values used for generation, leaving a substantial cost with the independent investigator.
Example [+5]: The authors use a synthetic data generator which is included in the code. They do not explain on what the generated data is supposed to represent or what parameter values must be used to generate the dataset but do provide some visualised examples allowing the independent investigator to have some insights on the generator but still leaves a costly process for re-creating.
Example [+6]: The authors use a synthetic data generator, providing the functions and parameter values but do not share the implementation, leaving it to the independent investigator to re-implement.
Example [+7]: The authors use a private environment, describing clearly how it is parametrised but not what the task is. It is not clear which value is used for the parameters per experiment. This makes the reimplementation a costly endeavour but once implemented the values to be used are clear.
Example [+8]: The authors use a private synthetic data generator which they state is parameter has three input parameters. They provide details on the task or on the generation process, but omit any information on the parameter values. The requirements for reimplementation are clear to the independent investigator, but a costly implementation process and parameter search remains.
Example [+9]: The authors use a private generator which is only named. The independent investigator is left with the entire cost to start from scratch under an array of assumptions.

Criterion: Dataset averaging

How multiple datasets are to be unified into a single cost.

Example [+1]: The authors use five benchmark datasets and provide all information except for a link. Each is represented in the main results Table 1. They are hence weighted equally as each is as costly to use for the independent investigator.
Example [+2]: The authors use four datasets, three of which are public datasets used to benchmark the method against other state-of-the-art methods. They provide an auxiliary experiment on their internal dataset, which they briefly describe (+8) — only to demonstrate its real world performance. Hence the majority of conclusions are made on the public dataset, leading to this dataset being weighted half of the others; $\frac{1+1+1+9}{2+2+2+1} = \frac{12}{7} \approx 2$. The independent investigator is left with some cost although most of the conclusions are accessible for reproduction.

We note that the above criterion on dataset averaging, in contrast to other criteria in this dimension, is quite subjective; nonetheless, it was deemed necessary for enabling the annotator to create a unified cost representative of the entire study. This criterion of applying different weights to the datasets was applied once throughout the study; during the secondary review, one reviewer scaled two datasets to $\frac{1}{3}$ of the weight of six other datasets. This resulted in a cost difference of one between the two annotators.

S1.1.3 Configuration Cost

Criterion: (Hyper)parameters

The explicit listing of all (hyper)parameters considered for the method.

Example [+0]: The authors provide pseudo code stating the input parameters of their method. It is immediately clear to the independent investigator what the parameters are.
Example [+1]: The authors discuss the parameters used with description in text, although a structured overview is missing leaving the independent investigator with the cost to verify the parameters throughout the article.
Example [+2]: The authors discuss certain parameters in the text, but it is unclear whether these were all the parameters considered for the method. The independent investigator is left with the cost to search through the article, code, and possible cited works to verify the parameters.
Example [+3]: The authors perhaps discuss some parameters throughout the article, but a real definition is not found, leaving the costly process of extracting and assuming information by the independent investigator.

Criterion: (Hyper)parameter values

The actual (hyper)parameter values used for the experiments.

Example [+0]: The authors provide a `configuration.yaml` per experiment in the code repository, making each value easily recognisable.
Example [+1]: The authors provide a hyperparameter table specifying the values, although not each completely overlaps with their parameter definition/overview leaving a small cost of verification for the independent investigator.
Example[+2]: The authors provide a concise table stating the hyperparameters of their method, but it is unclear to which experiment they belong and a substantial amount of values are missing; the independent investigator pays the cost of filling in the gaps.
Example[+4]: The authors provide no structured overview on the hyperparameter values and only detail this briefly for some in text. The independent investigator is left with the cost of reacquiring the majority of the values. 37

Criterion: (Hyper)parameter value acquisition

How the authors acquired the (hyper)parameter values used.

Example [+0]: The authors state they use the parameters of previous works to remain comparable to other methods in their benchmark, citing the papers.
Example [+1]: The authors state they empirically chose the values in a preliminary experiment, leaving it unclear what kind of experimentation budget was used leaving the independent investigator unsure how to reliably reacquire comparable values.
Example [+2]: The authors state the simply the values used, leaving it unclear to the independent investigator how to pay the cost of supplementing (possible missing) values in a comparable fashion. 38

S1.1.4 Experimental Procedure Cost 39

Criterion: Metrics

The metrics used for the evaluation of the method's performance on the task.

Example [+0]: The authors use standard metrics such as accuracy and F1-score. They also use more complex metrics for which they provide a citation or describe and denote the formula in the appendix.
Example [+1]: The authors use a mixture of metrics, although some require domain knowledge and are not specified. For a few they provide a citation, but for others it will require the independent investigator to survey external documentation to understand how these are calculated.
Example [+2]: The authors use complicated metrics which are only named; the independent investigator is left with the cost to verify their meaning and how they were applied. 40

Criterion: Data partition

The specification on which data the evaluation is presented.

Example [+0]: The authors specify they use the train/validation/test split of the dataset and denote for each result whether it was on the validation or test set.
Example [+1]: The authors state on which dataset the result is produced on, and state dataset is split randomly into training, validation and testing 70/10/20. However, they don't state on which split their tables and figures report on, leaving the cost of determining this to the independent investigator.
Example [+2]: The authors state their results are on the test set, but do not clarify how they separate the benchmark data into training and testing. The independent investigator has to assume a comparable protocol.
Example [+3]: The authors clearly use a training procedure in their methodology and thus implying a possible train/test split, but in the results they only state the dataset they are reporting on. The independent investigator is left with the cost of assuming the protocol and on which split to compare to. 41

Criterion: Strategy

The strategy used to evaluate the method on the data.

Example [+0]: The authors state they use k-fold cross validation with $k = 10$ for each dataset.
Example [+0]: The authors state they run each experiment with five random seeds
Example [+1]: The authors present the results over 'multiple seeds', but the amount of seeds is unclear. The independent investigator has to assume and defend a budget.
Example [+2]: The authors present the results with mean and standard deviation over a population of 5, but it is unclear *what* strategy produced this population of results. The independent investigator has to assume how to recreate the population of the experiment.
Example [+3]: The authors present the results with a median value and a 95% confidence interval, but do not specify under what strategy they present this population nor what the population size is. The independent investigator now has to pay the cost of searching and assuming alternatives for both. 42

Criterion: Aggregation of results

Is it clear what kind of statistics the authors are presenting on their results?

Example [+0]: The authors state their results as $a \pm b$ and denote in the caption of the table that this is the mean and standard deviation.
Example [+1]: The authors state their results in the table are presented with the mean but do not clarify what the variation statistics describes. The independent investigator has to pay the cost of finding a suitable replacement.
Example [+2]: The authors provide a graph with their results, comparing their methodology against multiple state-of-the-art methods. The graph contains a centre-line and a shaded area, but no clarification on what these represent. The independent investigator has to pay the cost of assuming and replace both aggregator and variation. 43

S1.2 Discussion

During the study, we found that the data dimension shows somewhat of an ‘artificial inflation’; when the authors used popular or ‘standard’ datasets, such as MNIST [S3] or CIFAR [S4], the authors did not feel the need to report detailed information on them, especially when it does not play a central role to the method. We decided against making exceptions for popular or common datasets as we found it too complicated to objectively decide what makes a dataset common or ’popular’, especially when dealing with smaller subfields that may use less well known datasets to the broader community, but may be considered common in their respective subfields. Thus we did not adopt a separate procedure for these datasets. This also indicates the authors did not provide a (detailed) motivation for choosing a standard dataset for a given task.

We also note that in a few cases, the number of papers slightly varied per venue or year. This we accounted for due to one conflict of interest detected post-review, and the others due to a sampling incident regarding pre-selecting the award-winning papers, resulting in more reviews.

Furthermore, we noted one theoretical example where the implementation cost could exceed ten by our guidelines; we have not seen any instance where this occurred.

For the statistical analysis we considered many statistical tests to determine the significance between distributions, which we would like to clarify. The normality test showed that the data is not normally distributed ($p < 0.01$ for all). Using Brown-Forsythe test, with the venues as groups, we find that implementation and experimental procedure have strong evidence of non-homogeneity ($p < 0.01$), significant evidence for configuration (0.045) but not for data (0.30). We did not apply ANOVA as we would violate two assumptions: Normality and homogeneity. We applied the Kruskal-Wallis test instead: For each dimension, there is a statistical significant difference for at least one group ($p < 0.003$ for all). We considered the following tests for comparing venues per dimension:

- Kolmogorov-Smirnov: Non-parametric test, does not assume normality distribution. However, it does not have a one sided version.
- Friedman: requires repeated measures which we do not have.
- Wilcoxon signed rank: an alternative to the t-test when normality cannot be assumed, but requires paired observations
- Mann-Whitney U / Wilcoxon rank-sum: no normality assumption, does not require same size samples or paired data but assumes equal variance
- Permutation: No assumption about normal distribution or variances. For robustness, we increased resamplings to 1 000 000

We decided against p-value correction (Bonferonni) to reduce the false positive rate, as our hypothesis is per single test rather than using a family of statistical test for a single hypothesis, and instead opted for interpretation of the results for practical relevance.

S1.3 Assumptions on the entire population

Our analysis is widely aimed at comparing venues, and hence our sampling strategy was conducted uniformly at random per venue per year, with the same sample sizes. However, in a few cases, we also presented findings across the entire population; the average public data rate, average implementation link rate and its subsequent error rate. As our sampling strategy does not take into account the proportionality of each venue to the population, we weighted these metrics to the *estimated* rates by determining the proportion of empirical publications of each venue per year and calculating their contribution to the averages accordingly. This resulted in the following value shifts in our study:

- The estimated public data rate, measured in our dataset at 93.95%, calculated to be 94.01% after weighing the sources per year.

- The estimated public implementation rate, measured in our dataset 62.13%, corrected to 70.52%% after weighing the sources per year. This is the most substantial change, due to the substantially lower rates found in JMLR/JAIR, whose impact is more limited to the overall population than other venues due to a lower amount of publications per year.

- The estimated implementation URL error rate, measured in our dataset at 6.59%, corrected to 6.72%.

S1.4 Secondary Review

A highly important part of this work is the secondary review to determine the reproducibility of our methodology. There are a few details that we would like to highlight with regards to these results. We have acquired our secondary reviewers through our research network and considered doctoral candidates, post-docs and professors based on their proficiency. To ensure a diverse group of secondary reviewers, we used various channels to contact a population with a diverse background. For example, the research directions of our network varies strongly; among the secondary reviewers we recorded the following primary research directions: Reinforcement Learning, Neural Network Verification, Music Information Retrieval, Robustness, Algorithm Selection/Configuration, Multi-Agent systems, Time Series Analysis, SAT Solving, Hyperparameter Optimisation, Data Mining, and, ML for Earth Observation. Thus, the communities they are part of which impact their views and judgements are diverse. This was done to reflect a wide variety of researchers from the field of AI, and mitigate possible biases in this part of the dataset. We subsampled our dataset using the same strategy as our initial sampling, and ensured each venue and year received a proportional amount of reviews to be representative of the distribution of the dataset.

The secondary reviewers were allowed to select papers by title themselves based on their expertise and personal interest. This potentially introduced a topic-matching bias — but this was permitted for two main reasons; 1) expertise in a certain subfield tends to increase the consistency of a given rater across samples and 2) their interest in the domain reduces the chances of human error due to fatigue. As Bayerl and Paul [S5] pointed out, raters with similar levels of expertise may give rise to inflated agreement scores. They note that this bias, the influence of expertise on agreement, is not consistent across domains. As we do not have any information whether this bias is applicable to our domain, this remains an open question. However, the topic-matching bias only occurred on one side of the annotation — namely the secondary reviewer. As noted by Bayerl and Paul [S5]: "[..] to increase the likelihood of high agreement rates annotator groups should consist of coders with a comparable level of expertise". Hence, we argue that the topic-matching bias triggered the exact opposite effect — only one side was matched with their supposed expertise, whereas the other was sampled uniformly at random, thus not guaranteeing any specific subfield expertise.

Another important subject of this analysis is the sample size; we acquired 46 secondary reviews for our entire dataset (5.01%). Although larger sample sizes are always highly desirable in any data-driven study, we faced resource constraints due to the complexity of the task; we had strong requirements for the academic expertise of the reviewers, and conducting such a review is time consuming. We do find the sample size is sufficient for statistically robust claims, and hence do not expect this outcome to change substantially with a larger sample size under this strategy.

S2. Extended Results

Apart from annotating a sample of publications over the past three years of seven main venues, we also validated our methodology against actual reproduction attempts. We first discuss limitations on our comparison with the dataset by Gundersen et al. [S6] in subsection S2.1, then discuss an extension of this dataset with reproducibility studies sourced from TMLR in subsection S2.2, analyse the intra-annotator agreement in subsection S2.3, and discuss the possible enrichment of meta data in subsection S2.4.

S2.1 Correlation with Gundersen et al. (2025)

In the dataset of Gundersen et al. [S6], there are a several sources of bias that should be noted with regards to our analysis:

- The authors used publications from a different time frame (2011 to 2016). Standards of documentation have changed over the years, which may bias the data.
- The authors considered *only* publications that use public data for their experiments.
- The authors selected the most highly cited publications from their time frame.
- The authors used publications from other venues than those included in this study. Although our method is generalised towards all empirical AI studies, this may imply a shift in population as the community (standards) of researchers from these venues may vary from those evaluated in this work.
- The authors have decided on a hard cut off of forty working hours per study. Working hours also included computing time; this is not considered labour by the definition of reproducibility cost in our work.

Limitations of our method. Gundersen et al. [S6] measured problem types P1, ..., P20 per reproduction attempt, but not present per study in the original publication. We requested the data regarding which study encountered which problem type, which they kindly provided us with. They encountered twenty different problems, the majority of which is covered in the documentation evaluation of our method; in fifteen cases, we could determine that the subject is covered in our assessment of at least one dimension. However, five of these cases (P1, P7, P14, P19 and P20) are not covered, which amounts to 18.84% of the encountered issues. This is a limitation of our method (i.e. encountered documentation issues that are not captured by our methodology) that we would like to discuss.

- (P1) “Method code is shared, but not experiment code”: Our methodology focuses on the documentation of the implementation of the newly presented method. The authors find this issue in five attempts; only one occurred in an unsuccessful attempt.
- (P7) “Random seeds and random number generators not specified”: We explicitly decided against including such values into our cost assessment, as we believe that outcomes should generally be statistically robust and reproducible under repetition with different pseudo-random number sequences. Gundersen et al. [S6] found this issue in four attempts, none of which resulted in a failure to reproduce a given study. Hence we conclude that not including this into our measurement is not a limitation, but rather that our choice is supported by the (limited) data set.

- (P14) “The article contains an error”: This is a very important problem, that can only be detected upon deeper inspection (and perhaps actual reproduction attempt) of a study. Gundersen et al. [S6] encountered this once, but the reproduction attempt was still successful.
- (P19) “Results are presented in a way that makes a comparison hard”: This problem is focused on the presentation of the method. This is a very important topic that is hard to capture objectively. Gundersen et al. [S6] encountered this in two publications; the reproduction attempt of one turned out to be unsuccessful.
- (P20) “Lack of access to hardware or software needed to conduct the experiment”: This problem is not related to the documentation provided by authors, but rather the accessibility of resources and thus not included in our method, based on our definitions.

S2.2 Analysis on TMLR Reproducibility Studies

The correlation study between the reproducibility effort of Gundersen et al. [S6] has yielded valuable insights into the relation between our methodology and actual reproduction attempts. Although the study by Gundersen et al. [S6] has involved substantial effort to acquire data on the reproducibility of AI research, a larger population sample may afford more statistical power and achieve more generalisable conclusions. To this end, we analysed reproducibility studies from the Transactions on Machine Learning Research (TMLR). TMLR is an online open access journal (https://jmlr.org/tmlr/).

S2.2.1 Acquisition strategy

TMLR awards special certifications to submissions aiming to reproduce a previous study, a characteristic we leveraged to manually scrape the submission website (TMLR uses OpenReview for a public reviewing process; https://openreview.net/group?id=TMLR). When collecting possibly relevant studies, we specifically included in our sample studies that have been rejected; without, this may introduce a bias towards certain studies, for example failed reproduction attempts that were submitted to TMLR. However, to ensure we only rely on methodologically sound reproducibility studies, we selected them based on the feedback of the reviewers and the editor. Hence, only those rejected due to a ‘lack of novelty’ — as reproducibility studies are required by TMLR standards to go “beyond simple verification” and to “contribute significant added value through additional baselines, analysis, ablations, or insights.” (See; https://jmlr.org/tmlr/editorial-policies.html) — were excluded from our dataset.

This resulted in 39 reproducibility studies published in TMLR between 2024 and 2026. We subsequently extracted the reproduced papers of each reproducibility study from the abstract and labelled the original papers according to our methodology. Some to-be-reproduced articles that were selected by the papers in our TMLR dataset were published outside of our scope of venues, such as ACL and SIGIR, although the vast majority (84.62%) appeared at the venues we included in our original study, such as AAAI, ICML, NeurIPS and ICLR. After labelling the original studies, we sought to apply the labels of Gundersen et al. [S6] to this new dataset, running into several limitations:

- Reproducibility studies submitted to TMLR have very high expectations in terms of effort by the independent investigators; more often than not, the papers have more than one author, and, as they are expected to extend upon the original study in addition reproducing it, it is not unreasonable to assume this exceeds the forty working hour limit of a single individual used by Gundersen et al. [S6].

- The increase of budget has implications on how we can apply our methodology to this extended dataset. Our work focuses on estimating the amount of unnecessary labour for independent reproduction due to lacking documentation; this means that a TMLR study finding that a claim is not reproducible more likely than not will indicate some methodological flaw in the original work than an 'inability' of the independent investigators to reproduce it due to lacking documentation. This entails, in the scope of our work, that the independent investigators in our sample will likely have 'paid the cost', even if it was unreasonably high.

- A smaller concern is that Gundersen et al. [S6] labelled reproduction attempt in terms of experiments reproduced, whereas TMLR publications usually divide up the elements of the reproduction in terms of claims made by the original authors.

Based on the reasons stated above, we adapted our original protocol towards the problem types Gundersen et al. [S6] encountered in their work. Besides labelling the claims aimed to be reproduced and the results thereof, we also inspected each study for issues encountered during the attempt at reproducing earlier work. As the scope of each TMLR reproduction study is larger than those considered by Gundersen et al. [S6], we extended the problem types with *ten* new labels, as shown in Table S1.

ID	Source	Description of Problem	Covered
P21	Code	Installation of the code was difficult to execute	Implementation
P22	Data	The generator or simulator code was not provided or available	Data
P23	Code	Code was not shared at all	Implementation
P24	Contact	Contact with the authors was necessary and yielded more information	Yes
P25	Contact	Contact with the authors was necessary but received no (useful) response	Yes
P26	Code	A bug was detected in the code	No
P27	Article	It is unclear how the hyperparameter value was selected	Configuration
P28	Data	Part of the data requires a key	Data
P29	Resources	The code is inefficient and requires optimisation	No
P30	Resources	Running the experiments requires a substantial amount of compute	No

Table S1: Extension of the problem classification by Gundersen et al. [S6]. The first column shows the new problem ID, the second column a source description as done in Gundersen et al. [S6], the third column the description of the problem, and the last column the dimension of our methodology covering the problem. Note that for P24 and P25, we simply show 'yes', as this covers various questions about lacking or ambiguous documentation. For the definition of the first twenty problem types, please see Gundersen et al. [S6] or our repository.

As shown in Table S1, our methodology covers seven out of ten new problems independent investigators encountered during their reproduction attempts. With regards to P29 and P30, these are mainly to allow for more detailed expressions of issues of compute than P20. P26, a bug was detected in the code, has also not been covered by our methodology, as these are usually only discovered at runtime rather than inspecting the code documentation for completeness and quality. With these new labels, we joined the two datasets resulting in a total of *61* reproduction attempts.

S2.2.2 Analysis

In Figure S1, we quantify the occurrences of each problem type in the extended dataset, sorted by how it is covered by our methodology.

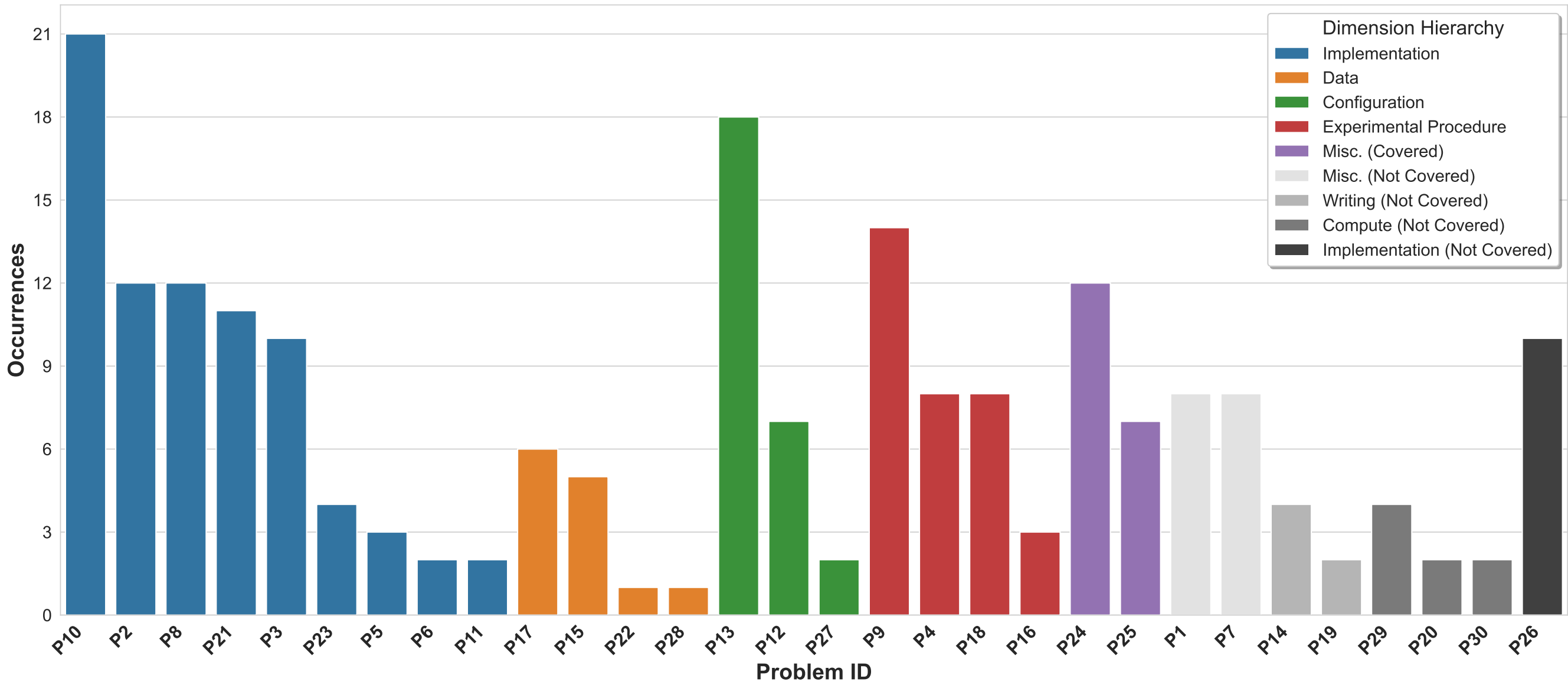

Figure S1: The amount of occurrences of each problem type in the reproducibility study dataset, sorted, left to right, based on their coverage by our methodology dimensions (Implementation, Data, Configuration, Experimental Procedure), followed by those that are not covered in grey and black. It is directly apparent that P10 (21), P13 (18), P9 (14) and P24 (12) are occurring most frequently. Overall, 80.86% of the reported problems are captured by our methodology.

Based on the findings in Figure S1, our method captures a vast majority of the encountered issues in practice. Those that are not represented in our methodology are largely explainable due to the fact that they have little or no overlap with the documentation responsibilities of the authors.

However, there some important details we would like to emphasise. Firstly, the most frequently encountered problems (P10, P13, P9 and P24) are captured strongly by our methodology and associated with substantial increases in reproducibility cost. Secondly, there are few issues encountered with the data dimension; only in two studies did independent investigators record such problems (P22, P28), although this was largely biased by the fact that Gundersen et al. [S6] considered only studies with public data. We found this to be the case for the TMLR studies as well: only two datasets used throughout 39 reproducibility studies were subject to (partial) data privacy, one of which was resolved after direct contact with the authors. The other data problem was an issue related to code provided by original authors using a data scraper with a privatised API requiring an API key (P28).

In our original analysis we found that the majority of published AI research relies on the usage, yet still found that 3.99% of AI publications rely on solely private data. Yet in our TMLR dataset we only find a singular case of an issue with (semi) private data, showing a possible bias of reproduction attempts towards studies with public data. This bias may suggest that a significant share of published research is not even attempted to be reproduced — an estimated 3.99% of all published research based on our data set. The overuse of public benchmark datasets may lead to over-fitting to specific instances of a given problem or task, which may have detrimental effects on performance beyond those limited use cases [S7] — possibly affecting the reproducibility of conclusions that presume the results to be generalisable or transferable to other instances or tasks.

Furthermore, we found in the 19 cases in the TMLR data where independent investigators sought

contact with original authors (P24, P25) — Gundersen et al. [S6] explicitly avoided contact with the authors: "We did not contact the authors as the documentation should contain all necessary information." In 63.16% of the reported cases, the original authors responded. This gives rise to a form of semi-public information; the authors clearly omitted some crucial information required by independent investigators, yet this was not included directly in the original publication. This elucidates the need for concise and comprehensive documentation — especially with known decay rates of the correctness of contact details found by Vines et al. [S8], showing that author contact is an unreliable method of information acquisition. This is unreliability of contacting authors is found in our TMLR dataset as well; over one third of the attempts at obtaining important information from original authors were unsuccessful.

Lastly, the TMLR data also highlights a possible limitation of our methodology; a frequently occurring problem in these reproducibility studies were related to the installation procedure of code provided by original authors (P21, eleven occurrences). This was captured by our protocol; a cost increase of one was attached if no instructions were provided at all on how to install the code (e.g. dependencies, packages, versioning). However, we found that in at least two studies this was provided, yet independent investigators still encountered this problem. Nonetheless, we believe this is still fairly represented in our methodology; the authors clearly made an effort to document the procedure, and they cannot be expected to ensure that it works across all conceivable target platforms, let alone stand the test of time with the speed at which software dependencies change and software is deprecated. Nonetheless, this issue illustrates that our approach opens the door to alternative cost schemes that may further refine how the problem is extracted. For example, a protocol based on empirical data could serve as a strong alternative foundation. However, as such data is extremely costly to create, we believe such an effort is a study on its own and thus leave this for future work.

Furthermore, the frequency of installation issues raises a new question: "How can we as a community improve the best practices when it comes to code installation documentation?" Our observation here is supported by the empirical study of Samuel and Mietchen [S9], who found that only 10 388 notebooks within their dataset of 22 578 Python notebooks successfully installed 'out of the box' using a requirements file, even though 15 817 notebooks had their dependencies declared in a standard file. Thus, in only 70.1% of the cases the installation is properly documented, but only in 65.6% of the 70.1% cases Samuel and Mietchen [S9] were able to execute the code. This means that Samuel and Mietchen [S9] found an effective installation rate of 46.0%, which demonstrates the frequency and scale of this problem.

Furthermore, the artifact decay rate does not only affect code but also data, and its subsequent impact on the cost of reproducibility, must not be underestimated. As reported by Vines et al. [S8], the availability of data artifacts from published studies declines by 17% per year post-publication.

S2.3 Intra-annotator analysis

As discussed previously, a limitation of our study is the lack of reviewing resources. Although we validated our methodology by studying the agreement between various reviewers on the same publications, we have not yet investigated possible inconsistency of a single reviewer on this dataset — the within-person inconsistency or error [S5]. To measure this, we randomly sampled three previously annotated samples from each venue and year resulting in 63 publications. We excluded those previously labelled as *theoretical* from sampling, as we draw our conclusions from and aim our actionable findings at empirical research. Furthermore, we have omitted expertise from this experiment, as we have already concluded that our methodology was not sufficiently grounded to accurately capture this dimension.

In a preliminary analysis, we immediately detected a strong outlier; this was determined to be the result of our review on "Three rates of convergence or separation via U-statistics in a dependent

framework", published in JMLR in 2022. The root cause for this extreme intra-annotator variation (an increase of 7 in implementation) was determined to be a deleted GitHub repository, where the authors originally hosted their implementation. This is an example of data-drift rather than intra-annotator variation, and has therefore been excluded from our subsequent analysis. Nonetheless, this outlier serves as a clear-cut example of the dark pattern regarding 'dead' implementation URLs. We continued our analysis with a sample size of 62, representing 6.75% of our full dataset.

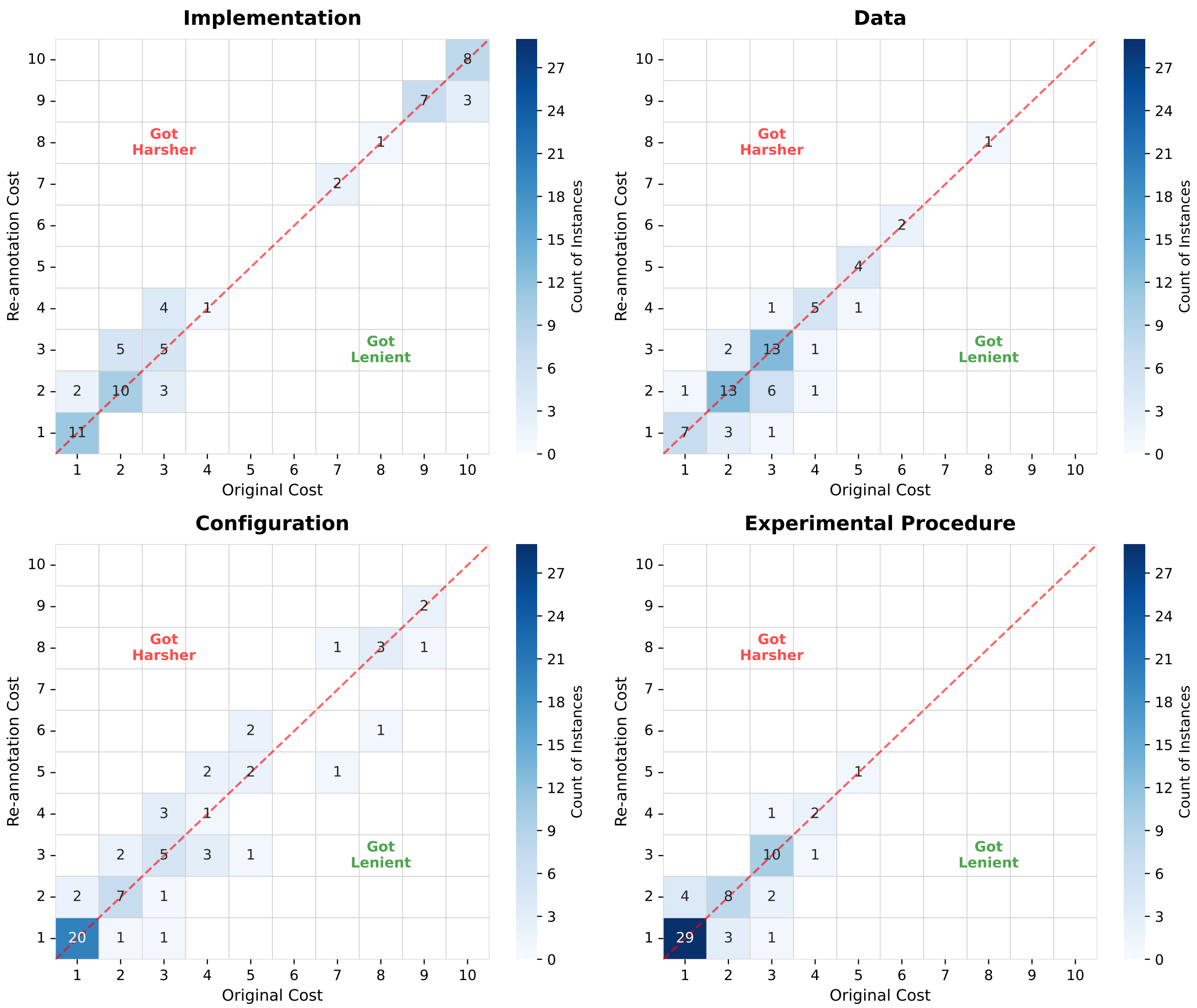

Figure S2: Confusion matrix of the original labels produced by the reviewer (x-axis) and a second review conducted by the same reviewer several months later (y-axis) per dimension. The colour intensity indicates the frequency of the occurrences, which are labelled per cell with their exact values. The diagonal line indicates exact overlap (agreement), whereas below the diagonal suggest labels have drifted to lower costs (i.e. the reviewer 'became lenient'), and above the diagonal suggest labels have drifted to high costs (i.e. the reviewer 'became harsher').

In Figure S2, we present the results of our investigation of within-reviewer inconsistency in the form of a confusion matrix. Overall, the scores have changed in a few cases, most frequently in the data and configuration categories, with inconsistencies up to two points, but mostly limited to one. They are also relatively spread out across the main diagonal indicating perfect consistency. We noticed one exception: studies that previously received a cost of three now frequently receive a cost of two. Based on our methodology, this is likely caused by the rule set regarding the descriptions of

data; cost may increase when the description of the dataset used or the statistics regarding the data are lacking. What constitutes 'sufficient' descriptions or statistics is relatively subjective, and this is reflected in the data presented here. Nonetheless, our analysis indicates overall low within-reviewer inconsistency.

	MAD	ICC	95% CI	P-Value	Label
Implementation	0.27	0.989	[0.98, 0.99]	0.000	Excellent
Data	0.31	0.908	[0.85, 0.94]	0.000	Excellent
Configuration	0.42	0.953	[0.92, 0.97]	0.000	Excellent
Experimental Procedure	0.21	0.885	[0.82, 0.93]	0.000	Good

Table S2: The intraclass correlation coefficient (ICC) over the original and re-evaluation review with 95% confidence interval, P-Value and their interpreted labels based on Koo and Li [S10].

To statistically assess the within-reviewer inconsistency, we calculated the intraclass correlation coefficient, as seen previously in the main article. The results are shown in Table S2, calculated using the ICC-1 model, as in this scenario, we have a single rater assessing the same items twice. We find excellent agreements for implementation, data and configuration dimensions, and a good agreement for the experimental procedure. The P-values for each test are less than 0.0001, easily passing our threshold of 0.05 for statistical significance. To conclude, although some reviewer inconsistency has been detected, this effect is minor and thus does not significantly impact our analysis or conclusions.

S2.4 Enriching the Data Set

To expand the data set and open up more angles of analysis, we leveraged SCOPUS — the curated abstract and citation database of Elsevier — through the tool from Rose and Kitchin [S11] to yield more meta-data on each evaluated paper. Although promising, we have gained little insights thus far using these extra features. However, we did find one interesting point, using the amount of citations per paper; when analysing the relationship between citation count and our dimensions of cost we found it difficult to determine any consistency between these variables. Thus, we determined the Kendall correlation coefficient between each dimension per year and the number of citations provided by SCOPUS. We calculated the correlation per year, as more recent papers have had less opportunity to receive citations than older papers. The results can be found in Table S3.

# Citations	Implementation	Data	Configuration	Exp. Procedure
2022	**-0.10**(0.02)	-0.08(0.06)	**-0.10**(0.02)	0.01(0.85)
2023	-0.06(0.15)	-0.03(0.53)	0.02(0.60)	0.02(0.64)
2024	-0.05(0.31)	-0.07(0.12)	**0.10**(0.02)	0.05(0.29)

Table S3: The Kendall Correlation between the number of citations and the dimensions per calendar year. A negative correlation implies that lower cost correlates with a higher amount of citations. The statistically significant results are marked in **bold**.

Three results in Table S3 have been found to be statistically significant, however the correlation across the board are quite weak with the largest value being 0.10. Thus, deducing any relation between these variables based on this data is very weakly supported. Hence, we find in this exploration no signals that *better documented* studies receive more citations. As citations are a key metric to measure the success of a publication, this illustrates that, although the community finds high reproducibility standards important, applying best practices with regards to documentation has little effect on how it is received on the short (3 year) term. However, there is a caveat to this; we

have only collected data for the past three year and can see indications of an up tick of correlation year-on-year in for example implementation and configuration. Hence, it may be hypothesised that, although high quality documentation may be unrewarding on the short term, on the long term it will survive the test of time. We leave such investigations for future work.

S3. Extended Background

In this work, we use the term 'reproducibility' to refer to a general scientific concept, rather than limiting it to the field of AI or computer science, for which we find two possible standards: Goodman et al. [S12] and Gundersen [S13], who both used quite similar definitions and terminology. Goodman et al. [S12] separated the definitions of reproducibility into three types: methods, results and inferential reproducibility, which partially overlap with the definitions in Gundersen [S13], who defined three degrees of reproducibility: outcome, analysis and interpretation reproducible. Both Goodman et al. [S12] and Gundersen [S13] presented their types of reproducibility within a hierarchical ordering. Goodman et al. [S12] referred to methods reproducibility as the ability to repeat the procedure of the study on the level of implementation, e.g. "methodologically reproducible", where the experimental procedure can be repeated using the documentation provided. This does not include the outcome or the conclusions that are drawn from the experiment. We note a discrepancy between fields: in psychology, methodologically reproducible would mainly refer to reproducing an experimental set-up, whereas in computer science, this would also refer to the reproducibility of the implementation or execution, e.g. by running code. Although the other categories and degrees, such as results reproducibility and outcome reproducibility or inferential and interpretation reproducibility, overlap more between these two approaches, for conciseness and clarity, we refrain from using definitions of categories and degrees. We find, however, the definitions by Goodman et al. [S12] to be more ambiguous, as they lack a definition of independent investigators ('reproducers'), as well as the concept of what constitutes the documentation of a method.

We also disagree with the reasoning that the search for 'truth' is the main motive for reproducibility, and argue that in the field of AI, it is rather the credibility of the method [S14]. This discrepancy between definitions likely originates in the epistemological nature of both fields; the framing by Goodman et al. [S12] reflects an approach that more closely models the discovery of knowledge in the natural world — in AI, we rather consider the discovery of knowledge, at least to some degree, to occur by determining and comparing the performance of some methodologies for solving a given problem or accomplishing a given task. Hence, by reproducing such findings, we add credibility to specific methodologies.

Crook et al. [S15] discussed various approaches regarding reproducibility in computational neuroscience. They defined reproduction and replication of an experiment: An independent investigator either reproduces an experiment, or replicates it 'using the same code', and they consider the latter to be 'certainly easier than independent reproduction'. Later, they redefined these terms as internal and external replicability, and introduced the concept of cross-replicability: The ability to simulate the same model with different software. Lastly, they defined reproducibility as the ability to implement a model independently, without using the source code provided by the original authors.

We refrain from using these definitions, due to their ambiguity and limitations. The definition of reproducibility excludes the source code as part of the documentation, which we find an unnecessary and somewhat unnatural constraint. The terms of internal and external replicability are redundant from our point of view: Reproducibility should be measured based on the documentation made publicly available by original authors, and should thus not vary based on the relation to the independent investigator; one could even argue that a closer relation could create a conflict of interest. The information provided to a reader for 'external replicability' should be the same as for reproducibility. Lastly, the term cross-replicability refers to the ability to re-implement a method

outside the domain of the source code. We see this as a constrained version of the re-implementation of methods within reproducibility.

S3.1 Alternative costs

Alternatively to documentation quality, other types of reproducibility cost can be conceived. For example, one could focus on resource requirements in terms of monetary cost. A famously expensive example from physics involves CERN's Large Hadron Collider [S16], a ten-year project with a 4.75 billion USD price tag [S17]. Reproducing research that requires such an instrument would be unrealistic, unless it is made available to independent investigators, and may also imply other costs, in terms of effort (for securing access) or time (the resource is in high demand). The issue of resource requirements is becoming more prevalent in AI research, e.g., in the context of large deep learning models. Large language models (LLMs), such as Open AI's ChatGPT-4 and Microsoft's Copilot models, take several months to train on thousands of state-of-the-art GPUs. Tian et al. [S18] conducted a reproducibility study of AlphaZero [S19] and found that "A single training run requires [..] days of training on thousands of TPUs, which is an unattainable level of compute for the majority of the research community". The authors further state: "When combined with the unavailability of code and models, the result is that the approach is very difficult, if not impossible, to reproduce, study, improve upon, and extend". High demand for compute can be accepted as an "irreducible cost" by the original authors, as it is in general not in their reach nor their responsibility to make these resources available, whereas lack of documentation is clearly a "reducible cost" that could be mitigated relatively easily. Yet, as we have seen in subsection S2.2 and Figure S1 specifically, the high demand for computing resources is a frequently reported problem in reproducibility studies. Hence, the issue of compute with substantive implications for reproducibility which we leave for future work.

S4. Extended Quantification Studies

In Raff [S2], the author defines features to test for correlation with reproducibility of studies. The most directly linked feature to our work is the specification of hyperparameters, which we evaluate under configuration cost, and pseudo code, which we evaluate under implementation cost. It is important to note that both of these features are considered in Raff [S2] as 'mildly subjective', which also finds its place in our work as we enable our reviews to diverge from the set guidelines. One significant feature that we do not include in our work is required compute. We have chosen to exclude this, as our focus lies on the documentation provided by authors rather than the accessibility of compute. Another reason, unrelated to the work of Raff [S2], is that the variation that can be caused by different low-level systems (such as a CPU/GPU architecture or drivers) is more closely aligned with *outcome* reproducible rather than *interpretation* reproducible, e.g. being able to draw the same conclusion. Variations in (low-level) system software may impact the results outcome in unexpected ways, even if factors of stochasticity are well documented; for example seeds for non-deterministic algorithms. However, exactly reproducing the outcomes is a highly strict view on reproducibility which often may be unnecessary; as long as outcomes support the same interpretations for the hypothesis (for example, varying outcomes but statistical tests still prove the same significances) we find this sufficient to determine a study reproducible.

Due to its widespread applicability and popularity, the impact of irreproducible machine-learning research is not limited to its field [S20–S22]. In Kapoor and Narayanan [S23], the authors study 642 publications across 17 scientific fields, ranging from medicine, satellite imaging and cybersecurity to the political sciences. They identified 294 cases of data leakage and classified them into eight categories. These classifications cover situations such as ill-separated training and test sets, or the

lack thereof entirely, as well as more complicated examples such as temporal leakage or feature selection and dataset pre-processing on the test set. The authors conclude that the application of ML methods has many pitfalls and that each is rediscovered independently by each community. To prevent or mitigate these issues, Kapoor et al. [S24] provides recommendations for a stronger review process regarding reproducibility documentation, which can help to alert the authors to their mistakes, thus contributing to more realistic or well-founded outcomes. The authors conclude that this identification process of these pitfalls is a first step in the direction of addressing this interdisciplinary crisis. Secondly, increasing the focus on documentation on the application of AI methods regarding reproducibility emphasises the importance to the community.

S5. Extended figures and tables

In this section, we present several graphs and tables that are used as a supporting role for our main results. In Table S4 we present the median, quantiles and data skew. In Table S5 we represent the results of the normality test. In Table S6 we show the permutation test between all sources per dimension, testing which sources are significantly less costly than others. In Table S7 we present the results of the Kolmogorov-Smirnov test and permutation test for award winning papers. In Figure S3 we present the correlation per source and overall.

	Implementation					Data				
	Q1	Median	Q3	IQR	Skew	Q1	Median	Q3	IQR	Skew
AAAI	2	6.50	9	7	-0.09	1	2.00	3	2	1.35
IJCAI	2	5.00	9	7	0.14	1	2.00	4	3	1.47
ICLR	2	2.00	8	6	0.86	2	3.00	4	2	1.31
ICML	1	2.00	8	7	0.72	1	3.00	3	2	0.80
NeurIPS	2	2.00	5	3	1.16	2	3.00	4	2	0.92
JAIR	2	3.00	9	7	0.32	2	3.00	4	2	0.87
JMLR	2	3.00	10	8	0.17	2	3.00	4	2	0.46

	Configuration					Experimental Procedure				
	Q1	Median	Q3	IQR	Skew	Q1	Median	Q3	IQR	Skew
AAAI	2	3.50	5	3	0.95	1	2.00	3	2	1.41
IJCAI	2	3.00	5	3	1.07	1	2.00	3	2	1.57
ICLR	2	3.00	4	2	1.64	1	2.00	3	2	1.34
ICML	1	3.00	3	2	1.73	1	1.00	3	2	1.05
NeurIPS	3	3.00	4	1	1.36	1	2.00	3	2	0.85
JAIR	1	2.50	4	3	1.35	1	1.00	2	1	1.68
JMLR	1	3.00	4	3	1.18	1	1.00	2	1	1.80

Table S4: The median, first and third quantile and interquantile range of each dimension per venue. The skewness of the distribution is nearly always positive, with the exception of AAAI in the implementation dimension, thus motivating the representation of median rather than mean as it is frequently substantially affected by right skewed distribution outliers except for the implementation dimension which has relatively mild skew values.

	Implementation	Data	Configuration	Experimental Procedure
AAAI	1728.65 (0.00)	35.00 (0.00)	17.19 (0.00)	36.18 (0.00)
IJCAI	1398.58 (0.00)	36.10 (0.00)	18.00 (0.00)	43.33 (0.00)
ICLR	29.38 (0.00)	37.34 (0.00)	51.78 (0.00)	34.83 (0.00)
ICML	72.84 (0.00)	12.35 (0.00)	52.38 (0.00)	18.42 (0.00)
NeurIPS	20.97 (0.00)	22.03 (0.00)	34.03 (0.00)	12.82 (0.00)
JAIR	3948.80 (0.00)	10.70 (0.00)	23.84 (0.00)	39.46 (0.00)
JMLR	1075.33 (0.00)	8.91 (0.01)	21.25 (0.00)	48.88 (0.00)

Table S5: Normality test [S25] of all venues and dimensions, where the p-values are shown in brackets. The results are statistically significant for each venue. Thus, we find evidence for each venue that the results are not normally distributed.

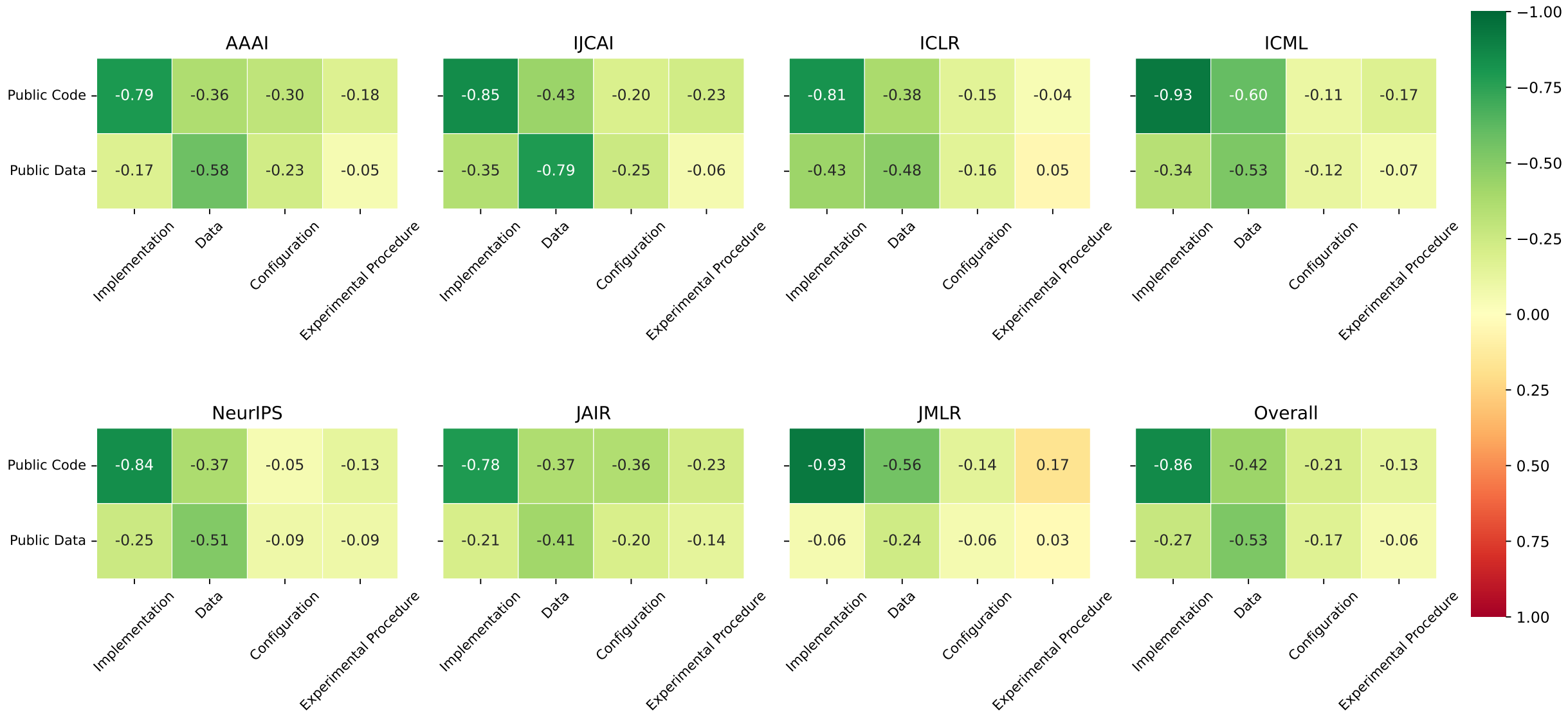

Figure S3: Kendall Correlation heat map of the dimensions and the collected metrics, namely implementation link (URL) and proportion of public data sets (Public Data). A negative correlation indicates lower cost.

S6. Data Collection

In the sampling process, we manually excluded the following categories of papers from each source, as they generally lack a full empirical evaluation:

- In AAAI we exempted the (student) abstracts
- In IJCAI we exempted the survey, doctoral consortium, and early career tracks. The Special Track on AI for Good (Projects) and 'demonstration track' were curated for empirical evaluations. 'Extended abstract' papers were included when the full version was linked.
- In ICLR, ICML and NeurIPS we exempted papers originating from a journal.
- In ICML we curated Position Papers for an empirical evaluation.

In our data scraping, we supplemented the data from OpenReview manually to ensure representative documentation from the point of view of a reviewer for each paper. We have two specific

notes: JMLR 2024 only had 49 accepted papers at the time of sampling, thus all were used. In JAIR 2022 "Marginal Distance and Hilbert-Schmidt Covariances-Based Independence Tests for Multivariate Functional Data" where code is available upon request. We requested, received, and reviewed accordingly. Regarding the reviews of papers with awards, we included from each source/year the papers with 'best paper award' up to 8 papers. If more were presented with such an award 8 were sampled at random due to resource and time constraints and to avoid data undersampling of the 'regular' paper population.

During sampling, the following papers evaluated for possible conflict of interest:

- IJCAI 2023 we rejected "Solving the Identifying Code Set Problem with Grouped Independent Support" due to the first author being a former PhD candidate of one of the authors
- IJCAI 2023 we still evaluated "One Model, Any CSP: Graph Neural Networks as Fast Global Search Heuristics for Constraint Satisfaction" as although the researchers come from the same institution we have no direct contact with them
- ICML 2023 we rejected "Expectation-Complete Graph Representations with Homomorphisms" due to a personal connection of the first author to the authors.
- ICML 2022 we still evaluated "Markov Chain Monte Carlo for Continuous-Time Switching Dynamical Systems" even though the code is hosted RWTH Aachen (No direct affiliation, authors are from TU Darmstadt)
- NeurIPS 2022 we rejected "JAHS-Bench-201: A Foundation For Research On Joint Architecture And Hyperparameter Search" as HH has publications with Frank Hutter
- JMLR 2024 we rejected "Critically Assessing the State of the Art in Neural Network Verification" as one of the authors also authored this paper
- JMLR 2022 we rejected "SMAC3: A Versatile Bayesian Optimization Package for Hyperparameter Optimization" due to personal connections with the authors.
- JAIR 2024 we rejected "A Hybrid Intelligence Method for Argument Mining" due to a personal connection to the authors
- JAIR 2024 we rejected "Improving Reproducibility in AI Research: Four Mechanisms Adopted by JAIR" as it was authored by the authors
- JAIR 2023 we rejected "MDP Playground: An Analysis and Debug Testbed for Reinforcement Learning" due to a personal connection with one of the authors
- JAIR 2022 we rejected "FOND Planning with Explicit Fairness Assumptions" due to a connection with the third author.

Implementation

	AAAI	IJCAI	ICLR	ICML	NeurIPS	JAIR	JMLR
AAAI	-	0.81	1.00	1.00	1.00	0.90	0.77
IJCAI	0.20	-	1.00	0.99	1.00	0.67	0.46
ICLR	**0.00** (-1.66)	**0.00** (-1.27)	-	0.34	0.62	**0.01** (-1.06)	**0.00** (-1.33)
ICML	**0.00** (-1.47)	**0.01** (-1.08)	0.67	-	0.77	**0.03** (-0.88)	**0.01** (-1.14)
NeurIPS	**0.00** (-1.77)	**0.00** (-1.38)	0.40	0.25	-	**0.01** (-1.18)	**0.00** (-1.44)
JAIR	0.10	0.34	0.99	0.97	1.00	-	0.31
JMLR	0.24	0.55	1.00	0.99	1.00	0.70	-

Data

	AAAI	IJCAI	ICLR	ICML	NeurIPS	JAIR	JMLR
AAAI	-	0.14	0.10	0.28	**0.02** (-0.42)	0.15	**0.00** (-0.67)
IJCAI	0.87	-	0.49	0.74	0.25	0.55	**0.05** (-0.42)
ICLR	0.91	0.54	-	0.79	0.24	0.58	**0.03** (-0.40)
ICML	0.74	0.29	0.24	-	0.06	0.30	**0.00** (-0.55)
NeurIPS	0.98	0.77	0.78	0.95	-	0.82	0.12
JAIR	0.87	0.48	0.45	0.73	0.20	-	**0.03** (-0.44)
JMLR	1.00	0.96	0.97	1.00	0.89	0.98	-

Configuration

	AAAI	IJCAI	ICLR	ICML	NeurIPS	JAIR	JMLR
AAAI	-	0.79	1.00	1.00	0.95	1.00	0.96
IJCAI	0.22	-	1.00	1.00	0.76	0.95	0.83
ICLR	**0.00** (-1.08)	**0.00** (-0.84)	-	0.55	**0.00** (-0.64)	0.20	**0.03** (-0.53)
ICML	**0.00** (-1.10)	**0.00** (-0.86)	0.48	-	**0.01** (-0.66)	0.19	**0.04** (-0.55)
NeurIPS	0.06	0.26	1.00	1.00	-	0.89	0.65
JAIR	**0.01** (-0.83)	0.05	0.82	0.82	0.12	-	0.23
JMLR	**0.04** (-0.55)	0.19	0.97	0.97	0.37	0.78	-

Experimental Procedure

	AAAI	IJCAI	ICLR	ICML	NeurIPS	JAIR	JMLR
AAAI	-	0.83	0.97	1.00	0.98	1.00	1.00
IJCAI	0.20	-	0.83	0.99	0.86	1.00	1.00
ICLR	**0.04** (-0.30)	0.20	-	0.91	0.56	0.98	0.99
ICML	**0.00** (-0.48)	**0.02** (-0.32)	0.11	-	0.11	0.81	0.92
NeurIPS	**0.03** (-0.32)	0.17	0.49	0.91	-	0.98	1.00
JAIR	**0.00** (-0.58)	**0.01** (-0.43)	**0.03** (-0.28)	0.23	**0.03** (-0.27)	-	0.70
JMLR	**0.00** (-0.65)	**0.00** (-0.49)	**0.01** (-0.34)	0.11	**0.01** (-0.33)	0.35	-

Table S6: Permutation test of 1 000 000 resamples between sources per dimension, where each row is tested to be smaller than each column. The table presents the found p-values per comparison/test. The statistically significant results are highlighted in **bold** with the statistic values (distances) in brackets.

**Implementation**	KS Test	P-Test
AAAI	0.20 (0.99)	0.42 (0.63)
IJCAI	0.35 (0.31)	2.28 (0.95)
ICLR	0.14 (0.94)	-0.06 (0.48)
ICML	0.18 (0.51)	-1.09 (0.11)
NeurIPS	0.15 (0.81)	-0.41 (0.31)

**Data**	KS Test	P-Test
AAAI	0.13 (1.00)	0.07 (0.54)
IJCAI	0.14 (0.99)	0.39 (0.70)
ICLR	0.14 (0.93)	-0.24 (0.33)
ICML	**0.33** (0.03)	**-1.12** (0.00)
NeurIPS	0.22 (0.34)	-0.65 (0.06)

**Configuration**	KS Test	P-Test
AAAI	0.28 (0.86)	-0.79 (0.28)
IJCAI	**0.64** (0.00)	2.43 (1.00)
ICLR	0.13 (0.96)	-0.34 (0.26)
ICML	0.17 (0.64)	-0.77 (0.08)
NeurIPS	0.20 (0.44)	0.99 (0.99)

**Exp. Proc.**	KS Test	P-Test
AAAI	0.43 (0.36)	-0.53 (0.27)
IJCAI	0.26 (0.68)	0.64 (0.94)
ICLR	0.14 (0.94)	-0.22 (0.27)
ICML	0.10 (0.98)	-0.25 (0.18)
NeurIPS	0.10 (0.99)	-0.25 (0.20)

Table S7: Kolmogorov–Smirnov test [S26] and Permutation test per source and dimension between papers with and without awards. The P-values are in brackets. The statistically significant results are highlighted in **bold**.

References

S1. Gundersen, O.E., and Kjensmo, S. (2018). State of the art: Reproducibility in artificial intelligence. In Proceedings of the 32nd AAAI Conference on Artificial Intelligence vol. 1. pp. 1644–1651. doi: https://doi.org/10.1609/aaai.v32i1.11503.

S2. Raff, E. (2019). A step toward quantifying independently reproducible machine learning research. In Proceedings of the 33rd International Conference on Neural Information Processing Systems vol. 32. pp. 5485–5495.

S3. LeCun, Y., Bottou, L., Bengio, Y., and Haffner, P. (1998). Gradient-based learning applied to document recognition. Proceedings of the IEEE *86*, 2278–2324. doi: https://doi.org/10.1109/5.726791.

S4. Krizhevsky, A., and Hinton, G. (2009). Learning multiple layers of features from tiny images. Computer Science Department, University of Toronto. URL: http://www.cs.utoronto.ca/~kriz/learning-features-2009-TR.pdf Accessed: 01-07-2026.

S5. Bayerl, P.S., and Paul, K.I. (2011). What determines inter-coder agreement in manual annotations? a meta-analytic investigation. J. Comput. Linguist. *37*, 699–725. doi: https://doi.org/10.1162/COLI_a_00074.

S6. Gundersen, O.E., Cappelen, O., Mølnå, M., and Nilsen, N.G. (2025). The unreasonable effectiveness of open science in ai: A replication study. Proceedings of the 39th AAAI Conference on Artificial Intelligence *39*, 26211–26219. doi: https://doi.org/10.1609/aaai.v39i25.34818.

S7. Sourget, T., Akkoç, A., Winther, S., Galsgaard, C.L., Jiménez-Sánchez, A., Juodelyte, D., Petitjean, C., and Cheplygina, V. (2024). [citation needed] data usage and citation practices in medical imaging conferences. In N. Burgos, C. Petitjean, M. Vakalopoulou, S. Christodoulidis, P. Coupe, H. Delingette, C. Lartizien, and D. Mateus, eds. Proceedings of the 7th International Conference on Medical Imaging with Deep Learning vol. 250 of *Proceedings of Machine Learning Research*. pp. 1475–1496.

S8. Vines, T.H., Albert, A.Y., Andrew, R.L., Débarre, F., Bock, D.G., Franklin, M.T., Gilbert, K.J., Moore, J.S., Renaut, S., and Rennison, D.J. (2014). The availability of research data declines rapidly with article age. Curr. Biol. *24*, 94–97. doi: https://doi.org/10.1016/j.cub.2013.11.014.

S9. Samuel, S., and Mietchen, D. (2024). Computational reproducibility of jupyter notebooks from biomedical publications. GigaScience *13*, 1–23. doi: https://doi.org/10.1093/gigascience/giad113.

S10. Koo, T.K., and Li, M.Y. (2016). A guideline of selecting and reporting intraclass correlation coefficients for reliability research. J. Chiropr. Med. *15*, 155–163. doi: https://doi.org/10.1016/j.jcm.2016.02.012.

S11. Rose, M.E., and Kitchin, J.R. (2019). pybliometrics: Scriptable bibliometrics using a python interface to scopus. SoftwareX *10*, 1–6. doi: https://doi.org/10.1016/j.softx.2019.100263.

S12. Goodman, S.N., Fanelli, D., and Ioannidis, J.P. (2016). What does research reproducibility mean? Sci. Transl. Med. *8*, 341ps12–341ps12. doi: https://doi.org/10.1126/scitranslmed.aaf5027.

S13. Gundersen, O.E. (2021). The fundamental principles of reproducibility. Philos. Trans. R. Soc. *379*, 1–15. doi: https://doi.org/10.1098/rsta.2020.0210.

S14. Popper, K. (1934). The logic of scientific discovery. Julius Springer, Hutchinson & Co.

S15. Crook, S.M., Davison, A.P., and Plesser, H.E. (2013). Learning from the past: Approaches for reproducibility in computational neuroscience. In 20 Years of Computational Neuroscience vol. 9 of *Springer Series in Computational Neuroscience* pp. 73–102.. Springer pp. 73–102. doi: https://doi.org/10.1007/978-1-4614-1424-7_4.

S16. Brüning, O., Burkhardt, H., and Myers, S. (2012). The large hadron collider. Prog. Part. Nucl. Phys. *67*, 705–734. doi: https://doi.org/10.1016/j.ppnp.2012.03.001.

S17. Knapp, A. (2012). How much does it cost to find a higgs boson? Forbes. Forbes Inc. URL: https://www.forbes.com/sites/alexknapp/2012/07/05/how-much-does-it-cost-to-find-a-higgs-boson/#1891ccaa3948.

S18. Tian, Y., Ma, J., Gong, Q., Sengupta, S., Chen, Z., Pinkerton, J., and Zitnick, L. (2019). ELF OpenGo: an analysis and open reimplementation of AlphaZero. In K. Chaudhuri, and R. Salakhutdinov, eds. Proceedings of the 36th International Conference on Machine Learning vol. 97 of *Proceedings of Machine Learning Research*. pp. 6244–6253.

S19. Silver, D., Hubert, T., Schrittwieser, J., Antonoglou, I., Lai, M., Guez, A., Lanctot, M., Sifre, L., Kumaran, D., Graepel, T., Lillicrap, T., Simonyan, K., and Hassabis, D. (2018). A general reinforcement learning algorithm that masters chess, shogi, and go through self-play. Science *362*, 1140–1144. doi: https://doi.org/10.1126/science.aar6404.

S20. Ball, P. (2023). Is AI leading to a reproducibility crisis in science? Nature *624*, 22–25. doi: https://doi.org/10.1038/d41586-023-03817-6.

S21. Gibney, E. (2022). Could machine learning fuel a reproducibility crisis in science? Nature *608*, 250–251. doi: https://doi.org/10.1038/d41586-022-02035-w.

S22. La Malfa, E., Petrov, A., Frieder, S., Weinhuber, C., Burnell, R., Nazar, R., Cohn, A., Shadbolt, N., and Wooldridge, M. (2024). Language-models-as-a-service: Overview of a new paradigm and its challenges. J. Artif. Intell. Res. *80*, 1497–1523. doi: https://doi.org/10.1613/jair.1.15865.

S23. Kapoor, S., and Narayanan, A. (2023). Leakage and the reproducibility crisis in machine-learning-based science. Patterns *4*, 1–12. doi: https://doi.org/10.1016/j.patter.2023.100804.

S24. Kapoor, S., Cantrell, E.M., Peng, K., Pham, T.H., Bail, C.A., Gundersen, O.E., Hofman, J.M., Hullman, J., Lones, M.A., Malik, M.M. et al. (2024). Reforms: Consensus-based recommendations for machine-learning-based science. Sci. Adv. *10*, eadk3452. doi: https://doi.org/10.1126/sciadv.adk3452.

S25. D'agostino, R., and Pearson, E.S. (1973). Tests for departure from normality. Biometrika *60*, 613–622. doi: https://doi.org/10.2307/2335012.

S26. Dodge, Y. (2008). The Concise Encyclopedia of Statistics. Springer.
